# LigHTS: Biomimetic Hydrogel Photo‐Fabrication for Imaging‐Based Ultra‐High‐Throughput Screening

**DOI:** 10.1002/advs.78192

**Published:** 2026-10-12

**Authors:** Alessandro Enrico, Sara Rigolli, Julius Zimmermann, Melissa Pezzotti, Eloisa Torchia, Moises Di Sante, Ferdinando Auricchio, Francesco S. Pasqualini

**Affiliations:** ^1^ Department of Civil Engineering and Architecture Synthetic Physiology Lab University of Pavia Pavia Italy; ^2^ Department of Internal Medicine and Medical Therapy Synthetic Physiology Lab University of Pavia Pavia Italy; ^3^ Group of Computational Mechanics and Advanced Materials Department of Civil Engineering and Architecture University of Pavia Pavia Italy

**Keywords:** 1536‐well plates, absorbers, GelMA, mechanobiology, whole‐plate patterning

## Abstract

Imaging‐based ultra‐high‐throughput screening (UHTS) in pharma and biotech still relies on 384‐ and 1536‐well plates whose rigid, flat substrates limit biological fidelity and screening efficiency. Highly biomimetic organoids and organs‐on‐chips improve relevance but at a cost that is incompatible with early screening campaigns. Photopatterned hydrogel scaffolds are potentially scalable, but current approaches lack plate‐scale reproducibility and throughput. To address this gap, we present chemically‐mediated Light‐Intensity Gradients for structured hydrogel functionalization of ultra‐High‐Throughput Screening formats (LigHTS): an all‐optical, in‐well method for parallel photofabrication of biomimetic hydrogels in standard 384‐ and 1536‐well plates. Adding ultraviolet‐light absorber tartrazine to photo‐curable solutions enables hydrogel thickness control by tuning illumination dose without focusing optics. Entire plates are functionalized in parallel with soft (∼1–10 kPa), thin (∼10–200 µm) hydrogels. Simultaneously, film photomasks that encode anisotropies enable control of well‐level gel thickness and topography at UHTS scale. We demonstrate the utility of biomimetic gelatin‐methacrylate‐functionalized UHTS plates using mechanosensitive human cells (HT1080 fibrosarcoma cells, HaCaT epithelial cell colonies, and cardiomyocyte tissues derived from induced pluripotent stem cells), which display stiffness‐ and topography‐dependent phenotypes. Finally, geometric uniformity across the plate meets industry requirements (coefficient of variation <20%), providing a readily available route to enhanced biomimicry in imaging‐based UHTS pipelines.

## Graphical abstract

LigHTS removes a key bottleneck for ultra‐high‐throughput screening (UHTS) by enabling biomimetic hydrogel microenvironments to be manufactured directly in standard multiwell plates. A single, collimated UV exposure with formulation‐tuned light penetration patterns an entire plate with UHTS‐grade uniformity (<20% CV), producing structured GelMA films spanning 10–200 µm and 1–10 kPa for scalable high‐content imaging assays.

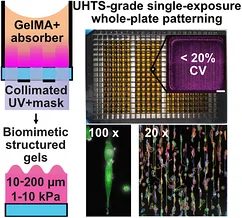

## 1 Introduction

Drug discovery continues to consume capital on late‐stage failures [1, 2, 3]. A key contributor to these failures is the limited predictivity of early‐stage screening models that lack biomimicry. In fact, biomimetic models such as organoids and organs‐on‐chips can improve relevance by restoring key features of the native microenvironment [4, 5], but remain difficult to deploy at the scale, cost, and reproducibility required for early discovery [6, 7, 8] and novel artificial‐intelligence‐based screening campaigns [9, 10, 11]. Consequently, early‐stage development relies on imaging‐based ultra‐high‐throughput screening (UHTS) to test tens of thousands of compounds at pennies per data point using 384‐ and 1536‐well plates [12]. However, these formats are characterized by rigid, flat plastic or glass substrates that poorly reflect the compliant, topographically complex extracellular matrix that shapes tissue structure and function [13, 14]. We believe this is a critical and often underappreciated limitation, as these simplified early models can promote bound‐to‐fail false‐positive compounds to later expensive stages, and even discard promising new therapies as false negatives [15, 16].

In principle, multiwell plates with planar hydrogels and tissue‐like stiffness (Young's modulus ∼1–10 kPa) may offer sufficient biomimicry at the required throughput [17]. Hydrogel thickness in the 10–200 µm range is necessary to decouple cells from the rigid well bottom [18, 19, 20], while remaining compatible with established imaging automation and high‐numerical‐aperture (NA) microscopy [21, 22]. The hydrogel fabrication process must also support instructive anisotropy (e.g., microgrooves) to control alignment and migration [23, 24, 25, 26]. However, existing demonstrations of structured soft substrates in plate‐like formats are limited to the 96‐well scale [27, 28, 29, 30, 31], or rely on custom assemblies or molded bases [32, 33, 34], whereas commercial hydrogel plates typically lack microarchitecture and are too thick for high‐resolution imaging [35, 36]. We previously reported a 384‐well thin‐hydrogel fabrication approach but did not achieve full well coverage or anisotropic microarchitecture [37]. Such microstructures can be fabricated by serial micromolding [38] or optics‐based patterning [39], but these techniques either sacrifice throughput or cannot independently control thickness and topography [40]. In particular, focusing optics coupled with digital micromirror devices (DMD) enforce field‐limited serial stitching and introduce plate‐scale dose nonuniformity [41]. In summary, there is no manufacturing process capable of realizing thin, soft, microgrooved hydrogels in standard 384/1536‐well plates meeting screening‐grade reproducibility and cost requirements.

Here we introduce a plate‐native photofabrication method that shifts thickness control from optics to chemistry, using chemically‐mediated Light‐Intensity Gradients for structured hydrogel functionalization of ultra‐High‐Throughput Screening formats (LigHTS). We reasoned that adding a strong ultraviolet (UV) absorber as a photoinitiator competitor could dominate light attenuation. Radical generation during illumination is no longer dominated by the photoinitiator concentration, allowing cure‐depth tuning with micrometric resolution under collimated illumination [42, 43, 44]. In LigHTS, tartrazine‐containing gelatin‐methacrylate (GelMA) is polymerized directly on the glass bottom of standard 384‐ and 1536‐well plates in a single exposure (Figure 1a), yielding planar films whose thickness is programmable by UV dose tuning (Figure 1b). Interposing simple film photomasks during the same cure step encodes surface anisotropies in the gels with micron‐scale fidelity, providing control of thickness, mechanics, and topography in UHTS formats  (Figure 1c).

**FIGURE 1:**
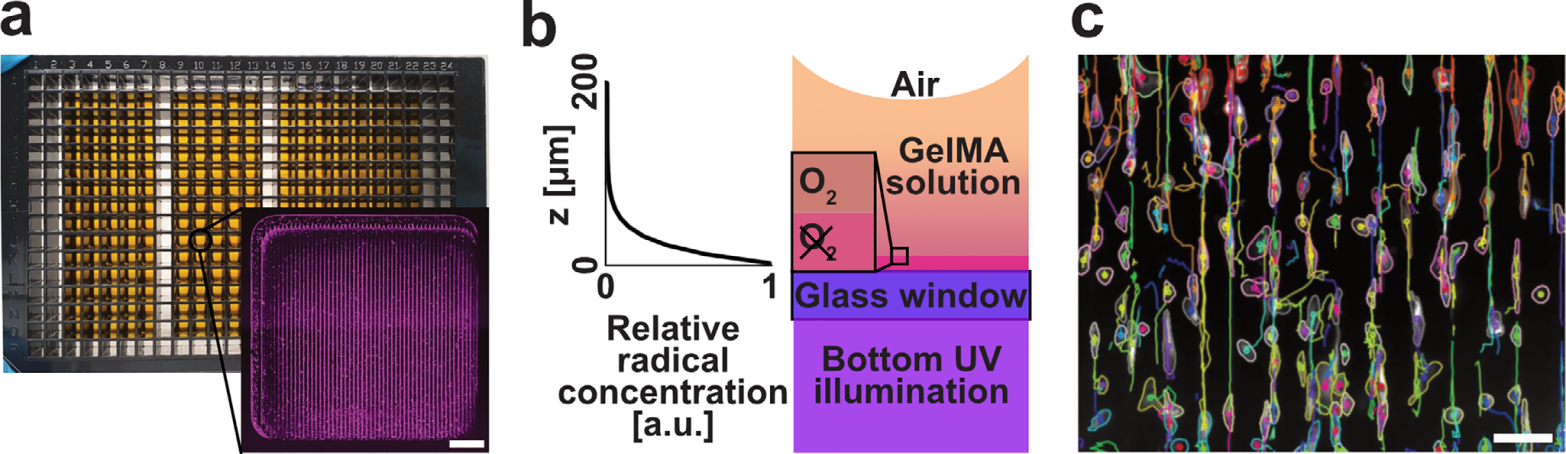
LigHTS: one‐step, light‐patterned approach bringing soft grooved gels to high‐throughput screening. (a) UHTS‐compatible fabrication demonstrated by gel patterning in a 384‐well plate format. A single exposure with collimated UV light (16 minutes) can simultaneously pattern thickness‐controlled (<200 µm) stiffness‐compliant (≈1–10 kPa) grooved gels on the well bottom of UHTS vessels. The method is compatible with the infrastructure and price targets of pharmaceutical UHTS pipelines and requires no post‐patterning integration or assembly steps. Scale bar in the inset: 500 µm. (b) Process principle. A soluble UV absorber is added to a GelMA prepolymer. Following Beer‐Lambert attenuation, the absorber creates a steep exponential decay in transmitted light intensity independent of the photoinitiator concentration. Polymerization occurs only where enough radicals generated by photoinitiator cleavage are present after the oxygen‐dependent induction time. As a result, hydrogel thickness can be controlled by tuning the UV dose and the hydrogel precursor formulation. (c) Imaging‐based phenotypic screening of cells on surface‐structured planar hydrogels. In the presence of grooved gels, HT1080 cells display contact‐guided migration with a preference for the groove main axis. Scale bar: 100 µm.

We validate the approach in four steps: (i) mapping the parametric space for thickness and stiffness definition (Figure 2); (ii) demonstrating high‐NA imaging compatibility and stiffness‐dependent cell phenotypes (Figure 3); (iii) obtaining patterned microgrooves and contact‐guided migration in a DMD illumination configuration (Figure 4), and showing compatibility with other hydrogel chemistries, such as hyaluronic acid methacrylate (HAMA) as well as poly(ethylene glycol) diacrylate (PEGDA); and (iv) translating the process to whole‐plate collimated exposure with plate‐scale metrology and reproducibility benchmarks (Figures 5 and 6). Using mechanosensitive human cells with genetically encoded reporters, including HT1080 fibrosarcoma cells, HaCaT keratinocytes, and cardiomyocytes derived from induced pluripotent stem cells (hiPSC‐CM) [45, 46], we show that LigHTS enables biomimetically relevant, imaging‐ready substrates at a scale and quality control (QC) level aligned with industrial screening (proof‐of‐concept multiparametric perturbation assay in Figure 7) and phenotypic modeling.

**FIGURE 2:**
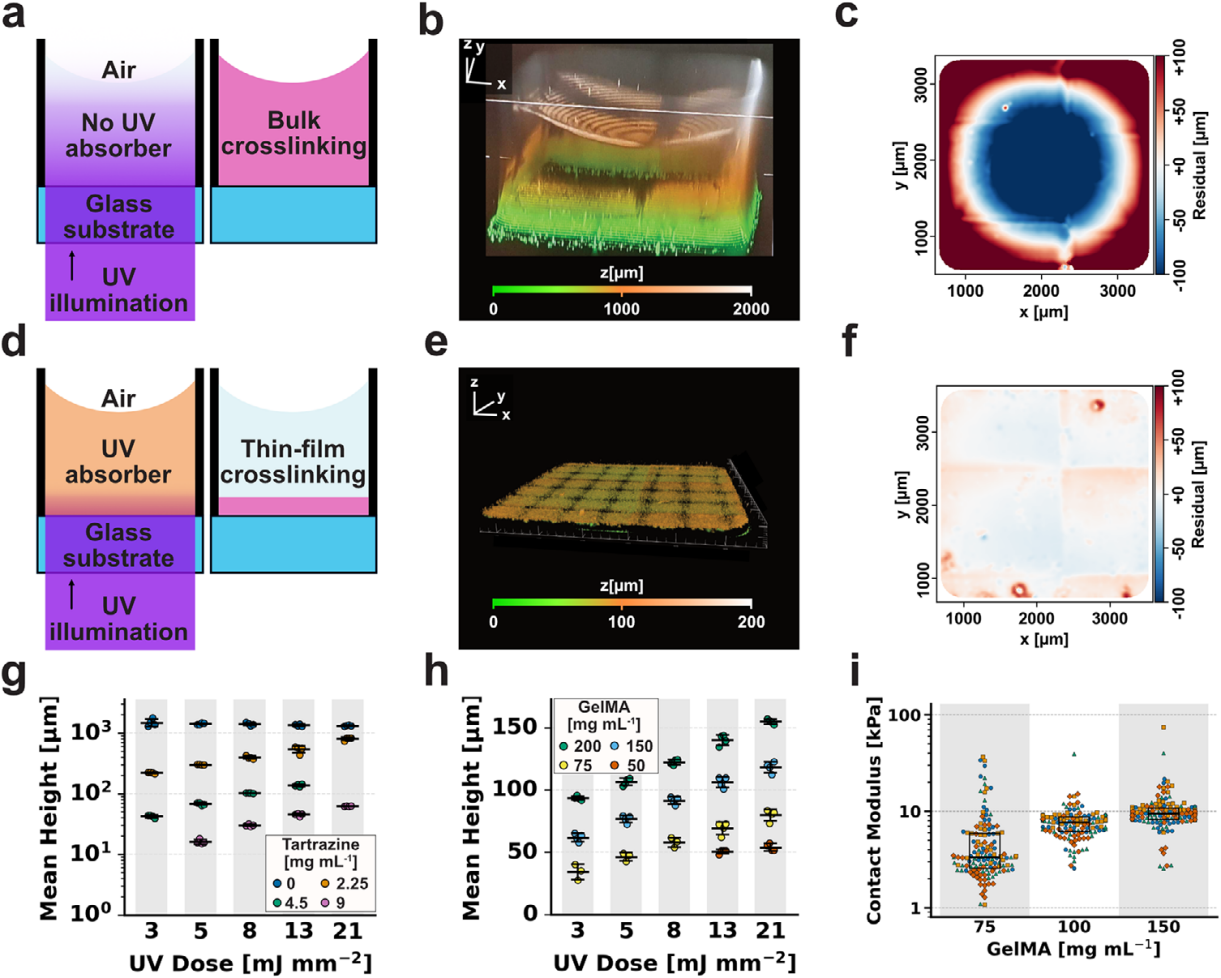
Introducing a strong optical absorber in hydrogel precursor solution enables generating sub‐200‐µm thickness‐tunable flat gels in a loosely‐focused UV exposure configuration. (a) Polymerization dynamics. Full‐volume crosslinking for commercial hydrogel precursor solution in well‐plate formats. When illuminated with a sufficient UV dose, the bulk of the prepolymer solution crosslinks. (b) 3D isometric rendering and (c) 2D residual height map of resulting hydrogels with prominent meniscus (>200 µm center‐to‐wall height difference). The resulting gel thickness largely exceeds 200 µm and correlates with the available volume of solution. The hydrogel surface was coated with fluorescent beads and analyzed with confocal fluorescence microscopy. (d) LigHTS strategy consisting of doping photosensitive hydrogel precursor solution with a strong UV absorber, such as tartrazine, to decouple optical absorption from the ECM and photoinitiator components and enable single‐digit‐micrometer control of the hydrogel thickness. (e) 3D isometric rendering and (f) 2D residual height map of meniscus‐free, flat, and thin hydrogels obtained by LigHTS approach. Tartrazine‐containing solutions result in a 10‐fold decrease in gel thickness inhomogeneity compared to tartrazine‐free formulations. (g) Parametric study of thickness dependence on UV dose and tartrazine concentration for fixed GelMA (75 mg mL^−^^1^) and LAP (2.5 mg mL^−^^1^) concentrations. Hydrogel thickness decreases monotonically with tartrazine concentration at every dose (Jonckheere–Terpstra, *p* < 0.001, N=4), and increases with UV dose in every absorber‐containing formulation (Jonckheere–Terpstra, *p* < 0.001, N=4). Each point represents one of the N = 4 gels per condition, prepared as four wells of the same plate in a single exposure. (h) Parametric study of thickness dependence on UV dose and GelMA concentration for fixed tartrazine (4.5 mg mL^−^^1^) and LAP (1 mg mL^−^^1^) concentrations. At each UV dose, higher GelMA content yielded thicker films (N≥3 gels per condition; 50 mg mL^−^^1^ did not form stable films at 3–8 mJ mm^−^^2^ and is therefore only shown at 13 and 21 mJ mm^−^^2^). (i) Programmable hydrogel mechanical properties measured by matrix nanoindentation. Each datapoint corresponds to the contact modulus extracted from each valid indentation, color coded according to the provenance from N=4 independent replicates per GelMA concentration (75, 100, and 150 mg mL^−^^1^). The thick black bars and boxes refer to the median and 25/75 distribution percentiles, respectively. The 3D renderings in (b) and (e) were obtained using NIS‐Elements software. Microscopy acquisition settings (illumination power, exposure time, binning, and gain) are reported in the Experimental Section.

**FIGURE 3:**
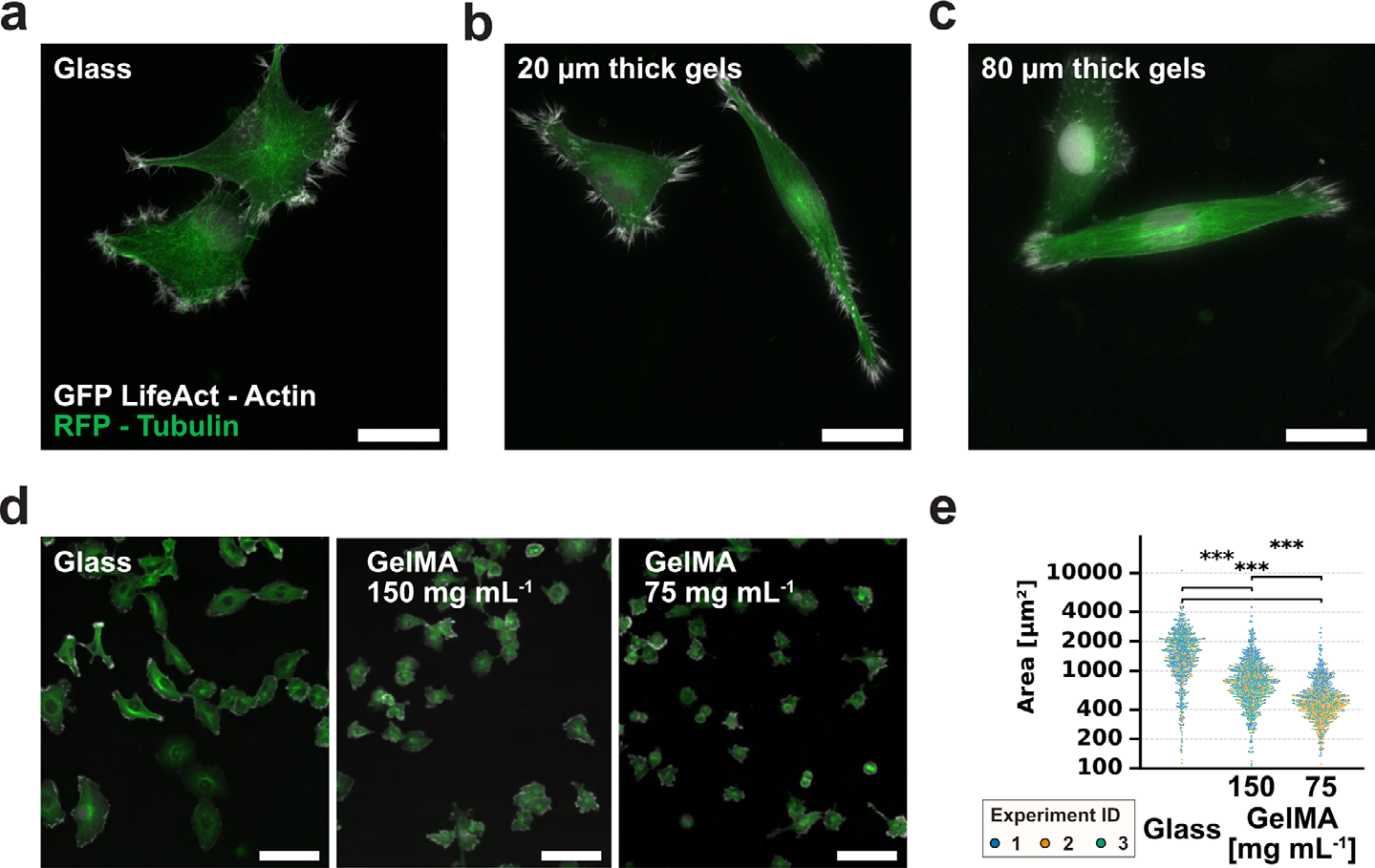
LigHTS hydrogels couple tissue‐like mechanics with microscopy‐optimized thickness, enabling high‐content phenotypic screens. (a–c) Fluorescence confocal imaging of HT1080 fibrosarcoma cells stably expressing LifeAct‐EGFP/Tubulin‐RFP on glass (panel a) and on thin soft (panels b and c) hydrogels at high (100x, silicone oil immersion objective) magnification. Since LigHTS films are ≤100 µm thick and sit on the coverslip, cells remain within the working distance of high‐numerical‐aperture objectives. Sub‐cellular features are crisply resolved on (a) glass and on LigHTS gels of (b) 20 µm and (c) 80 µm thickness, validating compatibility with high‐resolution, single‐cell read‐outs. Nuclear signal comes from the bleedthrough of the engineered FUCCIplex cell cycle sensor for cells in G0/G1 phase. Scale bars: 25 µm in (a), (b) and (c). (d) Fluorescence confocal imaging of HT1080 fibrosarcoma cells at low (20x dry objective) resolution. 75 and 150 mg mL^−^^1^ GelMA formulations correspond respectively to softer and stiffer hydrogel substrates (stiffness increasing with GelMA content; see Figure 2i). Scale bars: 100 µm. (e) Characterization of HT1080 cell area on different substrates. Actin and tubulin channels are used to extract cell area masks via the Cellpose‐SAM algorithm. The distributions indicate cell area modulation on substrates with different stiffness. Each point corresponds to a different cell (n > 150 per condition in each experiment), each color to a different experiment (N=3). Significance bars and stars between groups represent statistical significance for Holm‐corrected exact permutation test (*p*<0.001). Microscopy acquisition settings (illumination power, exposure time, binning, and gain) are reported in the Experimental Section.

**FIGURE 4:**
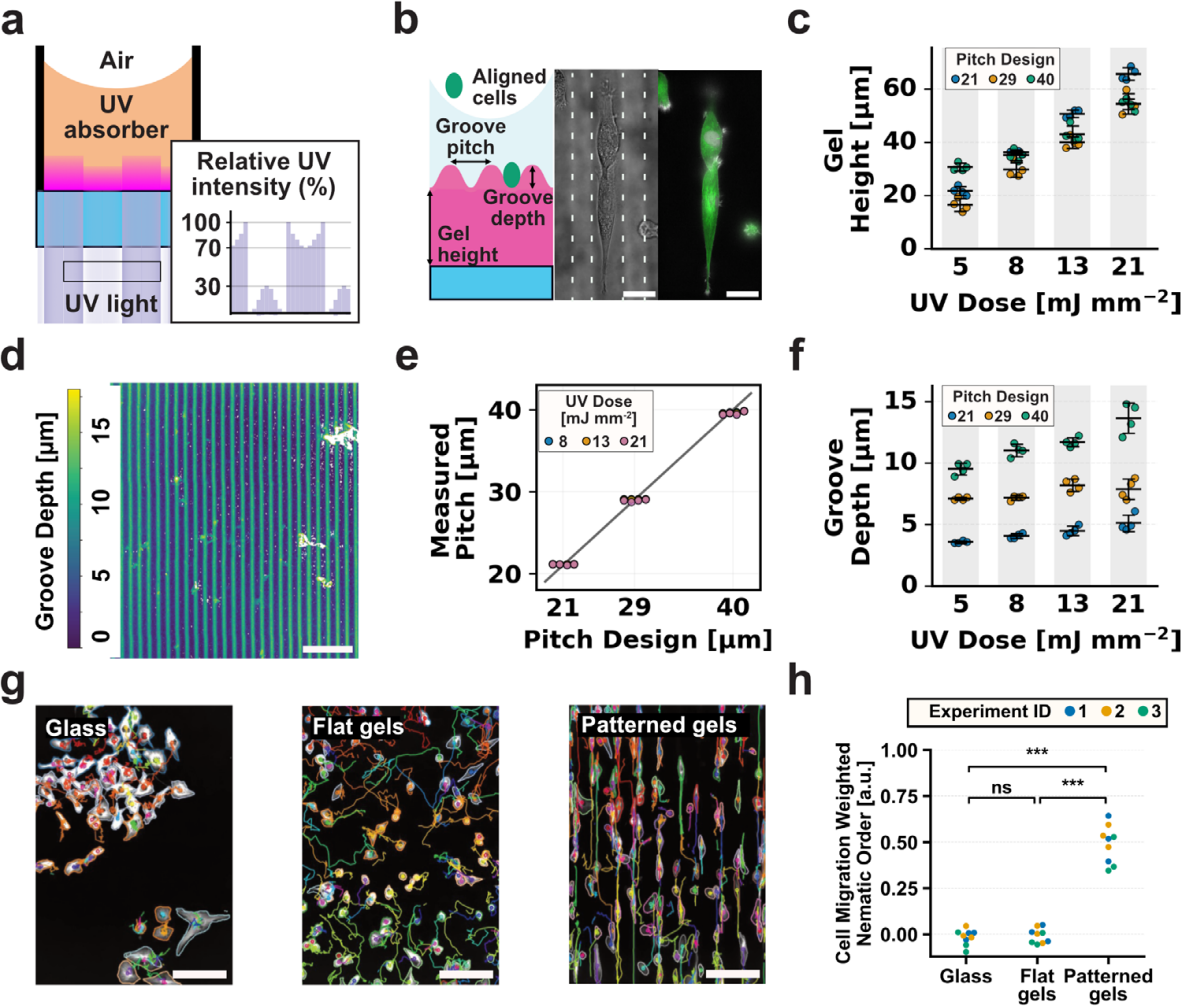
LigHTS fabrication method allows for surface photostructuring of hydrogels with single exposure, enabling contact guidance of cells. (a) Gradient light intensity profile translates into surface photostructuring via a single UV exposure. (b) Schematic illustration of the critical parameters in the grooved hydrogels, resulting in HT1080 cell polarization while maintaining high‐resolution compatibility. Green and gray represent tubulin and actin. Scale bar: 25 µm. (c) Mean hydrogel thickness of surface‐photostructured hydrogels with different UV doses and pitches. Gel thickness is determined by the local UV dose, which is pitch‐design dependent (N = 4 per UV dose per pitch design). (d) Heatmap reconstruction of photostructured hydrogel surface with groove depth quantification. Scale bar: 200 µm. (e) Measured pitch is highly conformal to the design across different spacings and different UV doses (N = 4 per UV dose per pitch design). (f) Quantification of groove depth as a function of UV dose for different pitch designs. Groove pitch is the dominant factor over UV dose in determining the groove depth (N = 4 per UV dose per pitch design). (g) HT1080 fibrosarcoma cell line migration experiment over a 12 h span. Compared to glass and isotropic gels, grooved hydrogels direct cell migration primarily along the main axis of the grooves. Colored lines are individual cell trajectories reconstructed by Cellpose segmentation and LapTrack linking [58, 59]. Color identifies track identity only and does not encode a quantitative variable. Scale bars: 50 µm. (h) Migration trajectory alignment referenced to the gel groove axis. Each point represents the Weighted Nematic Order (WNO) parameter averaged per field of view (FOV) weighted on the displacement of the individual cell migration tracks. WNO values run from +1 for displacement parallel to the grooves to −1 for displacement across them (N = 3 experiments, n = 3 replicate FOVs per condition, Figure S9b and Note S10). Photostructured grooves contact‐guide cells to migrate preferentially along the groove axis with statistical significance (exact blocked permutation stratified by experiment, Holm‐adjusted *p* < 0.001, N = 3 experiments with n = 3 FOVs each) relative to isotropic substrates. Microscopy acquisition settings (illumination power, exposure time, binning, and gain) are reported in the Experimental Section.

**FIGURE 5:**
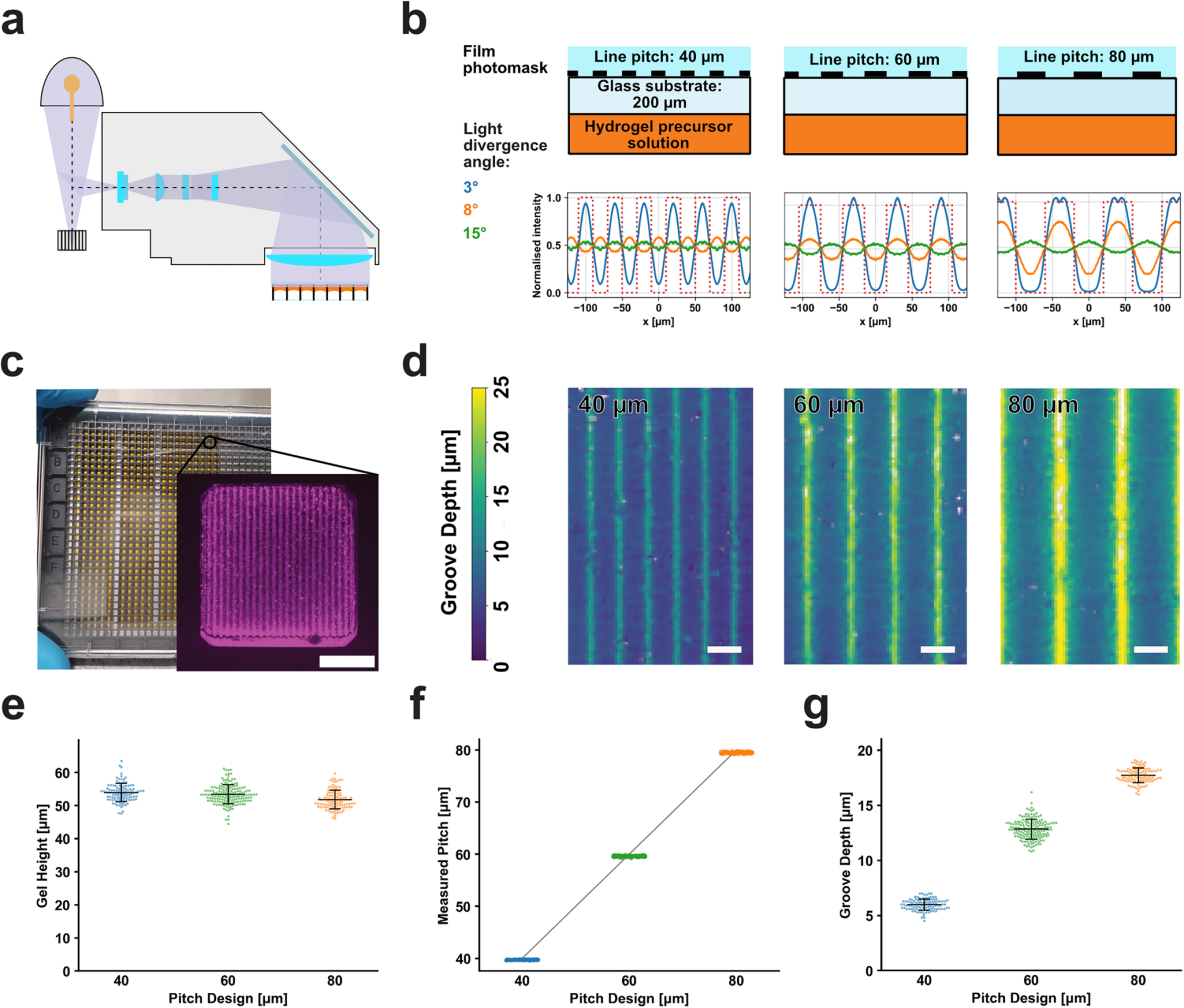
Ultra‐high‐throughput parallel fabrication of surface photostructured hydrogels. (a) Schematic of the collimated UV‐light system used for parallel whole‐plate fabrication through a film photomask. (b) Simulated normalized irradiance profiles at the gel plane for masks with different pitches, illustrating how diffraction and source divergence smooth the binary mask into a near‐sinusoidal modulation. (c) Proof of concept of parallel hydrogel photostructuring in standard 1536‐well plate format, with inset highlighting patterned grooves. Scale bar: 500 µm. (d) Heightmap reconstructions of photostructured hydrogel surfaces through a photomask with different pitches illustrating groove depth quantification. Scale bar: 50 µm. (e) Mean hydrogel thickness after 16 minutes of UV exposure. The graph shows good uniformity within each design, with slightly decreasing thickness values at increasing pitch spacing (N>100 wells per pitch). (f) Measured pitch is highly conformal to the design across different spacings (N>100 wells per pitch). (g) Groove depth is consistent between different technical replicates exposed through the same mask design, while pitch spacing causes a statistically significant increase in groove depth (Kruskal–Wallis test with Dunn's multiple comparisons, *p*<0.0001, N>100 wells per pitch). Microscopy acquisition settings (illumination power, exposure time, binning, and gain) are reported in the Experimental Section.

**FIGURE 6:**
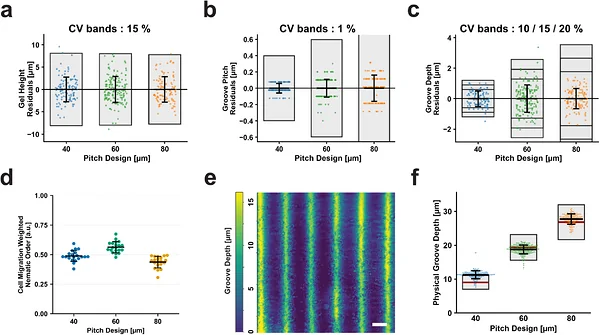
Reproducibility of LigHTS parallel photostructuring and validation by Holographic Phase Imaging. (a–c) Within‐plate reproducibility of LigHTS‐produced hydrogels (N>100 wells per pitch). Points are per‐well residuals with respect to the group mean and the gray bands are fixed coefficient‐of‐variation references drawn at the percentages printed above each panel. The measured within‐plate coefficient of variation is under 15% for gel thickness (panel a), under 1% for pitch design compliance (panel b), and under 20% for groove depth (panel c). (d) HT1080 cell migration along parallel photostructured gels with different pitches (N = 20 wells per pitch) in a 384‐well plate. Each point represents the Weighted Nematic Order (WNO) parameter averaged per field of view (FOV) weighted on the displacement of the individual cell migration tracks. The black bars denote mean ± SD; the panel is descriptive, and no inferential comparison between pitches is made. (e) Representative holographic phase map of a grooved gel, shown on an uncalibrated height scale. Scale bar: 50 µm. (f) Physical groove depth from holographic phase imaging, converted with the nanoindentation‐anchored factor C = 32 µm per native phase unit (n = 109, 159 and 107 wells at 40, 60 and 80 µm pitch). Each point represents the estimated physical gel thickness from one individual well, and the black bars denote mean ± SD. The dark red line is the confocal group mean converted with the axial factor ζ = 1.51, and the gray box is the expanded uncertainty (k = 2) of the HPI–confocal difference. The two modalities agree within this uncertainty at 60 and 80 µm pitch (−3.2% and +3.5%), whereas at 40 µm HPI exceeds confocal by 25% (Note S15 and Figure S11). Microscopy/holography acquisition settings (illumination power, exposure time, binning, and gain) are reported in the Experimental Section.

**FIGURE 7:**
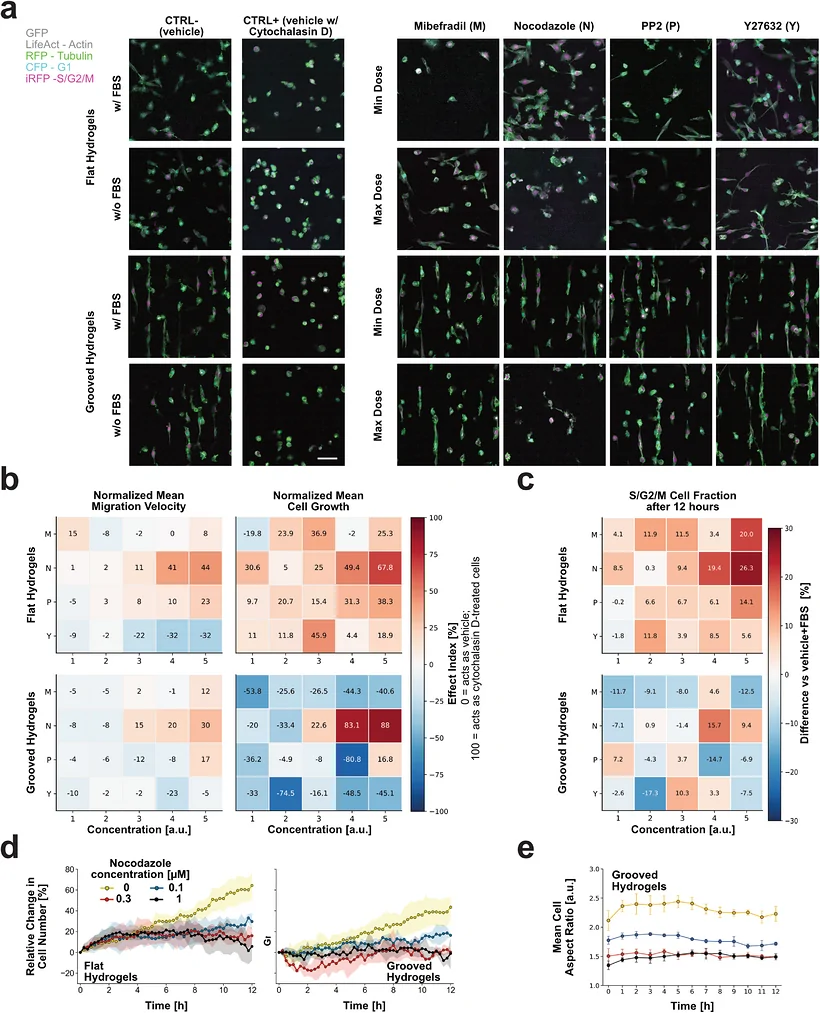
Proof‐of‐concept multiparametric pharmacological perturbation assay on flat and grooved LigHTS substrates. (a) Four‐channel confocal images at t = 720 min for all controls (left) and for all drugs (right) tested at minimum and maximum dose on flat (top) and grooved (bottom) substrates. The cell cycle S/G2/M and G1 reporters are shown in magenta and cyan, respectively, while structural markers are tubulin in green, and actin in gray. Scale bars: 100 µm. (b) Per‐condition mean cell speed averaged over all cells and frames (left) and cell‐number growth (right) for flat (top) and grooved (bottom) substrates. The values are expressed as signed control‐referenced effect indices in which 0 represents the same behavior as vehicle control and 100 the same drug response as cytochalasin D (positive control). Columns 1–5 correspond to the compound‐specific concentrations listed in Figure S12, and each matrix cell is the mean of three wells that received the same treatment. (c) Percentage‐point difference in the fraction of cells assigned to S/G2/M at 12 h relative to the matched vehicle‐with‐FBS condition, for flat (top) and grooved (bottom) substrates. (d) Relative change in mean cell number over time expressed as a percentage and normalized to the first frame for the vehicle with FBS and for nocodazole at 0.1 µm (N3), 0.3 µm (N4), and 1 µm (N5) conditions on flat (top) and grooved (bottom) substrates. (e) Change in cell aspect ratio (major over minor cell axis) over 12 h on grooved substrates for N3, N4, N5, and vehicle with FBS conditions. Data in d‐e are mean ± SD across three wells per condition from one plate‐scale experiment (N = 1).

## 2 Results

### 2.1 A Strong Optical Absorber in the Hydrogel Precursor Mix Enables the Creation of Thickness‐Tunable Hydrogels in Multiwell Plates

LigHTS spatially confines photopolymerization by introducing a strong UV absorber in a GelMA/LAP hydrogel precursor and illuminating the solution from the well glass bottom. Efficient photopolymerization requires radical generation through photoinitiator cleavage [47]. Conventional resins have photoinitiator species and concentrations optimized to generate radicals without strongly attenuating light through the precursor solution [48]. When the illumination dose exceeds the polymerization threshold for the liquid precursor, the bulk of the illuminated liquid volume crosslinks. As a result, focusing optics or layer‐by‐layer material confinement are the only options to constrain the gel thickness [39, 49], but are incompatible with parallel in‐plate patterning. For ease of prototyping, we started with a DMD and focusing optics (Figures 2, 3, 4) and switched to non‐focused collimated illumination after identifying the optimal conditions (Figures 5, 6, 7).

To establish the protocol, we dispensed precursor solutions into multiwell plates at volumes sufficient to fully wet the well glass substrate (typically ≥20 µL per 384‐well and ≥5 µL per 1536‐well). Then, we systematically mapped how UV dose (3–21 mJ mm^−^^2^), GelMA content (50–200 mg mL^−^^1^), and tartrazine concentration (0–9 mg mL^−^^1^) govern hydrogel thickness and flatness, illuminating the solutions at a room temperature of 23 ± 2°C. We used a commercial GelMA precursor (100 mg mL^−^^1^ GelMA with 2.5 mg mL^−^^1^ LAP in PBS; Figure 2a) to prepare an absorber‐free baseline (75 mg mL^−^^1^ GelMA solution with 10% v/v glycerol) and illuminated the precursor‐filled wells with UV light. Across all tested doses, the entire liquid column in the well crosslinked, producing millimeter‐thick hydrogels that are optically incompatible with high‐resolution cell imaging, since they introduce substantial scattering and optical aberrations. To characterize the optical gel height and surface topography by confocal microscopy, we coated the resulting gel surfaces after development with fluorescent beads. We observed that the hydrogel inherited the curvature of the air‐liquid meniscus of the hydrogel precursor solution, resulting in a highly curved surface (>200 µm center‐to‐wall height difference; Figure 2b,c and Video S1). Curved surfaces are suboptimal, since they produce spatial nonuniformity in the cell populations and complicate focusing necessary for optical characterization.

To avoid the described bulk photocrosslinking while working within well plates, we introduced tartrazine in the precursor solution as a non‐reactive UV absorber to compete with LAP for photon absorption, enabling dose‐controllable cure depth (Figure 2d). Single‐pass transmission measurements at 365 nm (Note S1 and Figure S1a) were performed in a 96‐well geometry with a defined 0.8‐mm optical path using different mixtures of buffer solution with and without tartrazine and LAP (Figure S1b), and the normalized transmission (Figure S1c) was used to calculate effective molar extinction coefficients of ε_365_ ≈ 1 × 10^4^ m ^−^^1^ cm^−^^1^ and 2.2 × 10^2^ m ^−^^1^ cm^−^^1^ for tartrazine and LAP, respectively. Therefore, at the tested concentrations, tartrazine dominates light absorption, effectively decoupling it from the presence of LAP, and shows no progressive photobleaching over exposure‐relevant timescales (Figure S1d). Beer‐Lambert attenuation steeply suppresses photon flux with depth and confines radical generation near the glass interface (Figure 1b). In this regime, oxygen presence creates an initial induction period before local sol–gel conversion proceeds [47], yielding an absorber‐defined crosslinking gradient [50]. Increasing the UV dose changes the depth at which the polymerization threshold sits and consequently the depth of precursor solution turning into a gel.

After the necessary exposure, the wells were washed using manual pipetting and vacuum aspiration to remove the whole tartrazine content (Figure S1e). We also used a tartrazine‐rich phantom solution and a standard liquid handling robot to verify that the tartrazine removal procedure could be automated (Note S2 and Figure S2a and Video S2). Specifically, we assessed that after six wash cycles the residual tartrazine dropped below the visible yellow‐tint concentration of 1 µg mL^−^^1^ tartrazine reference samples (Figure S2b and Note S6), indicating technical compatibility with machine operation and machine vision for the stop condition. After manually washing out soluble tartrazine from the gels (Figure S1e,f), we applied a post‐curing UV flood in 2‐(N‐morpholino)ethanesulfonic acid buffer (MES, 15 mm, pH 6) containing 1 mg mL^−^^1^ LAP to homogenize crosslinking through the film thickness (Figure S1g).

To generate hydrogels exceeding the limited DMD exposure field and covering the entire 384‐well glass substrate, we performed tiled illumination with sufficient overlap to obtain continuous gels (Figure S1h). We quantified optical gel thickness and flatness by coating the gel surface with fluorescent beads and reconstructing the top‐surface height from confocal z‐stacks using a custom Python pipeline (Note S3 and Figure S3a–c). Identification of the z position of the gel bottom and plate‐level tilt/warp normalization were achieved using gel‐free reference wells coated with fluorescent beads (Figure S3d–g), enabling absolute thickness comparisons across conditions.

Relative to absorber‐free gels, LigHTS produced thin, meniscus‐free films with an absolute ∼10‐fold reduction in thickness inhomogeneity within the well (Figure 2e,f and Video S3). At each UV dose tested, thickness decreased monotonically with tartrazine concentration (Jonckheere–Terpstra, *p* < 0.001, N = 4 wells per condition, Figure 2g). Within tartrazine‐containing formulations (2.25, 4.5, and 9 mg mL^−^^1^), thickness showed strong UV dose dependence (Jonckheere–Terpstra, *p* < 0.001, N = 4 wells per dose) and increased monotonically with UV dose (Holm‐adjusted permutation slope tests, *p* < 0.001, N = 4 wells per dose). In contrast, absorber‐free gels showed no significant increase in thickness with UV dose (Jonckheere–Terpstra, *p* = 0.99, N = 4 wells per dose). Across the tested range, 2.25, 4.5, and 9 mg mL^−^^1^ tartrazine reduced average thickness by ∼3‐, ∼15‐, and ∼35‐fold, respectively (N = 4). At the highest absorber/lowest dose, films became too thin to reliably quantify, whereas at low absorber/highest dose, films exceeded the z‐stack range (>180 µm). In the 10–100 µm window, thickness variation across an individual well remained <20% (single‐digit‐micron SD; Videos S1 and S3) within the DMD‐patterned field of view, enabling fine dose‐based thickness tuning. Balancing tunability against exposure time, 4.5 mg mL−1 tartrazine provided the most practical operating point (Figure 2g). The stitched illumination required by the focusing optics produces a visible tile‐like inhomogeneity of the gel surface. This artifact is intrinsic to focusing‐optics‐based photopatterning, and we avoid it by photopatterning tartrazine‐containing precursor solutions with collimated light (Figures 5, 6, 7).

### 2.2 LigHTS‐Patterned Hydrogels can be Stiffness‐Tuned by Adjusting GelMA Content and are Compatible With Long‐Term Storage

To tune mechanics via polymer content, we fixed tartrazine at 4.5 mg mL^−^^1^ and prepared GelMA formulations spanning 50–200 mg mL^−^^1^ (with LAP 1 mg mL^−^^1^ to expand the thickness tuning range under high irradiance). Across this matrix, film thickness increased with both UV dose and GelMA concentration (Figure 2h and Video S4; Jonckheere–Terpstra within each dose, *p* < 0.001, N ≥ 3 wells per condition), and the lowest GelMA formulation (50 mg mL^−^^1^) only formed measurable films at ≥13 mJ mm^−^^2^ (no stable films at 3–8 mJ mm^−^^2^). These results indicate that, for matched UV dose and tartrazine concentration, the hydrogel composition remains the primary factor governing polymerization dynamics and the resulting gel properties, in particular the absolute values of gel thickness and mechanical modulus.

To evaluate how the tartrazine‐mediated thickness control impacts the stiffness of LigHTS‐patterned gels, we performed nanoindentation characterization of the gels after UV flooding: the experiment confirmed that LigHTS preserves the expected polymer‐concentration‐dependent stiffness for GelMA hydrogels (Figure 2i). We measured the mechanical properties of hydrogels prepared by varying the GelMA content (75, 100, and 150 mg mL^−^^1^) in the precursor solution, finding the median values of the contact moduli increase from roughly 4 kPa at 75 mg mL^−^^1^ to 7 kPa at 100 mg mL^−^^1^ and 9 kPa at 150 mg mL^−^^1^ (Jonckheere–Terpstra, *p*<0.05, N = 4 independent gel replicates per concentration, Figure 2i). In terms of spatial homogeneity, the mechanically identified surfaces were globally planar, with 95% of detrended contact‐coordinate residuals falling within 2 µm from the median contact position (Note S4 and Figure S4). The within‐gel contact modulus distribution was in line with the typical spread values reported for similar characterization of ECM‐derived soft gels (Figure S5) [51, 52, 53], with contact‐point determination and finite sample thickness identified as major sources of uncertainty in soft‐hydrogel indentation [54, 55].

LigHTS‐patterned hydrogels were structurally and mechanically stable over one month of hydrated cold storage (4°C in PBS, Note S5 and Figure S6a,b). Most of the batch‐to‐batch variability related to the resulting value of gel thickness and contact modulus appears to be concentrated across the polymerization threshold regime determined by a combination of lot differences and critical process variables such as precursor temperature for thermosensitive formulations (Figure S6c,d). Industrial applications of this technique would therefore require a lot‐to‐lot calibration and strict control over the critical process parameters to ensure maximum reproducibility across plates.

### 2.3 LigHTS‐Patterned Hydrogels as Biomimetic Substrates for Human Cell Culture and Compatible With High‐Resolution Imaging

We next assessed whether fabricated hydrogels supported human cell culture as a biomimetic substrate. First, to ensure no toxic concentration of tartrazine leached from the fabricated gels, we indirectly measured the presence of tartrazine residual by characterizing the optical absorption of the PBS solution soaking the gels overnight after the complete fabrication procedure (Note S6). We found that the tartrazine levels were below 0.25 µg mL^−^^1^ after 12 h of incubation in fresh PBS (Figure S7a,b), roughly 40 times lower than the reported cytotoxic values of tartrazine toward mammalian cells [56, 57].

We then tested and verified that the produced hydrogels could support cell adhesion and growth and induce stiffness‐induced phenotype modulation of HT1080 cells expressing actin/tubulin fluorescent reporters [45, 46]. Benchmarking against the gold standard of cells on glass (Figure 3a), LigHTS films still supported high‐quality confocal imaging at 100x (silicone‐oil immersion) across 20–80 µm thicknesses with minimal background or aberration (Figure 3b,c). At screening‐relevant magnification (20x), we quantified substrate‐dependent spreading by segmenting actin/tubulin images and measuring projected cell area (Figure 3d,e and Note S7). HT1080 cells exhibited reduced area on 150 and 75 mg mL^−^^1^ GelMA films relative to glass, with the smallest areas on 75 mg mL^−^^1^ films (Holm‐adjusted exact blocked permutation on field‐of‐view median, *p* < 0.001, N = 3 independent experiments with 3 field‐of‐view (FOV) technical replicates per condition; Figure 3e).

As an additional epithelial model, HaCaT human keratinocytes genetically engineered in prior work were cultured on 75 mg mL^−^^1^ GelMA films and glass controls [45]. Differently from HT1080, HaCaT cells do not show significant migratory behavior and instead assemble into colonies, which have been reported to probe the mechanical properties of the substrate reaching much deeper than individual cells [20]. Cells adhered and formed colonies on both substrates, with qualitatively reduced single‐cell areas for colonies on the ∼80‐µm‐thick GelMA gels (Note S8 and Figure S7c,d), indicating that the hydrogel thickness was sufficient to screen the glass substrate stiffness.

### 2.4 Structured Illumination Provides a Scalable Approach to Controlling Gel Topography and Guiding Cell Behavior, and is Applicable to Other Hydrogel Chemistries

To add instructive anisotropy in a contact‐free manner, we patterned grooves directly during photopolymerization using grayscale structured illumination (Figure 4a). We targeted aligned microgrooves with peak‐to‐peak pitches of ∼21, ∼29, and ∼40 µm, dimensions matched to single‐cell length scales and compatible with DMD pixel quantization (Figure 4b and Figure S8a,b). Illumination patterns were designed with sharp dose transitions to maximize relief amplitude despite radical diffusion and scattering (Figure 4a). For groove mapping, we used 150 mg mL^−^^1^ GelMA with 4.5 mg mL^−^^1^ tartrazine and 1 mg mL^−^^1^ LAP, which supports efficient thickness control in the 10–100 µm regime. Patterned exposures used nominal UV doses of 5–21 mJ mm^−^^2^, but the grayscale modulation reduced the large‐area effective dose by ∼2x (Figure 4c): accordingly, grooved films were roughly half as thick as their uniformly exposed counterparts at the same nominal dose (Figure 2h).

Using a dedicated Python surface‐reconstruction pipeline (Figure 4d and Note S9 and Figure S8c–g), we quantified groove geometry within single DMD fields to avoid stitching steps at tile boundaries. Measured pitch closely matched the design across all conditions (overall deviation lower than the lateral 20x sampling resolution used here; Figure 4e). Groove depth spanned approximately 3.5–15 µm and increased strongly with pitch at every tested dose (Holm‐adjusted permutation slope tests, *p* < 0.001 for all doses, N = 4 gels per dose and pitch; Figure 4f).

Finally, we evaluated whether these microgrooves transmit functional guidance cues by tracking HT1080 migration over 12 h on fibronectin‐coated glass, flat gels, and grooved gels (40 µm pitch; Figure 4g and Figure S9a and Video S5). We summarized trajectory alignment using a displacement‐weighted nematic order parameter (WNO) referenced to the groove axis, which runs from +1 for persistent motion parallel to the grooves to ‐1 for motion across them (Figure S9b and Note S10). Cells on grooved gels migrated more aligned with the groove axis than on glass or flat gels (exact blocked permutation with Holm adjustment: *p* < 0.001 for both contrasts, N = 3 experiments with n = 3 FOVs per condition; Figure 4h and Figure S9c–e).

To further validate the biological value of grooved hydrogels, we used human induced pluripotent stem cell (hiPSC)‐derived cardiomyocytes (hiPSC‐CMs) as a proof of concept of compatibility with a mechanosensitive stem‐cell‐derived lineage and of the ability to induce tissue‐level organization (Note S11 and Figure S8h and Video S6). We differentiated hiPSCs into cardiomyocytes as previously described [45], and seeded them at high density on 40‐µm‐pitch grooved hydrogels prepared using 150 mg mL^−^^1^ GelMA precursor solution. The cells adhered, formed a partially aligned monolayer following the groove direction, and retained spontaneous contractile activity (Video S6), confirming that the patterned substrate supports both the structural organization and the contractile function of this lineage.

The strategy is in principle compatible with other radical‐polymerized formulations. To test transferability beyond GelMA, we performed proof‐of‐concept experiments with methacrylated hyaluronic acid (HAMA) and poly(ethylene glycol) diacrylate (PEGDA, Note S12 and Figure S10). Low‐molecular‐weight HAMA (10–20 kDa; 75 mg mL^−^^1^) reproduced the UV‐dose‐to‐thickness dependence measured for GelMA, with height of 40‐µm‐pitch grooved gels rising from 30.2 to 44.4 µm between 8 and 21 mJ mm^−^^2^ (Figure S10a–c). PEGDA of 575 Da (7.5% w/w) reproduced the groove‐pitch‐to‐depth dependence at a fixed dose, with depth rising from 8.4 to 18.6 µm between 21 and 40 µm groove pitch design (Figure S10d–f). Both trends are monotonic (Jonckheere‐Terpstra, *p* < 0.0001; five wells per condition on one plate). The comparison of the absolute values of gel thickness and groove depth with GelMA hydrogels obtained with matched UV dose and tartrazine content confirms that those properties remain material‐specific and depend on the processing conditions, as previously discussed.

### 2.5 Collimated Light Unlocks Highly Reproducible, Plate‐Wide Fabrication

Having established dose/formulation control and groove performance in the prototype DMD configuration, we replaced serial, focused crosslinking with massively parallel plate‐wide exposure using highly collimated UV light from a mask aligner, illuminating inverted plates to expose all wells simultaneously (Figure 5a). The inclusion of 10% v/v glycerol increased precursor viscosity and prevented leakage during inverted exposure. To generate grooves at plate scale, we interposed whole‐plate film photomasks with parallel opaque lines (40, 60, and 80 µm pitch). Because the mask‐to‐gel distance is set by the glass thickness (∼200 µm), diffraction converts the binary mask into a smooth, near‐sinusoidal modulation at the hydrogel plane, with optics‐only simulations capturing this behavior and indicating tolerance to light source divergence (Note S13 and Figure 5b). Using a single 16‐min exposure at ∼3.5 mW cm^−^^2^, we patterned a complete 1536‐well plate in parallel (Figure 5c). Confocal metrology confirmed continuous groove fields across wells (Figure 5d), with bulk film thickness between ∼50 and ∼60 µm and good within‐design uniformity (Figure 5e). Groove pitch remained highly conformal to the mask design across >100 wells per condition (Figure 5f), and groove depth increased significantly with pitch, from ∼6 µm at 40 µm pitch to ∼18 µm at 80 µm pitch (Kruskal–Wallis test with Dunn's multiple comparison, *p*<0.0001, N > 100 wells per pitch; Figure 5g).

### 2.6 Plate‐Scale Metrology

To benchmark process capability against industrial screening needs, we quantified within‐plate coefficients of variation (CV) across more than 100 wells per condition: thickness CV was <15% (Figure 6a) and pitch CV approached ∼1% (Figure 6b), while depth CV was <20% (Figure 6c), approaching the measurement limits. This confirms that the LigHTS approach is highly conformal within the plate, allowing the parallel production of screening‐suitable microgrooved hydrogels. We then repeated the migration assay on mask‐aligner‐fabricated plates and observed contact guidance consistent with the DMD‐patterned plates (Figure 6d and Video S7).

Finally, we implemented holographic phase imaging (HPI) as a rapid, label‐free QC modality compatible with industrial throughput. Compared with confocal z‐stacking (0.1–1 s exposures), holographic imaging requires 1‐10 ms exposures and reads out groove topography from a single phase acquisition (Note S14 and Figure 6e). To find the physical value of groove depth, we performed a simple cross‐calibration experiment by first reconstructing a single (280 x 280) µm^2^ FOV from a grooved hydrogel (40 µm design pitch, 75 mg mL^−^^1^ GelMA) via nanoindentation (Figure S11a) and then acquired groove depth by confocal (Figure S11b) and HPI (Figure S11c) to compare and calibrate the extracted values (Note S15 and Figure S11d). The optical groove depth extracted by the confocal pipeline can be converted to a physical value matching the depth characterized via nanoindentation using an axial factor ζ = 1.5 ± 0.1 (Figure S11e). This value agrees with the wave‐optical prediction for a dry NA 0.75 objective focused into a medium of refractive index ≈1.35 (ζ = 1.47) [60], and lies within the paraxial‐to‐marginal envelope of 1.35–1.70 [61]. Similarly, the phase delay information from HPI can be converted to a physical groove depth using a phase‐to‐relief factor C = 32 ± 2 µm per native phase unit (Figure S11f). Applied to the plate dataset, the calibrated confocal and HPI reconstructions substantially agree on the estimated physical groove depth at 60 and 80 µm pitch, while we observe a larger difference at 40 µm, which we attribute to the groove depth approaching the uncertainty of the confocal measurement pipeline (Figure 6f). These are first‐approximation conversion factors obtained from one calibration specimen, while a broader calibration campaign across pitches, formulations and objectives would be needed to generalize them. HPI was also used to track cell migration on the grooved field without fluorescent labels (Note S16 and Video S8). Together, these results establish a practical fabrication‐metrology loop for biomimetic, imaging‐ready substrates at UHTS scale.

### 2.7 LigHTS Substrate Compatibility With Perturbation Assay in High‐Throughput Formats

To test whether LigHTS substrates support plate‐scale compound perturbation assay, we performed a single‐plate proof‐of‐concept pharmacological assay using HT1080 cells on flat and 60‐µm‐pitch grooved LigHTS gels (Figure 7). We selected nocodazole, PP2, mibefradil, and Y‐27632 as mechanistically distinct perturbations of pathways implicated in HT1080 adhesion and motility, spanning microtubule‐dependent adhesion turnover, Src‐family signaling, Ca^2^^+^ dynamics, and ROCK‐dependent actomyosin regulation [62, 63, 64, 65]. Each compound was tested across five concentrations, with three replicate wells per concentration and hydrogel geometry (Figure S12). Four fluorescence channels were acquired every 15 min for 12 h, enabling stratified analysis of the cell response considering morphology, migration, total cell number, and FUCCI cell‐cycle state (Figure 7a and Video S9) [45, 46]. Nocodazole produced the clearest concentration‐associated phenotype modulations, while the other compounds did not produce similarly coherent responses across endpoints under these conditions (Figure 7b,c). Specifically, cells treated with nocodazole at 0.3‐1 µm concentration displayed changes in cell elongation, migration, cell‐number dynamics, and S/G2/M progression on both hydrogel geometries (Figure 7d,e). The plate carried its own control for the topography: vehicle wells gave a weighted nematic order of +0.59 on grooved substrates against ∼0 on flat, reproducing the contact guidance of Figure 4h. While this single plate‐scale experiment (N = 1, n = 3 wells per condition) cannot be used for inferential drug‐by‐topography comparisons or comparative pharmacological ranking, this proof of concept establishes compatibility of LigHTS with multiplexed compound perturbation and longitudinal multiparametric phenotyping. Future work will be directed toward a full validation of the imaging‐based screening pipeline on these substrates, targeting differential hit discovery and drug‐effect‐by‐substrate studies.

## 3 Discussion

LigHTS converts a long‐standing bottleneck in plate‐based mechanobiology into a controllable materials‐processing problem, providing tunable thickness, stiffness, and surface topography inside standard 384‐ and 1536‐well plates in a single exposure. By adding tartrazine as a strong, food‐grade UV absorber that dominates photon transport at 365 nm, we decouple cure depth from focusing optics and make thickness a predictable function of dose and formulation (Figure 2d,g). This chemistry shift suppresses meniscus‐driven thickness gradients (Figure 2b,c) and enables thin, microscope‐compatible GelMA films (10–200 µm) with programmable soft‐tissue mechanics (Figure 2h,i) formed directly in well plates and compatible with high‐NA confocal imaging (Figure 3b,c). Critically, the same approach scales from serial DMD prototyping to plate‐wide manufacture: collimated illumination combined with inexpensive whole‐plate film photomasks generates microgrooves in standard 1536‐well formats in a single exposure (16 min at ∼3.5 mW cm^−^^2^; Figure 5c–g), replacing stitched serial exposure with massively parallel patterning. Because tartrazine sets the axial light‐intensity gradient and not the crosslinking chemistry, the same dose‐to‐thickness control carries over to any radical‐polymerized precursor in which the added absorber dominates attenuation at the exposure wavelength, as the HAMA and PEGDA proof‐of‐concepts demonstrate (Figure S10 and Note S12). Together with scalable metrology, such as confocal surface profiling and rapid holographic phase imaging for millisecond readout (Figure 6e,f), both calibrated against nanoindentation, LigHTS provides an end‐to‐end route from substrate manufacture to phenotype readout with screening‐relevant within‐plate reproducibility (thickness CV <15%, groove pitch CV ∼1%, groove depth CV <20%; Figure 6a–c). We anticipate immediate value for three communities: in vitro tissue engineering and mechanobiology, imaging‐based phenotyping and AI, and industrial screening/QC. In fact, they share the common bottleneck of introducing biomimetic mechanics and anisotropy into plate‐native workflows without sacrificing scale, optics, or reproducibility [8, 9, 10, 11, 16, 17, 18, 19, 20].

A persistent barrier to mechanobiology at scale is that substrates are either scalable but mechanically unrepresentative (rigid plates) or biomimetic but difficult to standardize (organoids and organs‐on‐chips), leaving most early discovery screens blind to mechanical and architectural context [8, 9, 10]. LigHTS reduces this trade‐off by delivering two high‐leverage ECM variables, compliance and anisotropy, directly in 384/1536‐well plates with minimal assembly burden. In practice, the absorber‐guided working curve collapses the uncontrolled, millimetric, meniscus‐shaped gels produced by conventional well curing (Figure 2a–c) into 10–200 µm films whose height is directly controlled by UV dose exposure, with an order‐of‐magnitude reduction in thickness inhomogeneity (Figure 2e,f). The resulting substrates span median contact moduli of ≈4–9 kPa between 75 and 150 mg mL^−^^1^ GelMA (Figure 2i) and microgrooves with single‐cell‐scale pitches (40–80 µm) and relief amplitudes (≈6–18 µm) (Figure 5f,g), enabling scalable studies of how stiffness and alignment regulate spreading, polarization, and migration (Figure 3d,e and Figure 6d). The modulation of the cell area at single‐cell (HT1080) and colony (HaCaT) level (Figure 3e and Figure S7c,d) indicates that LigHTS hydrogel thickness can satisfy the thickness requirements for mechanical isolation needed from single‐cell to tissue levels [18, 19, 20]. Beyond HT1080 and based on the proof‐of‐concept compatibility with HaCaT and hiPSC‐CM, the immediate opportunity is to port alignment‐ and mechanics‐sensitive systems (e.g., myocytes, neurons, stromal cells) into UHTS‐compatible screening designs without abandoning the plate‐native automation ecosystem [16, 17, 18, 19, 20].

Current imaging‐based UHTS workflows can generate large, clean image datasets, but their biological mimicry is often weak: culturing on rigid, flat substrates can suppress phenotypic diversity or introduce substrate‐specific artifacts that confound generalization [12, 13]. LigHTS addresses this gap directly: thin films remain compatible with established pipelines and high‐NA microscopy (confocal imaging at 100x; Figure 3b,c) while introducing controlled stiffness and topography that elicit interpretable phenotypes (stiffness‐dependent spreading and groove‐dependent contact guidance; Figure 3d,e and Figures 6d and 7). For translation, the decisive requirement is that biomimetic substrates can be produced and qualified like a manufactured product, that is, plate‐native, high‐throughput, and traceable. LigHTS was engineered around that requirement. Plate‐wide collimated exposure eliminates stitching artifacts and throughput limits intrinsic to focused serial patterning, and whole‐plate film masks provide a low‐cost path to anisotropic architectures (Figure 5a–c). At scale, we achieve well‐level reproducibility consistent with screening tolerance bands, such as thickness CV <15%, pitch CV ∼1%, and depth CV <20% across hundreds of wells (Figure 6a–c). Importantly, we propose a label‐free QC that is scalable to industrial standards. Holographic phase imaging can verify groove fields and support low‐phototoxic tracking with 1–10 ms exposures (Figure 6e and Video S8), enabling large datasets where substrate geometry is verified for each well. The LigHTS fabrication protocol was also shown to be compatible with an automated liquid‐handling robot (Figure S2), and the gels were stable under long‐term cold storage (Figure S6). Our results establish the biomimetic features of the platform (tissue‐like compliance, mechanical isolation from the rigid well bottom, and instructive anisotropy), which are known to modulate cell phenotype [18, 19, 20, 24]. Because stiffness‐driven processes converge with biochemical signals on common pathways [13], substrate mechanics can also shape drug responses, and compounds with stiffness‐dependent effects have been identified in a multiwell screen [31]. Topography likewise modulates functional endpoints used in drug testing as previously shown for aligned cardiomyocyte tissues [26]. Organs‐on‐chips followed a similar path, with biomimicry established first and predictive value subsequently tested against compounds of known action or toxicity [7, 15]. In the single‐plate proof‐of‐concept assay (N = 1; Figure 7), nocodazole elicited the clearest concentration‐associated response in HT1080 cells on both flat and grooved gels. Because the assay was run on a single plate, however, it can neither establish substrate‐dependent drug effects nor show whether biomimetic substrates yield different or more relevant hits than rigid, flat ones. Future work will assess the predictive value of LigHTS plates in replicated, multi‐plate screens that directly compare hit calls on biomimetic and rigid substrates [14], alongside the standard industrial milestone of demonstrating between‐plate process capability (C_pk_ across lots).

In summary, LigHTS offers a practical bridge between biomimetic materials and industrial screening: thin, soft, microstructured substrates fabricated directly in standard 384/1536‐well plates with plate‐wide throughput and measured QC, including a scalable modality for testing geometry compliance. While the current implementation focuses on GelMA and a limited set of cell responses, the underlying principles are broadly applicable in future extensions to new chemistries and biological models. In the near term, this enables mechanobiology‐aware screenings without changing current imaging infrastructure; in the longer term, it can accelerate AI‐enabled microphysiological modeling by supplying large, standardized, biomimetically relevant datasets with substrate traceability [9, 10, 11].

## 4 Experimental Section

### 4.1 Well Plate Cleaning and Silanization

To promote GelMA adhesion, glass‐bottom 384‐well plates (P384‐1.5H‐N, Cellvis) or 1536‐well plates (M4562, Sigma–Aldrich) were cleaned and silanized before casting. Wells were incubated 1 h at 37°C with 2% v/v Hellmanex III (Z805939, Sigma–Aldrich) in sterile‐filtered DI water, rinsed 3x with 37°C DI water, and dried. The glass bottom of the wells was then silanized with 3‐(trimethoxysilyl)propyl methacrylate (TMSPM, 440159, Sigma–Aldrich): 0.4% v/v TMSPM in aqueous solution at pH 3.5 (glacial acetic acid, 33209‐M, Sigma–Aldrich), stirred until clear; wells were incubated 1 h at RT, rinsed 2x with 37°C DI water and once with ethanol, then dried. Plates were used immediately or sealed (Parafilm, 11772644, Fisher Scientific) and stored RT ≤ 1 week.

### 4.2 Preparation of the Hydrogel Precursor Solutions

For testing the effect of different tartrazine concentration on gel thickness, a stock of 100 mg mL^−^^1^ GelMA with 2.5 mg mL^−^^1^ LAP in PBS (D16110025360, Cellink) was mixed with tartrazine (T0388, Sigma–Aldrich), LAP (900889, Sigma–Aldrich), and glycerol (327255000, Thermo Fisher Scientific) in PBS (pH 7.4; 10010015, Gibco) to obtain 75 mg mL^−^^1^ GelMA, 2.5 mg mL^−^^1^ LAP, 10% v/v glycerol, and tartrazine 0–9 mg mL^−^^1^.

For testing the effect of different concentrations of GelMA on scaffold thickness and stiffness, groove generation, and cell response, lyophilized GelMA (Type A, Bloom 300; degree of methacrylation > 75%) from Advanced Biomatrix (5208) or Sigma–Aldrich (938408) was dissolved in PBS. LAP and tartrazine were pre‐dissolved in PBS to target 1–5 mg mL^−^^1^ (LAP) and 2.25–9 mg mL^−^^1^ (tartrazine), stirred until clear, sterile‐filtered (0.22‐µm pore PVDF membranes), and used immediately or stored 4°C, protected from light, ≤ 4 days. To dissolve GelMA efficiently, the LAP/tartrazine solution was heated to 45–60°C. To increase viscosity and decrease oxygen solubility, glycerol was added to reach 10% v/v of the hydrogel precursor solution. Then GelMA was added to the solution to reach a concentration in the range 50–200 mg mL^−^^1^ and stirred at 1000 rpm ≥ 20 min until fully dissolved. Specifically, for testing contact guidance, lyophilized GelMA was dissolved in PBS containing 1 mg mL^−^^1^ LAP and 4.5 mg mL^−^^1^ tartrazine to reach the concentration of 150 mg mL^−^^1^.

### 4.3 Casting of the Hydrogel Precursor Solution in Multiwell Plates

To avoid physical GelMA gelation during casting, the receiving well plates and pipette tips were heated up to keep the viscosity of GelMA low during casting. Silanized plates were equilibrated 20 min at 37°C. For 384‐well plates, 100 µL tips pre‐warmed in 45–60°C DI water transferred ≥ 20 µL precursor per square well (≈ 11 mm^2^ area). For 1536‐well plates, 12.5 µL tips transferred ≥ 5 µL (≈ 2.3 mm^2^ area). Plates were centrifuged 5 min at 1500 x g (SL16R, Thermo Fisher Scientific) to remove bubbles and wet the glass. Plates were cooled 5 min at 4–8°C, then exposed immediately or within 2 h (protected from light).

### 4.4 DMD Photopatterning, Development, and Post‐Exposure UV Flood for Flat Gels

A PRIMO maskless DMD system (Alvéole) on a Nikon ECLIPSE Ti2, controlled by Leonardo v2.4 (Alvéole), illuminated through a 4x air objective (CFI Plan Fluor 4x, NA 0.13, MRH00045). The single‐field area was ≈ 2.5 mm x 1.5 mm. Irradiance at 365 nm at full‐field 100% was ≈ 0.8 mW mm^−^^2^ (estimated from 3 mW measured with Argo‐POWER HM Slide V2, Argolight). Uniform exposures used Leonardo dose control to reach 3–21 mJ mm^−^^2^. Development used warm (37°C) 2‐(N‐morpholino)ethanesulfonic acid buffer (MES, 15 mm, pH 6.0 ± 0.1; 76039, Sigma–Aldrich), prepared from 0.5 m stock in DI water with NaOH (S2770) pH correction. Gels were incubated in MES three times with 30 min exchanges at 37°C. After the third rinse, gels were left overnight at 37°C, then rinsed once more. For the post‐exposure flood, LAP (1 mg mL^−^^1^) in MES was added (20 µL in 384‐well; 5 µL in 1536‐well) and plates were illuminated for 15 min using a commercial dual‐wavelength (365/405 nm) UV LED curing light delivering ≥10 J cm^−^^2^ at the plate plane (13 ± 3 mW cm^−^^2^ across 12 mapped positions; calibrated silicon photodiode referenced to 365 nm, 0.64 cm^2^ aperture; Argo‐POWER HM V2, Argolight). Gels were rinsed 3x with PBS and stored in PBS containing 0.2% v/v Primocin (ant‐pm‐05, InvivoGen) ≥ 24 h before cell seeding.

### 4.5 Gel Surface Functionalization Using Fluorescent Beads

To label the gel‐liquid interface for metrology, surfaces were coated at RT using MES throughout. Samples were equilibrated once in MES; poly‐L‐lysine (PLL, P4707, Sigma–Aldrich) 0.0025% w/v in MES (from 0.01% w/v stock) was applied 30 min, then rinsed once with MES (2 min). Carboxylated polystyrene beads (FluoSpheres, 0.2 µm, 660/680 nm, F8807, Invitrogen; 2% solids) were freshly diluted 1:100 in MES, dispensed, and plates were centrifuged 5 min at 500 x g to accelerate sedimentation, followed by 10 min static incubation. After gentle aspiration and one MES rinse, a PLL “cap” (0.0025% w/v in MES) was applied 15 min and rinsed twice with MES. PBS was then added to half‐volume to equilibrate osmolarity; 15 min incubation at 37°C; finally, wells were fully exchanged to PBS and equilibrated ≥ 1 h at 37°C before imaging.

### 4.6 Gel Profile Characterization by Confocal Fluorescence Microscopy

Spinning‐disk confocal (Crest V3 x‐Light on Nikon) with CELESTA Light Engine (TSx5030FV, Lumencor) and a Photometrics Kinetix sCMOS camera acquired 4x4‐binned z‐stacks of the bead layer (red excitation, 638 nm, or 655 nm where indicated for 20x acquisitions, 50 ms exposure). Unless otherwise stated, all fluorescence images were acquired in confocal mode in the camera's 12‐bit mode. Exposure time, binning, pixel size, z‐step, and frame interval of every acquisition are recorded in the metadata of the raw ND2 files (BioImage Archive, S‐BIAD4164). Excitation powers, which are not recorded in the metadata, were measured at the specimen plane with an Argo‐POWER HM power meter (Argolight) at 100% source intensity: 6.8 mW (638 nm) with the 4x/0.13 NA objective; 4.5 mW (446 nm), 5.8 mW (477 nm), 4.9 mW (546 nm), and 5.5 mW (638 nm) with the 20x/0.75 NA objective; and 0.78 mW (477 nm) and 0.70 mW (546 nm) with the 100x/1.35 NA objective. Objectives and z‐sampling were chosen by expected thickness: 4x air (CFI Plan Fluor 4x, NA 0.13) for thick gels (> 0.2 mm) and quick screening, Δz = 30 µm; 10x air (CFI Plan Apo 10x, NA 0.30, WD 1.6 mm, MRH00105), Δz = 5.6 µm, 3x3 tiles; 20x air (CFI Plan Apo 20x λ, NA 0.75, MRD00205), Δz = 0.9 µm, 6x6 tiles. Reference beads on the glass (same well or neighbor wells) set the zero plane and allowed tilt correction.

### 4.7 Confocal z‐Stack Analysis of Gel Thickness

Confocal z‐stacks of fluorescent bead‐coated gels were quantified with a standalone Python workflow (flat_gel_surface_analyzer.py; versioned code in the project GitHub repository, link in Data Availability; algorithmic details and parameters in Supporting Information). ND2/TIFF stacks were loaded as (Z,Y,X) arrays and converted to per‐pixel top‐surface Z maps by locating the axial intensity maximum of the bead layer and refining its position with a sub‐slice parabolic fit. Heights were computed using Z_0_um_ as user‐defined axial reference (anchored experimentally when interpreting the mean Z as a thickness). A least‐squares plane was fit over a conservative core region to estimate residual tilt, and only the slope terms were removed. Summary metrics were reported per stack as the mean L‐filtered top‐surface Z (TopZ_LFilteredMean_um) and within‐well dispersion (TopZ_LFilteredStd_um), together with a band‐pass residual dispersion (BandpassStd_um) and QC fields (valid pixel fractions and truncation flags). Per‐folder outputs were exported as a single‐row‐per‐stack CSV plus run metadata for reproducibility; diagnostic figures were generated to support quality control. Thicknesses are reported as nominal z distances: because the dry objectives focus through the hydrated gel, physical thickness is larger by the axial factor (≈1.35–1.38 for the 4x and 10x objectives, NA ≤ 0.30, and ζ = 1.51 for the 20x objective, NA 0.75; Note S15), whereas comparisons between conditions, trends, and coefficients of variation are unaffected.

### 4.8 Culture of HT1080 Fibrosarcoma Cell Line

The human fibrosarcoma cell line (HT1080) used in this study was previously engineered to stably express the tubulin reporter RFP in addition to the F‐actin reporter LifeAct‐EGFP established in the commercial line (HT‐1080‐LifeAct‐TagGFP2, 40101, Ibidi). A genetically‐encoded variant of a FUCCI cell cycle sensor with CFP and iRFP signals for G1 and S/G2/M phases was also present in the cells. The cells were cultured using complete cell culture media, consisting of DMEM/F‐12 with L‐glutamine, HEPES, and phenol Red (11330032, Gibco), 10% v/v heat‐inactivated fetal bovine serum (FBS, 10270106, Gibco), and 1% v/v Penicillin‐Streptomycin (10 000 U mL^−^^1^, 15140‐122, Gibco), and passaged using Trypsin‐EDTA solution (T4049, Sigma–Aldrich) before the cells could reach 90% confluency. To prevent remaining trypsin during cell passaging from digesting the GelMA hydrogels, the cell suspensions were always centrifuged and resuspended in trypsin‐free media. All cell lines used in this study were tested negative for mycoplasma contamination.

### 4.9 Gel Compatibility With High‐Resolution Imaging

To verify that the photopatterned gels are optically clear and that the thickness is small enough to allow high‐resolution imaging with standard working distances (< 400 µm), HT1080 cells were seeded on the gels and imaged after two days of culture using a Crest V3 x‐Light spinning disk confocal microscope (Nikon) equipped with a CELESTA Light Engine source (TSx5030FV, Lumencor), a Photometrics Kinetix Scientific CMOS camera, and a 100x silicone oil objective (CFI SR HP Plan Apo Lambda S 100xC Sil, N.A. 1.35, WD 0.31 mm, MRD73950, Nikon). Specifically, images were acquired of actin and tubulin by excitation at 477 and 546 nm, respectively, with no binning (actin: 1 s exposure; tubulin: 200 ms exposure) at 100% source intensity (corresponding to 0.78 mW at 477 nm and 0.70 mW at 546 nm for the 100x objective; see Confocal excitation power calibration above).

### 4.10 Mechanical Characterization of the Gel Properties

To study the stiffness of photocrosslinked GelMA, large (>1 cm diameter) gels with GelMA concentration from 75 to 150 mg mL^−^^1^ were produced on glass bottom µ‐Dish 35 mm (81158, Ibidi), adapting the procedure described for the gel patterned in well plates. The UV doses used for the mechanical samples for 75, 100, and 150 µm were, respectively, 21, 13, and 8 mJ mm^−^^2^ to obtain gels with similar thickness above 50 µm based on the results from Figure 2h. After development and UV flooding, the gels were incubated in 2% w/v BSA in PBS for 4 h, and rinsed twice with PBS. Nanoindentation mechanical characterization was performed using a Chiaro nanoindenter (Optics11 Life, Amsterdam, The Netherlands). Specifically, probes comprising reflective cantilevers with stiffness of around 0.5 N/m and spherical tips with radii between 25 and 27 µm were used to perform indentation matrices with nominal gel indentation of 5 µm. 3.5 µm indentation capping was used to extract the contact modulus of the materials to avoid injecting final indentation depth variability in the modulus estimation. Each gel was sampled through an indentation matrix of at least 20 points spaced by at least 300 µm. Reported values are reduced (contact) moduli E^*^. Because the Poisson ratio was not characterized, they are upper bounds on the Young's modulus (Note S4). Indentations were treated as technical replicates nested within gels, and each gel was summarized by the median contact modulus across its indentation map. Statistical comparisons were made at the gel level on the gel medians. The increase of contact modulus with GelMA concentration was tested with an ordered‐alternative Jonckheere–Terpstra test across all twelve gels (three groups of four; exact p over the 34 650 distinct assignments). Production‐run and long‐term‐storage comparisons used an exact two‐sided Wilcoxon rank‐sum test on the log gel medians (Note S4).

### 4.11 Cell Area Extraction

20x confocal images of HT1080 Actin‐EGFP/Tubulin‐RFP cells cultured on glass and on LigHTS GelMA films (75 and 150 mg mL^−^^1^) were acquired with a 20x/0.75 NA air objective using 4x4 binning (actin: 477 nm, 50 ms exposure, 100% source intensity; tubulin: 546 nm, 200 ms exposure, 100% source intensity; corresponding specimen‐plane powers 5.8 mW at 477 nm and 4.9 mW at 546 nm for the 20x objective; see Confocal excitation power calibration). Images were analyzed with a custom, versioned Python workflow (project GitHub; extended rationale and validation in SI). Briefly, denoised ND2 images were segmented with a two‐channel Cellpose‐SAM model [66] using the actin and tubulin signals to generate whole‐cell masks; segmentation QC panels and label TIFFs were exported for traceability. Projected single‐cell areas (µm^2^) were computed from masks using microscope metadata for pixel scaling and aggregated per experiment x condition x FOV. All inference was performed on log‐transformed cell areas blocked at the level of the independent experiment (N = 3); cells were nested within fields of view (FOVs) and FOVs within experiments, with three FOVs (one per well) per condition per experiment. Each FOV was summarized by the median log‐area of its cells, so that condition was compared at the unit at which it was assigned rather than at the pseudoreplicated cell level. Pairwise comparisons between substrates (glass, 150 mg mL^−^^1^ GelMA, 75 mg mL^−^^1^ GelMA) used a cluster‐aware permutation test in which the condition labels of the FOV summaries were permuted within each experiment, preserving the balanced three‐vs.‐three block structure; the test statistic was the mean, across experiments, of the within‐experiment difference between the two conditions' FOV‐summary means. Because each block admits 20 arrangements, the null was enumerated exactly over all 20^3^ = 8000 within‐experiment label assignments, so no resampling floor applied; the design's exact two‐sided floor is 2/8000 = 2.5 × 10^−^^4^ before adjustment and 7.5 × 10^−^^4^ after Holm correction across the three contrasts. Effect sizes were reported as geometric‐mean area ratios, exp(mean within‐experiment log difference).

### 4.12 Photopatterning, Development, and UV Flooding of the Grooved GelMA Hydrogels

To produce grooved gels, the same methodology described for the preparation of the hydrogel precursor solution for flat GelMA was adopted. To generate a structured surface, a gradient illumination pattern was designed in Affinity Designer 2 consisting of a grayscale line array pattern matching the pixel count of PRIMO with intensities varying between 30% and 100% of the nominal UV dose. The illumination was performed in grayscale mode with Leonardo software tuning the exposure of the individual areas to reach the intended local dose. To map geometry control over grooved gels, different gradient illumination patterns were designed in Affinity Designer 2 as described, varying the pitch (21‐29‐40 µm in this study) and then exposed in PRIMO through Leonardo software with different selected local doses between 5 and 21 mJ mm^−^^2^. For contact guidance experiments, the grayscale lined pattern consisted of features of 20 µm width with 20 µm spacing. The gradient grayscale values corresponded to 30% to 100% of the selected local illumination of the tool, which was fixed at 8 mJ mm^−^^2^.

### 4.13 Image Analysis for Geometric Characterization

Grooved gels were coated with fluorescent beads as previously described and analyzed with a standalone Python workflow (groove_analyzer.py, versioned on GitHub; extended method and controls in SI). For PRIMO DMD‐patterned gels (Figure 4), groove metrics were computed on manually selected ∼1000 µm sub‐FOVs cropped from the 2x2 mosaic acquired at 20x, excluding tile boundaries to avoid stitching steps; these stacks were exported after NIS‐Elements denoising. For plate‐wide photomask exposures (Figures 5 and 6), sub‐FOVs of the same size were automatically center‐cropped from RAW stacks to enable batch processing. A paired control confirmed that analyzing RAW vs. NIS‐denoised stacks yielded indistinguishable pitch/depth/height within metrology uncertainty and did not change group‐level conclusions (see SI). Height maps were reconstructed per pixel by sub‐slice localization of the axial intensity maximum (quadratic refinement) and converted to µm using the z‐step; maps were optionally detrended by automated within‐FOV plane removal (no manual tilt adjustment), and no plate‐level curvature correction was applied. Groove orientation and a coarse pitch estimate were obtained from the dominant 2D Fourier component for alignment; groove pitch was reported as crest‐to‐crest spacing from peak detection on aligned band‐limited profiles, groove depth as crest‐to‐adjacent‐valleys amplitude averaged across periods (with a pitch‐dependent correction derived from synthetic validation), and grooved‐gel height/thickness as the median valley height to decouple bulk film height from periodic relief. Confocal groove depths are apparent optical values, because the dry objective compresses the axial scale inside the hydrated gel; physical groove depths are obtained with the axial factor ζ = 1.51 ± 0.12 determined against nanoindentation (Note S15).

### 4.14 Statistical Analysis

Group comparisons were one‐sided for Jonckheere–Terpstra and two‐sided for all other tests with α = 0.05 except where explicitly stated otherwise. For datasets with sufficient replicate counts, statistics were performed in Prism v10.4.2 (GraphPad) via Kruskal–Wallis test with Dunn's multiple comparisons. For experiments with small n (e.g., 3–4 independent gels per condition), p‐values were obtained using a permutation‐based Python workflow consistent with the reported experimental design, including Holm‐adjusted permutation slope tests, exact two‐sample permutation planned contrasts where feasible, and ordered‐alternative Jonckheere–Terpstra tests for monotone trends. Analysis scripts and the full per‐test outputs are available in the repository listed in the Data Availability Statement. For small balanced groups (N = 4 per condition), the exact two‐sample permutation space contains only C(8, 4) = 70 arrangements, imposing a minimum attainable two‐sided p‐value of 1/35 ≈ 0.029; after Holm adjustment across two or more contrasts, this floor can exceed α = 0.05 regardless of effect magnitude. Planned pairwise contrasts in this regime are therefore reported with bootstrap 95% confidence intervals, while global inference relies on the Jonckheere–Terpstra and permutation slope tests.

### 4.15 Statistics and Plotting Conventions

Unless otherwise indicated in the corresponding figure legend, data are plotted as mean ± SD (error bars), and all statistical tests are two‐sided except where explicitly specified otherwise with α = 0.05. Significance symbols (where used) follow: ^*^ *p* < 0.05, ^**^ *p* < 0.01, ^***^ *p* < 0.001.

### 4.16 Experimental Design

All fabrication conditions were processed in parallel under predefined illumination and formulation parameters. Randomization was not applied, since wells were not assigned to biological treatment groups but to defined fabrication conditions. Blinding was not performed, as image acquisition and quantitative analyses (surface reconstruction, cell area, migration tracking, holographic reconstruction) were conducted using automated, objective pipelines with fixed parameters applied identically across conditions. Image acquisition and analysis were automated with predefined QC flags and exclusion rules (e.g., confocal z‐stack truncation flags, insufficient valid‐pixel fraction, or focus drift/gel failure for migration time series; see SI for QC descriptors).

### 4.17 Contact‐Guidance Long‐Term Live Imaging Experiments

To verify that the hydrogel surface anisotropies were able to provide contact guidance to cells, HT1080 cells were seeded on LigHTS‐produced grooved gels and flat gels, and on glass pre‐coated with 40 µg mL^−^^1^ human plasma fibronectin (F0895, Sigma–Aldrich). Cell migration was observed with a 12‐h live imaging experiment (37°C, 90% relative humidity, 5% CO_2_). Specifically, the cells were cultured as described before, then 48 h before the migration assay, HT1080‐containing media was added to each well to seed roughly 1000 cells per well in the 384‐well plate format and roughly 250 cells per well in the 1536‐well plate format. Cells were left growing in the incubator until the start of the experiment.

To acquire the 12‐h migration experiments, a Crest V3 x‐Light spinning disk confocal microscope (Nikon) equipped with a CELESTA Light Engine source (TSx5030FV, Lumencor), a Photometrics Kinetix Scientific CMOS camera, and a 20x air objective (CFI Plan Apo 20x Lambda, N.A. 0.75, MRD00205, Nikon) was used. In terms of imaging conditions, the tubulin and actin signal in confocal mode and the transmitted white spectrum light in differential interference contrast (DIC) mode were recorded in a 15‐min‐interval time series for 12 h. Specifically, tubulin was imaged with excitation at 546 nm, 4x4 binning, 200 ms exposure at 100% source intensity (4.9 mW at the specimen plane for the 20x objective); actin was imaged with excitation at 477 nm, 4x4 binning, 50 ms exposure at 100% source intensity (5.8 mW at the specimen plane for the 20x objective); and DIC was imaged with 4x4 binning, 4 ms exposure. The fields of view that showed consistent focus through the 12 h and no mechanical failure in gels were subsequently analyzed.

### 4.18 Substrate‐Dependent Analysis of HT1080 Migration

To analyze migratory behavior of HT1080 cell line on different substrates, the confocal‐acquired raw time‐stacks were denoised using NIS‐Elements “Denoise” feature and analyzed as follows. First, the images were converted from the Nikon proprietary .nd2 format to .tiff files, and the channels were split for further analysis through an ImageJ macro. The .tiff file containing only the tubulin channel was further processed through custom Python scripts. The first analysis script uses Cellpose 3.0.5 pretrained cyto3 network to automatically segment the stacks given the user‐selected mean cell diameter [58], corresponding to 17 pixels in these experiments. The labels are imported in LapTrack (v 0.17.0) to generate tracks describing cell motion [59], tracks are saved in a .csv file for later use. To be able to visually check the cell segmentation and track attribution, a .mov movie overlaying the cell label border and its assigned track to the tubulin signal is created through matplotlib and saved.

The tracks contained in the .csv files are then used for quantitative analysis of cell movement on different substrates and between three independent experiments via another custom Python script. For each experiment, the script reads the tracks and calculates the parameter used to determine the contact guidance of grooves, called “2D nematic order parameter”. This parameter measures the deviation of the angle between the starting and ending point of the track segment (θ) from the vertical direction that corresponds to groove orientation: NO = cos (2^*^ (θ–90°)). By construction, this parameter varies between ‐1 if the cells migrate exclusively in the direction perpendicular to the grooves to 1 if all the movements are perfectly parallel to the grooves. Then, the script assigns a weighted nematic order (WNO) value to each track in the field of view, weighting the value of the single displacement with respect to its length. A synthetic WNO of the entire field of view is calculated, weighting again the WNO per track on its length. This parameter is used to compare each field of view with others recorded during the same experiment and among different experiments via a permutation test. In addition to the WNO, the code also calculates and compares the mean cell velocity of the field of view. All analysis results are reported in a .csv file, used to perform statistical analysis.

To perform statistical analysis, a custom Python routine is used. The script asks the user to select a .csv file containing a row per field‐of‐view (FOV) measurements across experimental groups. After user‐defined selection of the dependent variable, group labels, experiment identifiers, and optional FOV identifiers via a graphical interface, values are aggregated to yield one entry per experiment x group x FOV. Due to limited replication of experiments (N = 3, with n = 3 technical replicates per group per experiment), statistical testing relies on exact blocked label‐permutation, in which group labels were exhaustively permuted within each experiment while preserving sample sizes; the test statistic was the mean difference of group means across experiments, and two‐sided p‐values were obtained from the exact blocked label‐permutation distribution; p‐values were Holm‐adjusted where applicable. The code generates a dependent variable vs. groups plot highlighting the statistical difference among groups and a docx report of the statistical analysis performed.

### 4.19 Optimization of Hydrogel Preparation and Photopatterning for Parallel Processing

To perform upright large‐scale illumination with a commercial mask aligner setup (Karl Suss MA6; 365 nm; irradiance 3.5–3.7 mW cm^−^^2^ at the sample plane, measured with a power meter during the 16 min exposure), the plate had to be inverted during exposure. The precursor used for all plate‐wide exposures (Figures 5 and 6) was prepared from a commercial GelMA stock (PhotoGel‐INK 95% DS: 100 mg mL^−^^1^ GelMA, Type A, 300 Bloom, porcine, ∼95% degree of methacrylation, containing 2.5 mg mL^−^^1^ LAP; D16110025360, Cellink) diluted in PBS to a final GelMA concentration of 75 mg mL^−^^1^ with tartrazine at 4.5 mg mL^−^^1^. To ensure that the hydrogel precursor solution stayed in place, 10% v/v glycerol (Glycerol, 99.5%, 327255000, Thermo Scientific Chemicals) was added to the solution to increase the viscosity. Moreover, the LAP content was increased to 5 mg mL^−^^1^ to avoid polymerization‐induced phase separation (PIPS) associated with the significantly lower irradiance in this configuration compared to PRIMO DMD (20‐fold reduction). No modifications were made to the previously described procedures for gel development and UV flooding. To obtain grooved hydrogels, a custom‐made plate‐wide film photomask (JD photodata) featuring pitch design of 40, 60, and 80 µm was manually aligned to the wells and put in contact with a quartz block. The UV exposure was carried out in contact with the quartz for 16 min. For parallel produced gel characterization, the same procedures of fluorescent beads coating, confocal imaging, and quantitative analysis were carried out as described above.

### 4.20 Large‐Scale Compatible Characterization of Groove Profile by Holographic Phase Imaging

To enable rapid, label‐free quality control of LigHTS grooved hydrogels in high‐density plates, holographic phase imaging was acquired on a HoloMonitor M4 (Phase Holographic Imaging AB) positioned inside a cell incubator (37°C, 90% relative humidity, 5% CO_2_). Acquisition was controlled with HoloMonitor AppSuite (v4.0.1, Phase Holographic Imaging PHI AB). One field of view per well was imaged sequentially for gel QC, and for holographic migration imaging, the same field was sampled once every 5 min for 12 h. Imaging used an Olympus PLN 20x objective (NA 0.4) (N1215900, Evident) and a low‐power laser unit (635 nm, 0.2 mW cm^−^^2^). Wells were filled with PBS (+/+) to reduce air‐liquid interface curvature artifacts during surface profiling. Height‐map exports were processed with a dedicated Python workflow (holo_to_tiff.py and groove_analyzer_holo.py; versioned on the project GitHub repository; extended rationale and parameters in SI). In contrast to the confocal fluorescence pipeline (which reconstructs surface height from z‐stacks), holographic analysis operates on single‐plane phase/height maps: maps were decoded to native phase units (one unit = one wavelength of optical path) and stored on an uncalibrated height scale, detrended by within‐field plane removal to suppress slow curvature, and groove orientation plus a coarse pitch seed were obtained from the dominant spatial‐frequency component (2D FFT) for alignment. Groove pitch was reported as crest‐to‐crest spacing from peak detection on aligned, band‐limited profiles, and groove depth as crest‐to‐adjacent‐valleys amplitude averaged across periods; per‐well depth values were aggregated across wells to compute group CV. Physical groove depths in Figure 6f were measured on the median of the registered frames of each field with the estimator of Note S15 (375 of 382 fields passed quality control) and converted with C = 32 µm per native phase unit; Figure 6e shows a representative field.

### 4.21 Perturbation Assay on LigHTS‐Patterned Plate

To test whether LigHTS substrates support the compound‐perturbation step of a screening workflow, a single‐plate pharmacological experiment was run on a mask‐aligner‐patterned 384‐well plate that had been stored hydrated in PBS at 4°C for approximately one month. The 192 wells that had not been consumed by fluorescent‐bead or FITC‐poly‐L‐lysine metrology and carried flat or 60‐µm‐pitch grooved LigHTS hydrogels together with glass controls were used for the assay (Figure S12 and Figure 7). No additional extracellular matrix coating was applied before seeding, so cell attachment and the subsequent imaging also report the culture compatibility of the stored plate. The glass control wells were seeded and imaged alongside the hydrogel wells to test for cell viability after reseeding and handling, but were not further analyzed since the substrate‐by‐compound comparison they would serve needs replication that a single plate does not provide.

HT1080 reporter cells were seeded at approximately 1000 cells per well. After 24 h the complete DMEM/F12 medium was exchanged for serum‐free medium, and after a further 24 h the cells were treated with PP2 (0.1, 0.3, 1, 3, and 10 µm), mibefradil (0.3, 1, 3, 5, and 10 µm), nocodazole (0.01, 0.03, 0.1, 0.3, and 1 µm), or Y‐27632 (0.3, 1, 3, 10, and 30 µm), with three wells per concentration on each hydrogel geometry. Controls were 1:1000 DMSO, 1:1000 DMSO with FBS, 5 µm cytochalasin D, and 5 µm cytochalasin D with FBS, six wells each. The vehicle‐with‐FBS wells are considered the assay baseline, and the cytochalasin‐D‐with‐FBS wells the positive control. The two serum‐free controls excluded confounding factors due to starvation. Images were acquired with the same time loop on the same confocal setup as previously described, acquiring nuclear channel S/G2/M with excitation wavelength 638 nm, 4x4 binning, 100 ms exposure at 100% light intensity (9.3 mW power at the specimen plane for the 20x objective); tubulin was imaged with excitation at 546 nm, 4x4 binning, 50 ms exposure at 100% source intensity (7.6 mW at the specimen plane for the 20x objective); actin was imaged with excitation at 477 nm, 4x4 binning, 40 ms exposure at 100% source intensity (5.6 mW at the specimen plane for the 20x objective); nuclear channel G1 with excitation at 446 nm, 4x4 binning, 100 ms exposure at 100% source intensity (6.3 mW at the specimen plane for the 20x objective). Segmentation and tracking were performed as previously described in Note S10 with a 4‐channel extension to consider the nuclear cell cycle signals. Segmentation extracted the number of cells at each frame, then used to calculate cell growth progression over time, normalized to the first frame, enabled the calculation of cell aspect ratio as major‐axis length divided by minor‐axis length for cells grown on grooved substrates, and the tracking of its variation over time, cell displacement used to calculate velocity, and cell direction used to calculate the weighted nematic order. To express synthetically the extracted parameters, the 12h‐averaged migration velocity and the cell‐number growth are expressed as signed control‐referenced effect indices in which the matched substrate condition for vehicle is 0 and the matched substrate cytochalasin D treatment response is 100. These indices summarize relative change of the drug effect as suppression index (%) = 100 × (mean_C– mean_drug) / (mean_C– mean_C+), where “C ± ‐” indicates the serum‐free vehicle or drug control. Serum‐free formulations were used in this case to discriminate between drug and starvation effects. Cell‐cycle effects are reported separately as the percentage‐point difference in the S/G2/M fraction at 12 h relative to the matched vehicle‐with‐FBS condition: (%S/G2/M_12h ‐ %S/G2/M_0h)_drug – (%S/G2/M_12h ‐ %S/G2/M_0h)_C‐+ (Figure 7c). Cell‐cycle state was assigned from the dominant mean signal of the two FUCCI reporters.

### 4.22 Artificial Intelligence Generated Content Tools

The authors acknowledge the use of AI‐assisted tools, ChatGPT (OpenAI, 4.1 to 5.6) and Claude (Anthropic, Claude Opus 4.6 to 5), between 07/2025 and 08/2026. The tools supported development of the analysis code (drafting, refactoring, polishing, and debugging) for the Python workflows described in the Experimental Section and Supporting Information, and improved the clarity and readability of portions of the manuscript text throughout. AI tools were not used to generate, modify, or manipulate original experimental data or results. All AI‐assisted code and text outputs, including verification of claims, analyses, and citations, were manually reviewed, tested, and verified by the authors; analysis code was additionally validated against independent ground truth. No sensitive, proprietary, or identifiable human‐subject data were provided to the tools: this study used established cell lines only, and inputs were limited to author‐drafted text and analysis source code. The authors take full responsibility for the final manuscript, analyses, and code.

### 4.23 Ethics Statement

This study used established human cell lines and an established human induced pluripotent stem cell (hiPSC) line. No new human embryonic stem cell lines, embryos, or gametes were generated for this study. Use of the cell lines complied with institutional and applicable regulatory requirements. The hiPSC line used here (WTC11) is registered with hPSCreg under UCSFi001‐A‐1C.

## Author Contributions

A.E., S.R., and F.S.P. conceived the study, developed the methodology, and wrote the original draft. A.E. and S.R. performed the investigation, data curation, validation, and visualization. A.E., S.R., and J.Z. developed software. A.E., S.R., M.P., and E.T. performed formal analysis. A.E., M.P., M.D.S., and F.S.P. provided resources. A.E., F.A., and F.S.P. supervised the work. A.E. and F.S.P. acquired funding and administered the project. All authors reviewed and edited the manuscript.

## Conflicts of Interest

The authors declare no conflicts of interest.

## Supporting information




**Supporting File 1**: advs78192‐sup‐0001‐SuppMat.pdf.

**Supporting File 2**: advs78192‐sup‐0002‐VideoS1.mp4.

**Supporting File 3**: advs78192‐sup‐0003‐VideoS2.mov.

**Supporting File 4**: advs78192‐sup‐0004‐VideoS3.mp4.

**Supporting File 5**: advs78192‐sup‐0005‐VideoS4.mp4.

**Supporting File 6**: advs78192‐sup‐0006‐VideoS5.mp4.

**Supporting File 7**: advs78192‐sup‐0007‐VideoS6.mov.

**Supporting File 8**: advs78192‐sup‐0008‐VideoS7.mp4.

**Supporting File 9**: advs78192‐sup‐0009‐VideoS8.mp4.

**Supporting File 10**: advs78192‐sup‐0010‐VideoS9.mov.

## Data Availability

The data that support the findings of this study are openly available. Specifically, the raw imaging datasets are deposited in BioImage Archive (permanent accession number S‐BIAD4164). Nanoindentation data of flat and grooved gels as well as the statistical analysis of all the relevant datasets, are available on Zenodo (permanent DOI https://doi.org/10.5281/zenodo.22942992). Code and instructions to install and run analysis pipelines as described in the manuscript are published on GitHub and archived on Zenodo (permanent DOI https://doi.org/10.5281/zenodo.21840232).

## References

[1] A. Schuhmacher , M. Hinder , A. von Stegmann und Stein , D. Hartl , and O. Gassmann , “Analysis of Pharma R&D Productivity – A New Perspective Needed,” Drug Discovery Today 28, no. 10 (2023): 103726, 10.1016/j.drudis.2023.103726. 37506762

[2] D. Sun , W. Gao , H. Hu , and S. Zhou , “Why 90% of Clinical Drug Development Fails and How to Improve It?” Acta Pharmaceutica Sinica B 12, no. 7 (2022): 3049–3062, 10.1016/j.apsb.2022.02.002. PMC9293739 35865092

[3] M. S. Ringel , “What Is the Right Amount to Spend on Biopharma R&D?” Nature Reviews Drug Discovery 16, no. 9 (2017): 597–598, 10.1038/nrd.2017.114. 28685763

[4] R. Xie , V. Pal , Y. Yu , et al., “A Comprehensive Review on 3D Tissue Models: Biofabrication Technologies and Preclinical Applications,” Biomaterials 304 (2024): 122408, 10.1016/j.biomaterials.2023.122408. PMC10843844 38041911

[5] F. Foglietta , R. Canaparo , G. Muccioli , E. Terreno , and L. Serpe , “Methodological Aspects and Pharmacological Applications of Three‐Dimensional Cancer Cell Cultures and Organoids,” Life Sciences 254 (2020): 117784, 10.1016/j.lfs.2020.117784. 32416169

[6] G. Rossi , A. Manfrin , and M. P. Lutolf , “Progress and Potential in Organoid Research,” Nature Reviews Genetics 19, no. 11 (2018): 671–687, 10.1038/s41576-018-0051-9. 30228295

[7] L. A. Low , C. Mummery , B. R. Berridge , C. P. Austin , and D. A. Tagle , “Organs‐on‐Chips: Into the Next Decade,” Nature Reviews Drug Discovery 20, no. 5 (2021): 345–361, 10.1038/s41573-020-0079-3. 32913334

[8] L. Wang , D. Hu , J. Xu , J. Hu , and Y. Wang , “Complex in Vitro Model: A Transformative Model in Drug Development and Precision Medicine,” Clinical and Translational Science 17, no. 2 (2024): e13695, 10.1111/cts.13695. PMC10828975 38062923

[9] J. Carreras‐Puigvert and O. Spjuth , “Artificial Intelligence for High Content Imaging in Drug Discovery,” Current Opinion in Structural Biology 87 (2024): 102842, 10.1016/j.sbi.2024.102842. 38797109

[10] S. Sivanandan , B. Leitmann , E. Lubeck , et al., “A Pooled Cell Painting CRISPR Screening Platform Enables de Novo Inference of Gene Function by Self‐Supervised Deep Learning,” Nature Communications 17, no. 1 (2026v): 77, 10.1038/s41467-025-66778-6. PMC12770385 41402283

[11] A. Mullard , “Machine Learning Brings Cell Imaging Promises Into Focus,” Nature Reviews Drug Discovery 18, no. 9 (2019): 653–655, 10.1038/d41573-019-00144-2. 31477870

[12] S. Honarnejad , S. van Boeckel , H. van den Hurk , and S. van Helden , “Hit Discovery for Public Target Programs in the European Lead Factory: Experiences and Output From Assay Development and Ultra‐High‐Throughput Screening,” SLAS Discovery 26, no. 2 (2021): 192–204, 10.1177/2472555220942765. PMC8637381 32734803

[13] W. L. K. Chen and C. A. Simmons , “Lessons From (Patho)Physiological Tissue Stiffness and Their Implications for Drug Screening, Drug Delivery and Regenerative Medicine,” Advanced Drug Delivery Reviews 63, no. 4 (2011): 269–276, 10.1016/j.addr.2011.01.004. 21241759

[14] T. H. Booij , L. S. Price , and E. H. J. Danen , “3D Cell‐Based Assays for Drug Screens: Challenges in Imaging, Image Analysis, and High‐Content Analysis,” SLAS Discovery 24, no. 6 (2019): 615–627, 10.1177/2472555219830087. PMC6589915 30817892

[15] N. C. Peterson , P. K. Mahalingaiah , A. Fullerton , and M. Di Piazza , “Application of Microphysiological Systems in Biopharmaceutical Research and Development,” Lab on a Chip 20, no. 4 (2020): 697–708, 10.1039/C9LC00962K. 31967156

[16] J. Inglese , D. S. Auld , A. Jadhav , et al., “Quantitative High‐Throughput Screening: A Titration‐Based Approach That Efficiently Identifies Biological Activities in Large Chemical Libraries,” Proceedings of the National Academy of Sciences 103, no. 31 (2006): 11473–11478, 10.1073/pnas.0604348103. PMC1518803 16864780

[17] K. K. Kwon , J. Lee , H. Kim , D. Lee , and S. Lee , “Advancing High‐Throughput Screening Systems for Synthetic Biology and Biofoundry,” Current Opinion in Systems Biology 37 (2024): 100487, 10.1016/j.coisb.2023.100487.

[18] A. Buxboim , K. Rajagopal , A. E. X. Brown , and D. E. Discher , “How Deeply Cells Feel: Methods for Thin Gels,” Journal of Physics: Condensed Matter 22, no. 19 (2010): 194116, 10.1088/0953-8984/22/19/194116. PMC2864502 20454525

[19] C. G. Tusan , Y. Man , H. Zarkoob , et al., “Collective Cell Behavior in Mechanosensing of Substrate Thickness,” Biophysical Journal 114, no. 11 (2018): 2743–2755, 10.1016/j.bpj.2018.03.037. PMC6027966 29874622

[20] H. Yu and A. Pathak , “Emergent Depth‐Mechanosensing of Epithelial Collectives Regulates Cell Clustering and Dispersal on Layered Matrices,” Proceedings of the National Academy of Sciences 122, no. 37 (2025): ee2423875122, 10.1073/pnas.2423875122. PMC12452895 40932776

[21] M. Mirbagheri , V. Adibnia , B. R. Hughes , S. D. Waldman , X. Banquy , and D. K. Hwang , “Advanced Cell Culture Platforms: A Growing Quest for Emulating Natural Tissues,” Materials Horizons 6, no. 1 (2019): 45–71, 10.1039/C8MH00803E.

[22] Y. Sun , R. Deng , X. Ren , K. Zhang , and J. Li , “2D Gelatin Methacrylate Hydrogels With Tunable Stiffness for Investigating Cell Behaviors,” ACS Applied Bio Materials 2, no. 1 (2019): 570–576, 10.1021/acsabm.8b00712. 35016320

[23] M. Luciano and S. Gabriele , “Designing Hydrogel Dimensionality to Investigate Mechanobiology,” Soft Matter 21, no. 23 (2025): 4551–4572, 10.1039/D4SM01458H. 40439624

[24] W. Y. Wang , A. T. Pearson , M. L. Kutys , et al., “Extracellular Matrix Alignment Dictates the Organization of Focal Adhesions and Directs Uniaxial Cell Migration,” APL Bioengineering 2, no. 4 (2018): 046107, 10.1063/1.5052239. PMC6481732 31069329

[25] K. Lu , Y. Qian , J. Gong , et al., “Biofabrication of Aligned Structures That Guide Cell Orientation and Applications in Tissue Engineering,” Bio‐Design and Manufacturing 4, no. 2 (2021): 258–277, 10.1007/s42242-020-00104-5.

[26] B. Zhang , Y. Luo , X. Zhou , L. Gao , X. Yin , and H. Yang , “GelMA Micropattern Enhances Cardiomyocyte Organization, Maturation, and Contraction via Contact Guidance,” APL Bioengineering 8, no. 2 (2024): 026108, 10.1063/5.0182585. PMC11065454 38699629

[27] T. Rossy , L. Schwendeman , S. Kohli , et al., “Leveraging Microtopography to Pattern Multi‐Oriented Muscle Actuators,” Biomaterials Science 13, no. 11 (2025): 2891–2907, 10.1039/D4BM01017E. PMC11907412 40084556

[28] A. A. Conner , Y. Yao , S. W. Chan , et al., “High‐Throughput Analysis of Topographical Cues for the Expansion of Murine Pluripotent Stem Cells,” Nanotechnology 35, no. 45 (2024): 455101, 10.1088/1361-6528/ad6994. 39084233

[29] R. Lei , E. A. Akins , K. C. Y. Wong , et al., “Multiwell Combinatorial Hydrogel Array for High‐Throughput Analysis of Cell‐ECM Interactions,” ACS Biomaterials Science & Engineering 7, no. 6 (2021): 2453–2465, 10.1021/acsbiomaterials.1c00065. PMC8900525 34028263

[30] P. B. Lücker , S. Javaherian , J. P. Soleas , D. Halverson , P. W. Zandstra , and A. P. McGuigan , “A Microgroove Patterned Multiwell Cell Culture Plate for High‐Throughput Studies of Cell Alignment,” Biotechnology and Bioengineering 111, no. 12 (2014): 2537–2548, 10.1002/bit.25298. 24889796

[31] J. D. Mih , A. S. Sharif , F. Liu , A. Marinkovic , M. M. Symer , and D. J. Tschumperlin , “A Multiwell Platform for Studying Stiffness‐Dependent Cell Biology,” PLOS ONE 6, no. 5 (2011): 19929, 10.1371/journal.pone.0019929. PMC3103526 21637769

[32] N. R. M. Beijer , A. S. Vasilevich , B. Pilavci , et al., “TopoWellPlate: A Well‐Plate‐Based Screening Platform to Study Cell–Surface Topography Interactions,” Advanced Biosystems 1, no. 4 (2017): 1700002, 10.1002/adbi.201700002. 32646161

[33] F. V. Almeida , L. Gammon , A. C. Laly , O. J. Pundel , C. L. Bishop , and J. T. Connelly , “High‐Content Analysis of Cell Migration Dynamics Within a Micropatterned Screening Platform,” Advanced Biosystems 3, no. 8 (2019): 1900011, 10.1002/adbi.201900011. 32648701

[34] J. Hu , A. A. Gondarenko , A. P. Dang , et al., “High‐Throughput Mechanobiology Screening Platform Using Micro‐ and Nanotopography,” Nano Letters 16, no. 4 (2016): 2198–2204, 10.1021/acs.nanolett.5b04364. PMC5403373 26990380

[35] Advanced BioMatrix , CytoSoftTM Imaging, 96‐Well Plates (0.2 ‐ 64 kPa), accessed, 2025, https://advancedbiomatrix.com/cytosoft‐96well.html.

[36] Softwell 384 Glass , Matrigen, accessed, 2026, https://store.matrigen.com/products/softwell‐384‐glass.

[37] E. Torchia , M. Di Sante , B. Horda , et al., “Fabrication of Cell Culture Hydrogels by Robotic Liquid Handling Automation for High‐Throughput Drug Testing,” Communications Engineering 4, no. 1 (2025): 222, 10.1038/s44172-025-00575-3. PMC12749810 41430373

[38] D. Chitsaz and T. E. Kennedy , “High‐Throughput Microcontact Printing of Proteins in Microwell Cell Culture Plates,” MethodsX 12 (2024): 102665, 10.1016/j.mex.2024.102665. PMC10957495 38524307

[39] M. Bril , M. D'Urso , M. Prejanò , M. Costantini , L. Angeloni , and N. A. Kurniawan , “Digital Photoinduced Topographical Microsculpting of Hydrogels,” Advanced Materials Technologies 9, no. 23 (2024): 2400721, 10.1002/admt.202400721.

[40] M. L. Skelton , J. L. Gentry , L. R. Astrab , et al., “Modular Multiwell Viscoelastic Hydrogel Platform for Two‐ and Three‐Dimensional Cell Culture Applications,” ACS Biomaterials Science & Engineering 10, no. 5 (2024): 3280–3292, 10.1021/acsbiomaterials.4c00312. PMC11094681 38608136

[41] C. Melero , A. Kolmogorova , P. Atherton , et al., “Light‐Induced Molecular Adsorption of Proteins Using the PRIMO System for Micro‐Patterning to Study Cell Responses to Extracellular Matrix Proteins,” Journal of Visualized Experiments (JoVE), no. 152 (2019): e60092, 10.3791/60092. 31657796

[42] Y. Li , Q. Mao , J. Yin , Y. Wang , J. Fu , and Y. Huang , “Theoretical Prediction and Experimental Validation of the Digital Light Processing (DLP) Working Curve for Photocurable Materials,” Additive Manufacturing 37 (2021): 101716, 10.1016/j.addma.2020.101716.

[43] A. Pariskar , P. K. Sharma , U. S. Murty , and S. Banerjee , “Effect of Tartrazine as Photoabsorber for Improved Printing Resolution of 3D Printed “Ghost Tablets”: Non‐Erodible Inert Matrices,” Journal of Pharmaceutical Sciences 112, no. 4 (2023): 1020–1031, 10.1016/j.xphs.2022.11.014. 36410417

[44] R. Levato , K. S. Lim , W. Li , et al., “High‐Resolution Lithographic Biofabrication of Hydrogels With Complex Microchannels From Low‐Temperature‐Soluble Gelatin Bioresins,” Materials Today Bio 12 (2021): 100162, 10.1016/j.mtbio.2021.100162. PMC8626672 34870141

[45] M. Di Sante , M. Pezzotti , J. Zimmermann , et al., “CALIPERS: Cell Cycle‐Aware Live Imaging for Phenotyping Experiments and Regeneration Studies,” Nature Communications 17 (2026): 9306, 10.1038/s41467-026-76144-9. PMC13526800 42669673

[46] M. Pezzotti , E. Torchia , J. Zimmermann , et al., “A Vertically Integrated System for Tracking and Assessing Cell‐Cycle‐Aware Phenotypes Under Confinement,” APL Bioengineering 10, no. 1 (2026): 016114, 10.1063/5.0306480. PMC12962752 41799556

[47] Y. Yagci , S. Jockusch , and N. J. Turro , “Photoinitiated Polymerization: Advances, Challenges, and Opportunities,” Macromolecules 43, no. 15 (2010): 6245–6260, 10.1021/ma1007545.

[48] B. D. Fairbanks , M. P. Schwartz , C. N. Bowman , and K. S. Anseth , “Photoinitiated Polymerization of PEG‐Diacrylate With Lithium Phenyl‐2,4,6‐Trimethylbenzoylphosphinate: Polymerization Rate and Cytocompatibility,” Biomaterials 30, no. 35 (2009): 6702–6707, 10.1016/j.biomaterials.2009.08.055. PMC2896013 19783300

[49] H. Gong , M. Beauchamp , S. Perry , A. T. Woolley , and G. P. Nordin , “Optical Approach to Resin Formulation for 3D Printed Microfluidics,” RSC Advances 5, no. 129 (2015): 106621–106632, 10.1039/c5ra23855b. PMC4699590 26744624

[50] R. Simič , J. Mandal , K. Zhang , and N. D. Spencer , “Oxygen Inhibition of Free‐Radical Polymerization is the Dominant Mechanism Behind the “Mold Effect” on Hydrogels,” Soft Matter 17, no. 26 (2021): 6394–6403, 10.1039/D1SM00395J. PMC8262556 34132302

[51] A. J. Lehtonen , O. Arasalo , L. Srbova , M. Heilala , and J. Pokki , “Magnetic Microrheometry of Tumor‐Relevant Stiffness Levels and Probabilistic Quantification of Viscoelasticity Differences Inside 3D Cell Culture Matrices,” PLOS ONE 18, no. 3 (2023): e0282511, 10.1371/journal.pone.0282511. PMC10032533 36947558

[52] S. G. Zambuto , S. S. Kolluru , E. Ferchichi , et al., “Evaluation of Gelatin Bloom Strength on Gelatin Methacryloyl Hydrogel Properties,” Journal of the Mechanical Behavior of Biomedical Materials 154 (2024): 106509, 10.1016/j.jmbbm.2024.106509. PMC12662645 38518513

[53] E. S. Krivega , S. L. Kotova , P. S. Timashev , and Y. M. Efremov , “Mechanical Characterization of Soft Biomaterials: Which Time and Spatial Scale to Choose?” Soft Matter 20, no. 26 (2024): 5095–5104, 10.1039/d4sm00530a. 38888165

[54] N. Gavara , “Combined Strategies for Optimal Detection of the Contact Point in AFM Force‐Indentation Curves Obtained on Thin Samples and Adherent Cells,” Scientific Reports 6, no. 1 (2016): 21267, 10.1038/srep21267. PMC4759531 26891762

[55] D. Xu , M. L. Hernandez Miranda , N. D. Evans , B. G. Sengers , M. Browne , and R. B. Cook , “Depth Profiling via Nanoindentation for Characterisation of the Elastic Modulus and Hydraulic Properties of Thin Hydrogel Layers,” Journal of the Mechanical Behavior of Biomedical Materials 148 (2023): 106195, 10.1016/j.jmbbm.2023.106195. 37862727

[56] A. Zand , J. M. Macharia , I. Szabó , et al., “The Impact of Tartrazine on DNA Methylation, Histone Deacetylation, and Genomic Stability in Human Cell Lines,” Nutrients 17, no. 5 (2025): 913, 10.3390/nu17050913. PMC11902176 40077783

[57] D. D. Thanh , N. Bich‐Ngoc , C. Paques , et al., “The Food Dye Tartrazine Disrupts Vascular Formation Both in Zebrafish Larvae and in Human Primary Endothelial Cells,” Scientific Reports 14, no. 1 (2024): 30367, 10.1038/s41598-024-82076-5. PMC11621646 39639097

[58] C. Stringer and M. Pachitariu , “Cellpose3: One‐Click Image Restoration for Improved Cellular Segmentation,” Nature Methods 22, no. 3 (2025): 592–599, 10.1038/s41592-025-02595-5. PMC11903308 39939718

[59] Y. T. Fukai and K. Kawaguchi , “LapTrack: Linear Assignment Particle Tracking With Tunable Metrics,” Bioinformatics 39 (2023): btac799, 10.1093/bioinformatics/btac799. PMC9825786 36495181

[60] D. V. Lyakin , L. A. Maksimova , A. Y. Sdobnov , and V. P. Ryabukho , “The Influence of the Numerical Aperture of a Beam Probing an Object on the Determination of the Thickness of a Layered Object in Confocal Microscopy,” Optics and Spectroscopy 123, no. 3 (2017): 487–494, 10.1134/S0030400X17090235.

[61] T. H. Besseling , J. Jose , and A. vean Blaaderen , “Methods to Calibrate and Scale Axial Distances in Confocal Microscopy as a Function of Refractive Index,” Journal of Microscopy 257, no. 2 (2015): 142–150, 10.1111/jmi.12194. PMC4383648 25444358

[62] V. Niggli , M. Schmid , and A. Nievergelt , “Differential Roles of Rho‐Kinase and Myosin Light Chain Kinase in Regulating Shape, Adhesion, and Migration of HT1080 Fibrosarcoma Cells,” Biochemical and Biophysical Research Communications 343, no. 2 (2006): 602–608, 10.1016/j.bbrc.2006.03.022. 16554034

[63] J. Huang , A. L. Kindzelskii , A. J. Clark , and H. R. Petty , “Identification of Channels Promoting Calcium Spikes and Waves in HT1080 Tumor Cells: Their Apparent Roles in Cell Motility and Invasion,” Cancer Research 64, no. 7 (2004): 2482–2489, 10.1158/0008-5472.CAN-03-3501. 15059902

[64] W. Wang , Y. Liu , and K. Liao , “Tyrosine Phosphorylation of Cortactin by the FAK‐Src Complex at Focal Adhesions Regulates Cell Motility,” BMC Cell Biology 12, no. 1 (2011): 49, 10.1186/1471-2121-12-49. PMC3245448 22078467

[65] W. Chao and J. Kunz , “Focal Adhesion Disassembly Requires Clathrin‐Dependent Endocytosis of Integrins,” FEBS Letters 583, no. 8 (2009): 1337–1343, 10.1016/j.febslet.2009.03.037. PMC2801759 19306879

[66] M. Pachitariu , M. Rariden , and C. M. Stringer , “Cellpose‐SAM: Superhuman Generalization for Cellular Segmentation,” preprint, bioRxiv, May 1, 2025, 10.1101/2025.04.28.651001.

