# Supplementary material for: LigHTS: Biomimetic Hydrogel Photo‐Fabrication for Imaging‐Based Ultra‐High‐Throughput Screening
Source: Adv Sci (Weinh). 2026 Oct 12:e78192. Online ahead of print. doi: 10.1002/advs.78192 (PMC13658890; doi:10.1002/advs.78192)
Supplement: Supplementary file 1 [file ADVS-9999-e78192-s009.pdf]

Supporting Information

LigHTS: Biomimetic Hydrogel Photo-Fabrication for Imaging-Based Ultra-High-Throughput Screening

Alessandro Enrico[†,,1], Sara Rigolli[†,1], Julius Zimmermann[1], Melissa Pezzotti[1], Eloisa Torchia[1], Moises Di Sante[2], Ferdinando Auricchio[3], Francesco S. Pasqualini[1,*]*

SI Table of Contents

Number of pages: 40
Number of videos: 9
Number of figures: 12
Number of notes: 16

Video S1 - Tartrazine suppresses meniscus formation and enables predictable dose-dependent thickness in flat GelMA films. Representative gel topographies reconstructed from spinning-disk confocal z-stacks (4x objective) for absorber-free and absorber-containing hydrogel precursor formulations (GelMA 75 mg mL^{-1}, LAP 2.5 mg mL^{-1}, 10% (v/v) glycerol in PBS). Isotropic substrates were polymerized at three UV doses (columns: 5, 8, 13 mJ mm^{-2}) in the absence (top row: 0 mg mL^{-1} tartrazine) or presence (bottom row: 2.25 mg mL^{-1} tartrazine) of absorber. Without absorber, the exposure drives bulk curing and yields meniscus-dominated hydrogel profile. With tartrazine, meniscus effects are suppressed, producing flatter substrates with thickness tunable by UV dose (Figure 2b-g). For height reconstruction, fluorescent beads were electrostatically adsorbed on the formed gels to localize the gel-liquid surface, and nearby gel-free reference areas were used to establish the z position of the glass interface (not shown in the video). Areas with fewer or no beads do not point to gel defects, but inhomogeneities in the beads coating. The stitched illumination required by the focusing optics produces a visible tile-like inhomogeneity of the gel surface. This artifact is intrinsic to focusing-optics-based photopatterning, and we avoid it by combining tartrazine-containing precursor solutions with collimated light (Figure 5 and 6). Confocal acquisition settings: 4x objective; 4x4 binning; red excitation (638 nm), 50 ms exposure, 100% source intensity; camera settings (including gain) held constant. Scale bars: 500 µm. (MP4)

Video S2 - Automated liquid handling removes soluble tartrazine from a 384-well plate. Recording of the automated development sequence, played at four times real time, performed on a 384-well plate filled with tartrazine-rich MES buffer as a model of the post-exposure well content. In each cycle the pipetting robot draws fresh MES from a reservoir, dispenses it into the wells, mixes three times by pipetting, and aspirates. The recovered liquid is transferred to a 96-well collection plate for color evaluation and the cycle repeats. The yellow tartrazine signal fades progressively over the six cycles shown (Note S2; Figure S2). (MP4)

Video S3 - High-absorber formulation yields thin, laterally uniform films across UV doses. Representative gel topographies reconstructed from spinning-disk confocal z-stacks (20x objective) for flat GelMA substrates prepared at the highest tartrazine condition used in this work (9 mg mL^{-1} tartrazine). Hydrogels were polymerized at three UV doses (panels: 5, 8, 13 mJ mm^{-2}), illustrating absorber-guided confinement of curing, producing gel thickness in the ideal range for imaging-based biomimetic scaffolds (10-100 µm), and improved within-field thickness uniformity in the thin-film regime (Figure 2g). Confocal acquisition settings: 20x objective; 4x4 binning; red excitation (638 nm), 50 ms exposure, 100% source intensity (5.5 mW at the specimen plane for the 20x objective; see Main Text Experimental Section). Scale bars: 500 µm. (MP4)

Video S4 - GelMA concentration and UV dose co-tune flat-film thickness under fixed absorber and initiator. Representative gel topographies reconstructed from spinning-disk confocal z-stacks (20x objective) for flat GelMA films spanning GelMA concentration (rows: 200, 150, and 75 mg mL^{-1}, as labeled) and UV dose (columns: 5, 8, and 13 mJ mm^{-2}) at fixed tartrazine (4.5 mg mL^{-1}) and LAP (1 mg mL^{-1}) in glycerol-free PBS. Across the matrix, increasing GelMA content and UV dose shift the gel thickness, defining the formulation window used to jointly tune thickness and mechanics (Figure 2h,i). Confocal acquisition settings: 20x objective; 4x4 binning; red excitation (655 nm), 50 ms exposure, 100% source intensity (638 nm calibration at 20x: 5.5 mW at the specimen plane; see Main Text Experimental Section). Scale bars: 200 µm. (MP4)

Video S5 - HT1080 migration is contact-guided on grooved gels. Time-lapse confocal imaging of HT1080 fibrosarcoma cell migration on fibronectin-coated glass (left), flat gels (center), and grooved gels (right). The fluorescent tubulin signal (gray) was acquired with a 20x objective every 15 min over 12 h. Overlays show Cellpose segmentation masks and LapTrack trajectories used to quantify migration alignment (displacement-weighted nematic order, WNO; Figure 4h). Manual inspection of the segmentation masks revealed minimal need for manual curation, which was performed only on the videos for visualization purposes. Cells on fibronectin-coated glass and flat gels displayed no preferred orientation in their trajectories, whereas cells on grooved gels predominantly migrated along the groove axis. Confocal acquisition settings: 20x objective; 4x4 binning; tubulin channel excitation at 546 nm, 200 ms exposure, 100% source intensity (4.9 mW at the specimen plane for the 20x objective; see Main Text Experimental Section). Scale bars: 100 µm. (MP4)

Video S6 - Spontaneously beating hiPSC-derived cardiomyocytes tissue on grooved LigHTS hydrogels. Fluorescence time-lapse of human induced pluripotent stem cell-derived cardiomyocytes 5 days after seeding at high confluence on fibronectin-coated, 40-µm-pitch grooved hydrogels prepared from 150 mg mL^{-1} GelMA precursor. Live-cell fluorescent probes for actin (gray) and intracellular Ca^{2+} (green) allowed direct live imaging with a 20x objective at 10 frames s^{-1} over 10 s. The monolayer is partially aligned along the groove axis and contracts spontaneously and synchronously across the field of view, showing that the grooved GelMA hydrogel produced using the LigHTS method supports cell viability as well as the structural organization and the contractile function of a mechanosensitive hiPSC-derived lineage (Note S11; Figure S8h). Scale bar: 100 µm. (MOV)

Video S7 - Mask-aligner patterned grooved gels enable migration assays at high-throughput scale. Time-lapse confocal imaging of HT1080 migration on grooved GelMA substrates fabricated in parallel by mask-aligner exposure, where collimated light from a conventional UV lamp was used to photostructure the hydrogel surface by interposing a film photomask encoding groove designs with 40, 60, and 80 µm pitch (left, center, and right, respectively). The migration assay and analysis match Figure 4g-h and Video S5, demonstrating the successful translation from low-throughput DMD patterning to scalable photomask-based fabrication for the dataset in Figure 6d. Confocal acquisition settings: 20x objective; 4x4 binning; tubulin channel excitation at 546 nm, 200 ms exposure, 100% source intensity (4.9

mW at the specimen plane for the 20x objective; see Main Text Experimental Section). Scale bars: 100 µm. (MP4)

Video S8 - Label-free holographic phase imaging enables simultaneous groove-field quality control and migration tracking. Time-lapse holographic phase imaging (HoloMonitor M4; 20x air objective; 635 nm) of HT1080 cells on grooved GelMA substrates fabricated by collimated UV exposure through film photomasks with 40, 60, and 80 µm pitch (left, center, and right; same fabrication method as for the hydrogels in Video S7). The grooves (static substrate component) were extracted by a temporal-median projection of the image stack and rendered in cyan for rapid topography/QC readout (top row; Figure 6e,f), while the frame-to-median residual was rendered in magenta to isolate moving cells for segmentation and migration tracking using the same Cellpose / LapTrack workflow as in Videos S5 and S7 (bottom row). Holographic acquisition settings: HoloMonitor M4 controlled with HoloMonitor AppSuite v4.0.1 inside a cell incubator (37 °C, 90% relative humidity, 5% CO_2); 20x objective (NA 0.4); 635 nm illumination at 0.2 mW cm^{-2}; one field of view per well sampled once every 5 min for 12 h (see Main Text Experimental Section). Scale bars: 100 µm. (MP4)

Video S9 - Multiparametric pharmacological perturbation assay on flat and grooved LigHTS substrates. Time-lapse of HT1080 reporter cells over the 12 h treatment window, sampled every 15 min and played at 4 frames s^{-1}. Columns show the four compounds and the two controls (PP2, mibefradil, nocodazole, Y-27632, 1:1000 DMSO vehicle, and 5 µM cytochalasin D); rows show flat hydrogels at the lowest and highest concentration of each compound (top) and 60-µm-pitch grooved hydrogels at the same concentrations (bottom). The vehicle and cytochalasin D wells were supplemented with FBS. Each panel overlays the Cellpose segmentation outlines and LapTrack trajectories used for the migration tracking on the four-channel fluorescence composite, with actin in gray, tubulin in green, and the G1 and S/G2/M FUCCI reporters in cyan and magenta; trajectory colors identify individual tracks. Cells on grooved substrates migrate along the groove axis under vehicle and at the lowest concentrations, whereas nocodazole at the highest concentration rounds the cells and suppresses both elongation and displacement on either geometry (Figure 7). Scale bars: 100 µm. (MP4)

Note S1. Estimation of the molar extinction coefficients at 365 nm for tartrazine and LAP, and assessment of tartrazine photostability under illumination

Photophysical principles of dose-controllable curing depth: to establish the photophysical basis for dose-controllable curing through competitive photon absorption, we quantified the attenuation of 365 nm light by tartrazine (non-reactive absorber) and LAP (photoinitiator) under the same in-well optical path used for photopolymerization. Specifically, we performed single-pass transmission measurements at 365 nm through the 0.8 mm recess geometry of a 96-well plate (**Figure S1a-c**). We then used the concentration dependence of transmission to estimate effective molar extinction coefficients (ε_{365}). In parallel, transmission through tartrazine solution was monitored during continuous illumination to test for measurable photobleaching over exposure-relevant timescales (5 minutes; **Figure S1d**).

Transmission assay and sample preparation: transmission of UV light through PBS containing 10% (v/v) glycerol was compared to solutions of tartrazine or LAP at the concentrations shown in the plate map (Figure S1b). For each condition, 10 µL was dispensed into the central recess of an Ibidi µ-Plate 96 Well 3D (defined optical path length l = 0.8 mm = 0.08 cm). Empty wells provided the normalization reference (I_0), and buffer-only wells were included as a solvent blank. The sample was illuminated at 365 nm through the same 4x objective used for patterning (Figure S1a), and transmitted power (I) was recorded above the well using a power meter (Argo-POWER HM Slide V2, Argolight). In Figure S1c, $T = I/I_{empty}$ where I is the transmitted power measured through the liquid-filled cavities and I_{empty} is the transmitted power measured through an empty well (100% reference), and each point corresponds to one independent well measurement, time averaged over a stable plateau of at least 8 s (median 14 s; n as reported in the Figure S1 caption). For the photostability check (Figure S1d), transmitted power was recorded continuously for 5 minutes under constant illumination and normalized to the initial value for that well.

Beer-Lambert model and ε_{365} estimation: normalized transmission was defined as $T = I/I_0$ where I is the transmitted power measured through the sample and I0 is the transmitted power measured through a solvent blank (PBS + 10% v/v glycerol) in the same plate/optical configuration. Absorbance at 365 nm was calculated as $A_{365} = -\log_{10}(T)$. Mass concentrations were converted to molar concentration c via c [M] = (C_{mass} [mg mL^{-1}] × 1 g L^{-1} per mg mL^{-1}) / MW, using MW = 534.36 g mol^{-1} for tartrazine and MW = 294.10 g mol^{-1} for LAP. Assuming single-pass attenuation through an optical path length (800 µm), absorbance scales with concentration: $A_{365} = \varepsilon_{365} \cdot c \cdot l + A0$, where A_0 captures concentration-independent

losses in this plate/optics configuration (interfaces and solvent baseline). Effective ε_{365} values were obtained by least-squares linear regression of A_{365} versus c, with ε_{365} = (slope)/l. For tartrazine, the fit was performed only on 0.1 (n= 6 wells) and 1 mg mL^{-1} (n = 12 wells), because higher tartrazine concentrations yielded transmitted powers near the measurement noise floor (no reliably measurable transmission with the sensor in this configuration). For LAP, the fit included all three tested concentrations (1, 2.5, and 5 mg mL^{-1}). This workflow yields $\varepsilon_{365} \approx 1 \times 10^4$ M^{-1} cm^{-1} for tartrazine and $\varepsilon_{365} \approx 2.2 \times 10^2$ M^{-1} cm^{-1} for LAP, consistent with absorber-dominated attenuation at 365 nm under LigHTS operating concentrations. The time traces in Figure S1d separate the absorber from the photoinitiator. Transmission through 0.1 mg mL^{-1} tartrazine fell by about 5% of its initial value during the first 1.5 min and then held constant for the remainder of the 5 min. Tartrazine therefore does not bleach progressively on the minute timescale of the photopolymerization protocol used here.

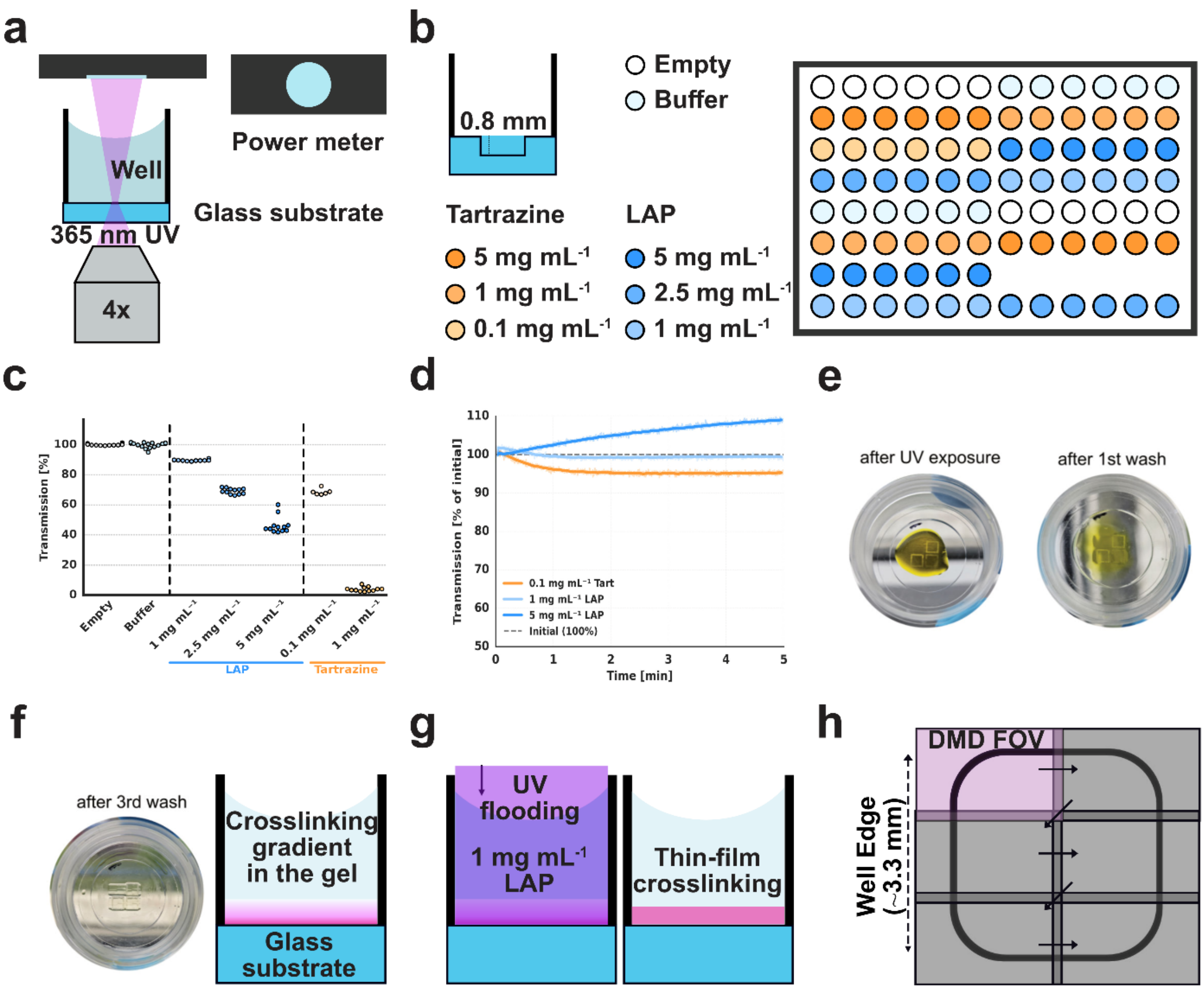

Figure S1. UV transmission controls and workflow for absorber-guided in-well photopolymerization. a) Optical configuration used to pattern gels and to quantify transmission through different media at 365 nm. UV light is delivered through a 4x objective

to the glass-bottom well and transmitted power is recorded above the sample with a power meter (Argo-POWER HM Slide V2, Argolight, schematic not to scale). b) Plate layout and solution set for transmission screening using an Ibidi µ-Plate 96 Well 3D, whose inner well compartment (central recess) has a defined depth of 0.8 mm. Liquid cast in this geometry fills as a near-cylindrical column before reaching the outer wall, mitigating meniscus formation. 0.8 mm was used as the effective optical path length for Beer-Lambert-based estimates. Solutions were prepared in PBS containing 10% (v/v) glycerol. Color code: Empty (white) and Buffer (light gray) controls; tartrazine (orange gradient; 0.1, 1, 5 mg mL^{-1}); LAP (blue gradient; 1, 2.5, 5 mg mL^{-1}). c) Single-pass UV transmission for each condition, reported as Transmission (%) and normalized to the empty-well reference (100%). Each point corresponds to a single independent well measurement (n = 12 wells per condition, except for n = 6 wells for 0.1 mg mL^{-1} tartrazine) time averaged for roughly 20 s. Dashed separators delineate controls, LAP-only, and tartrazine-only groups. d) Time stability of transmission under continuous illumination (example traces; 5 min), plotted as transmission normalized to the initial value (100%), to assess short-timescale photostability/photoproduct effects during exposure. Representative conditions are labeled (e.g., 0.1 mg mL^{-1} tartrazine, 1 and 5 mg mL^{-1} LAP). e-f) Representative photographs of tartrazine-containing gel patterned and developed in a 35 mm glass-bottom dish (used here to ease visualization), shown e) after UV exposure and after sequential rinses (after 1st wash, after 3rd wash, panel f) to document absorber washout during development. g) Conceptual workflow illustrating absorber-guided depth control: after tartrazine removal, a depth-dependent crosslinking profile can remain ("crosslinking gradient in the gel"); a subsequent UV flood step in LAP-containing buffer (example shown: 1 mg mL^{-1} LAP) is used to homogenize crosslinking through the film thickness. h) Tiled illumination for full-well patterning. The rounded-corner square outlined with the thick black line marks the well footprint (~3.3 mm edge for 384-well plates). The shaded rectangle is the single projected DMD field of view using a 4x objective, and the offset outlines are the translated exposure positions required to cover the whole well, showing that full-well patterning needs lateral stepping beyond one exposure field and that stitching between fields results in dose inhomogeneity within the well.

Note S2. Automated removal of soluble tartrazine during hydrogel development

Development requires the soluble absorber to be washed out of the gels, a step we perform manually by pipetting and vacuum aspiration. We tested whether repeated dispense, mix, and aspirate cycles reach the same endpoint using a liquid handling robot without integrated vacuum aspiration. A 384-well plate was filled with a solution of 15 mM pH 6 2-(N-morpholino)ethanesulfonic acid (MES) buffer with 9 mg mL^{-1} tartrazine as a model of the post-exposure well content, and the wash sequence was executed on a benchtop pipetting robot (Assist Plus, INTEGRA Biosciences) with 8-channel Voyager (4723, Integra Biosciences, Figure S2a; Video S2).

The tips were held 300 µm above the well bottom, which makes the same routine applicable when hydrogel in the <200 µm thickness range are present on the bottom of the wells. Each cycle dispensed fresh MES buffer from a reservoir, mixed it with the well content three times by pipetting, and aspirated all the possible volume above the 300 µm threshold to mimic vacuum aspiration on a safe range. The recovered liquid was transferred to a 96-well collection plate and the cycle was repeated six times, with no waiting time between cycles.

The collected fractions were visually clear by the sixth cycle (Figure S2b). For reference, a 1 µg mL^{-1} tartrazine solution in MES or PBS remains visibly tinted under the same inspection conditions, so this concentration provides an operational stop criterion for visual or machine-vision inspection. Six dispense, mix, and aspirate cycles therefore bring the soluble absorber below the visible-tint threshold when a residual volume on the well bottom is present, which places hydrogel development within the capability of standard tip-based liquid handling.

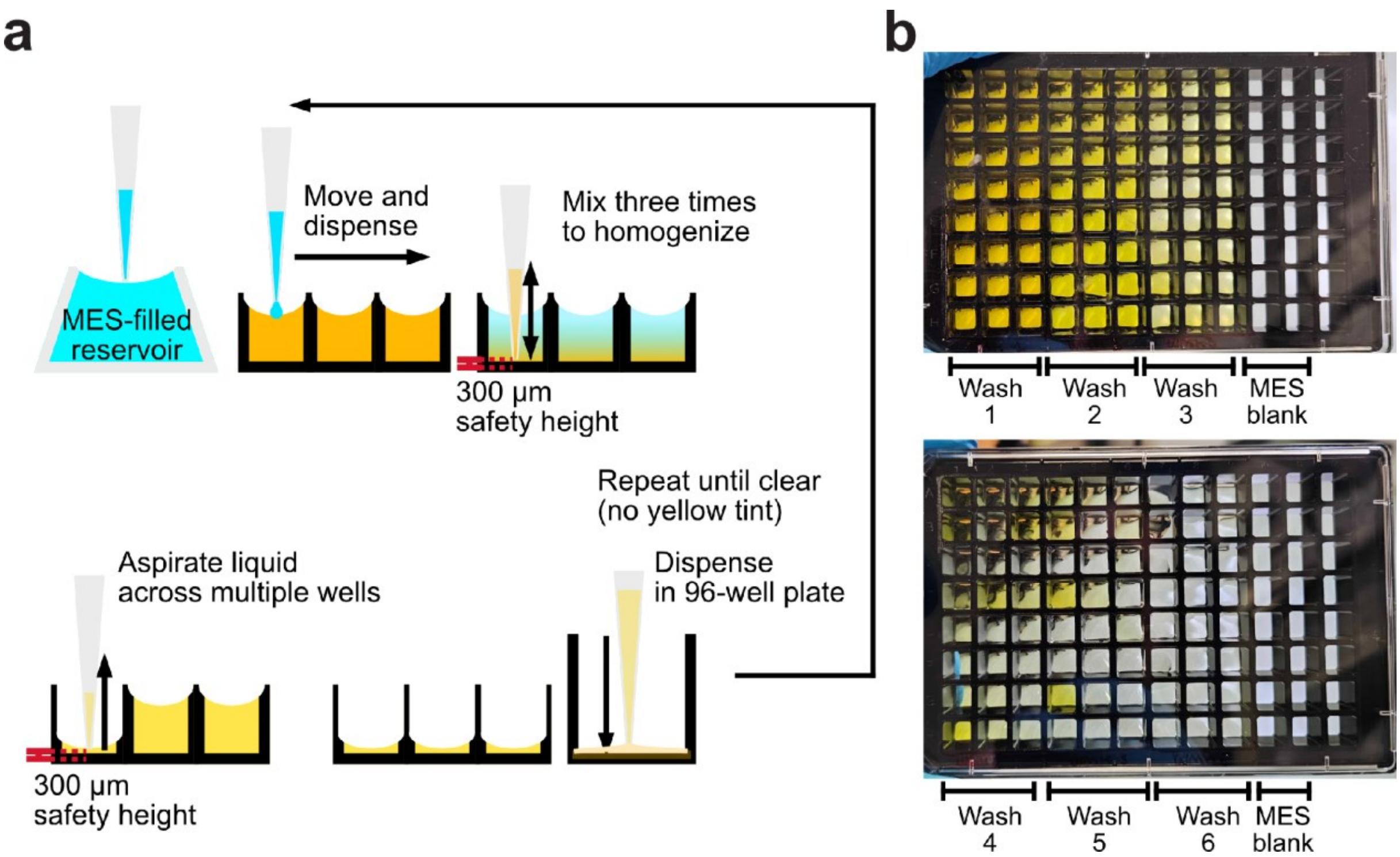

Figure S2. Automated liquid handling removes soluble tartrazine without bottom-contact aspiration. a) Schematic of one wash cycle. The pipetting robot draws fresh MES buffer from a reservoir, dispenses it into the tartrazine-containing wells, and mixes three times by pipetting with the tip held 300 µm above the well bottom. The liquid is aspirated at the same clearance, transferred to a 96-well collection plate, and replaced with fresh MES; the cycle repeats until the wells are clear. The 300 µm safety height keeps the tip away from a hydrogel of LigHTS thickness, so the routine transfers directly to gel-containing wells. b) Collection plates photographed after washes 1-3 (top) and 4-6 (bottom), each alongside MES-only blank columns. The yellow tartrazine signal fades progressively and is no longer visible after the sixth cycle. The endpoint shown here is the visual comparison against the MES-only blanks. The 1 µg mL^{-1} tartrazine reference that remains visibly tinted under the same inspection conditions defines the stop criterion (Note S2).

Note S3. Top-surface analysis pipeline (flat_gel_surface_analyzer.py): field-of-view identification, preprocessing, metrology definitions, quality control, and statistics

Confocal bead-layer top-surface analysis (flat_gel_surface_analyzer.py): confocal z-stacks of a fluorescent bead coating at the gel-medium interface were processed with a standalone, versioned Python pipeline (flat_gel_surface_analyzer.py; code and versioned outputs available via GitHub as stated in Data Availability). The purpose is to reconstruct a per-pixel top-surface height map and report (i) longer-wavelength within-well non-uniformity and (ii) shorter-wavelength residual dispersion using automation-compatible, robust metrics with explicit quality-control (QC) flags (Figure S3; Videos S1, S3, and S4).

Stacks are read from ND2 or TIFF as a 3D volume in (Z, Y, X) order. If additional axes are present (e.g., time/channel/position), the pipeline deterministically selects a single index per axis (default: index 0) to enforce reproducibility. The lateral pixel size (XY, µm px^{-1}) and axial sampling (Δz, µm) are obtained from metadata when available; otherwise they are provided as inputs. The user provides the relative axial position of the first frame of the stack (Z_0, µm), from glass-gel interface, so that the algorithm can use that information and identify the gel top surface to calculate the gel height (Figure S3d-g; Video S1).

Analysis is restricted to the well interior, or to the full field of view (FOV) when the acquired FOV is already fully contained within the well. When the FOV intersects the well boundary, a maximum-intensity projection is used to segment a conservative rounded-rectangle well interior (Figure S3a). Candidate masks derived from contrast enhancement and morphological processing are scored against a rounded-square well model using bead-signal coverage, alignment to strong edges/gradients, and penalties for masks abutting the image boundary; the best candidate is locally refined by small translations and scalings. To suppress rim/meniscus/wall-scatter artifacts while preserving a centered gel region, the selected ROI is then uniformly scaled inward (typically by ~10%); diagnostic overlays are exported for audit (Figure S3a; Videos S3 and S4). When the FOV is fully contained within the well (e.g., when imaging is purposefully centered and cropped), the full image is used as the ROI without segmentation.

Within the ROI, the bead-layer axial position is estimated independently for each (x,y) pixel. First, an integer-slice peak is located by taking the z-index of the maximum intensity at each pixel. Second, for non-truncated acquisitions, sub-slice refinement is performed by fitting a local parabola to the peak and its immediate neighbors, constraining the refinement to ±0.5 slices to reduce discretization error (Figure S3b). Third, pixels are QC-gated to prevent

propagation of unreliable peaks: pixels are excluded if the peak lies on the first/last z-slice (boundary peak) or if a conservative peak-quality statistic (computed from the same three points used for interpolation) falls below a within-ROI threshold; excluded pixels are set to NaN. Passing pixels are converted to physical height using the acquisition z step and origin stored in the image metadata. The fractions of valid pixels within the full ROI and within a central "core ROI" (an eroded subset used for robust plane fitting) are recorded as primary QC indicators and are used downstream to flag problematic stacks.

To avoid biased inference from incomplete axial sampling, the pipeline explicitly detects z-truncation before reporting thickness/uniformity metrics. A truncation condition is triggered when a substantial fraction of ROI pixels peak on a boundary slice and that boundary slice carries high-intensity signal relative to the maximum-intensity projection (i.e., the interface signal is likely beyond the acquired z range). When truncation is detected, the workflow switches to a conservative truncation mode: it reverts to integer peak locations, accepts only interior peaks passing a high-intensity criterion, assigns sentinel "beyond-range" values otherwise, and withholds thickness/uniformity outputs (reported as NaN) while exporting only QC/provenance fields (e.g., a truncation-mode flag, valid-pixel fractions, and pixel counts). This makes truncation detectable and auditable without allowing it to silently compress reported dispersion.

For non-truncated stacks, residual mounting/stage tilt within the FOV is estimated by least-squares fitting a plane $z = a{\cdot}x + b{\cdot}y + c$ over the core ROI (x,y in μm and centered). Tilt correction subtracts only the slope terms ($a{\cdot}x + b{\cdot}y$) while preserving the offset c so that maps remain anchored in the acquisition reference frame. Longer-wavelength within-well non-uniformity is quantified from a Gaussian low-pass filtered version of the tilt-corrected map, where the cutoff wavelength is fixed in physical units and implemented as a corresponding Gaussian σ in pixels. Summary statistics are computed on a conservative inlier subset defined by percentile trimming of the low-pass filtered heights within the core ROI to reduce sensitivity to sparse artifacts (e.g., bead aggregates or segmentation edge leakage) without assuming normality. The reported longer-scale metrics are the inlier mean surface height and the inlier standard deviation (capturing long-wavelength non-uniformity after tilt removal and trimming). Plane-fit diagnostics (a,b,c and goodness-of-fit) and a derived tilt magnitude are exported to support auditability.

Shorter-wavelength residual dispersion is quantified by an S-L band-pass construction on the tilt-corrected map. A short-scale Gaussian smoothing ("S-filter", cutoff λ_s) is applied first, with λ_s chosen deterministically from imaging scale and an effective bead/feature-size heuristic with

a minimum bound in pixel units to prevent under-smoothing. Then a L-filter (cutoff λc) is applied to the S-smoothed map to extract its long component, and the band-pass residual is defined as (S-smoothed) − (L-of-S-smoothed), isolating intermediate spatial scales. Outliers are rejected within the core ROI using a fixed MAD-based rule, and the residual dispersion is reported as the standard deviation over robust inliers (BandpassStd; an RMS-like dispersion metric rather than a thickness metric). The effective λs and bead-size heuristic values are exported for traceability.

For each analyzed folder, the pipeline writes a per-stack summary table (CSV) reporting the primary height metrics (mean and standard deviation of the low-pass and band-pass filtered height maps), truncation/QC fields (valid-pixel fractions, pixel counts, truncation mode), plane-fit/tilt diagnostics, and acquisition/analysis metadata (pixel size, z step, stage coordinates, filter cutoffs and other fixed parameters) along with a run identifier. A separate run-level metadata file records software provenance (script version/hash, platform and package versions, and fixed parameters). Optional diagnostic PNGs are exported per stack to enable rapid visual audit (maximum-intensity projection with ROI overlay; reconstructed height map; low-pass residual; band-pass residual), supporting the qualitative checks shown in Figure S3 and Videos S3 and S4.

Plate-level z normalization for flat-gel thickness metrology (tilt/warp across wells): across a multiwell plate, the microscope stage z (and thus the apparent absolute height reference) varies systematically with lateral position due to plate tilt and mild warp. For plate-wide thickness comparisons, we therefore apply a plate-level z normalization that is distinct from the within-FOV plane subtraction used for metric extraction. Using reference wells/regions with reliable stage coordinates, we fit a smooth surface $\hat{z}(x,y)$ to predict the expected stage height at any well position. When tilt dominates over the imaged region, a planar model is sufficient; when mild curvature/warp is evident, a second-order (full quadratic) surface is used. This correction is applied as an additive offset that maps each well's local stack reference onto a common plate-level z frame, ensuring that thickness trends reflect polymerization physics rather than systematic stage-height drift across the plate (Figure S3f-g). For transparency, the uncorrected and corrected counterparts of the main-text thickness maps are shown in Figure S3d-e, and representative plate-wide thickness reconstructions are provided in Videos S1-S3.

All plate-level fit diagnostics and per-ROI offsets are exported alongside the downstream thickness/height summaries to support auditability and reproduction (see the repository scripts and run metadata for the exact parameterization used in each analysis run).

For each ROI/well, the acquisition metadata provides the physical z coordinate of the first slice and the z step size, and stacks may be defined relative to an internal reference plane (e.g., autofocus plane or nominal center slice). To place all stacks into a common plate-level z frame, we apply a constant z translation per ROI equal to the difference between the predicted plate surface height $\hat{z}(x,y)$ at that ROI and the ROI's internal reference height. After this shift, the corrected z coordinates for every slice in the stack are expressed in the same global z frame, enabling inter-well comparison of thickness-derived quantities without systematic bias from plate tilt or differing stack origins.

Per-ROI z offset for z-stack comparability: using reference wells/regions with reliable stage coordinates, we fit a smooth surface $\hat{z}(x,y)$ that predicts the expected stage height at any well position. When tilt dominates, a planar model is sufficient; when mild curvature is evident, a full quadratic surface is used. In the quadratic case, the model takes the form $\hat{z}(x,y) = \beta_0 + \beta_x x + \beta_y y + \beta_{xx} x^2 + \beta_{yy} y^2 + \beta_{xy} x y$, fitted by least squares. The fitted surface provides an expected plate-level z reference even for wells with missing or unreliable stage z readings.

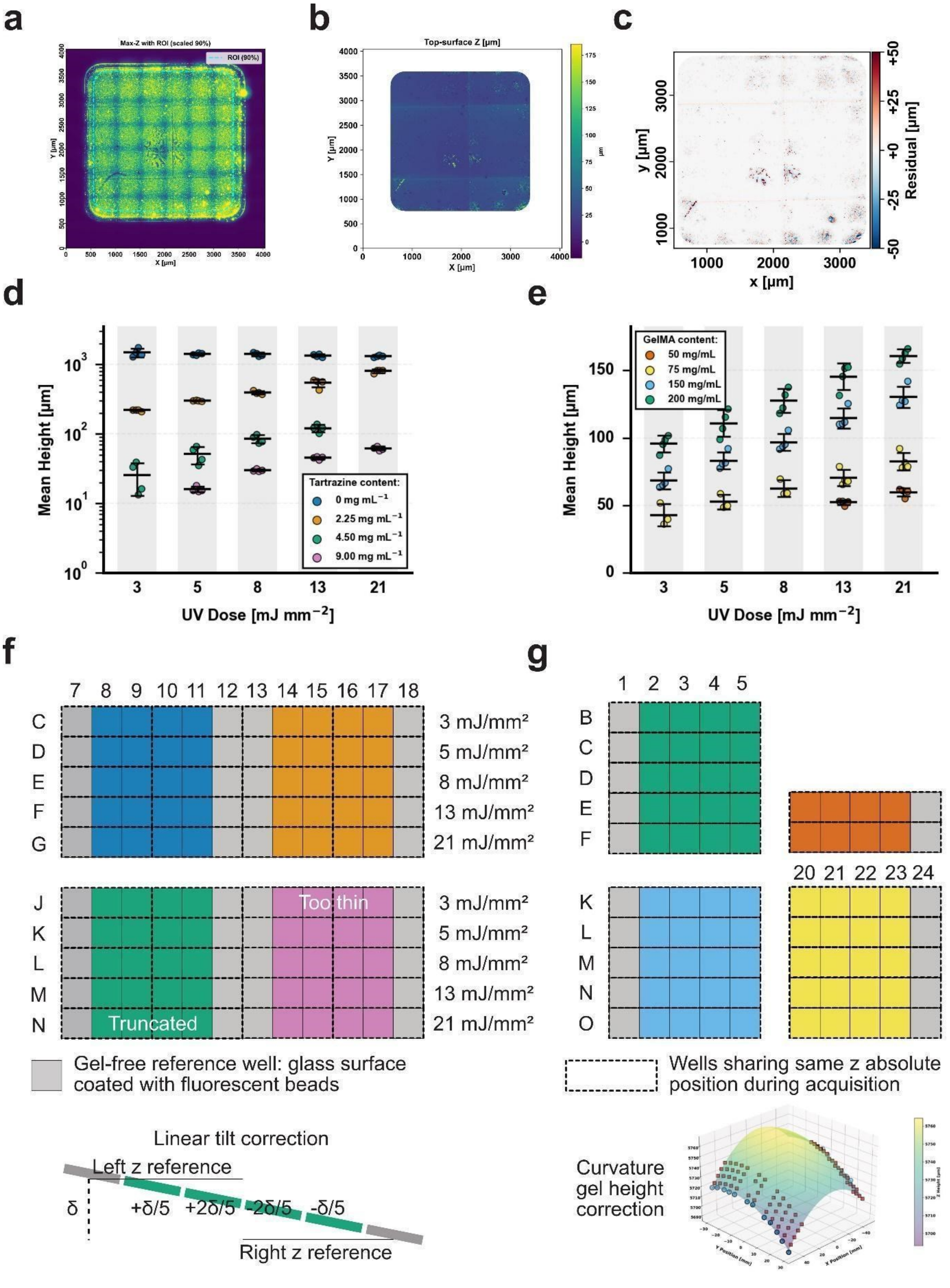

Figure S3. Confocal bead-layer top-surface reconstruction and z normalization workflow for flat-gel thickness metrology. a) Maximum-intensity projection (Max-Z) of a confocal z-stack acquired on fluorescent-bead-coated (“bead-layer”) flat GelMA gels, used for automated segmentation of the well interior. The analysis region-of-interest (ROI) is conservatively scaled

inward to 90% of the segmented footprint to suppress rim/edge/meniscus artifacts (axes in µm). b) Reconstructed per-pixel top-surface map obtained by locating the axial intensity maximum of the bead layer at each pixel and refining the peak position with sub-slice interpolation; the resulting map defines the absolute gel top surface prior to detrending (axes in µm; color scale as shown). c) Residual height map (Residual, µm) after least-squares plane fitting and subtraction, highlighting within-well non-uniformities independently of global tilt (axes in µm; color scale as shown). d) Raw (non-tilt-corrected) mean gel thickness versus UV dose for varying tartrazine content (legend). Each point represents one independent well (n = 3-4 wells/condition; one measurement per well; no time averaging); black bars denote mean ± SD. This panel is the uncorrected counterpart of main-text Figure 2g and is shown here to visualize the magnitude of plate tilt prior to correction. e) Raw (non-tilt/warp-corrected) mean gel thickness versus UV dose for varying GelMA content (legend), quantified as in (d) from independent wells (n = 3-4 wells/condition; mean ± SD). This panel is the uncorrected counterpart of main-text Figure 2h and is shown here to visualize the impact of plate-scale z offsets prior to normalization. f) Linear tilt correction strategy used to convert (d) to main-text Figure 2g. Schematic well-map indicating gel-free, bead-coated reference wells (gray) used to measure the left-right z offset (δ) within an acquisition block. The measured δ is distributed across intermediate columns to compute a per-well z correction (illustrated as ±δ/5 steps), aligning all wells to a common absolute z reference before extracting mean thickness. Wells flagged “Truncated” or “Too thin” illustrate quality-control exclusions. g) Plate curvature/warp correction and acquisition grouping used to convert (e) to main-text Figure 2h. Wells are acquired in groups sharing the same nominal stage z (dashed outlines), then normalized into a common coordinate frame using gel-free reference wells to fit a smooth 2D surface (including second-order terms) capturing plate-scale tilt and mild curvature. The resulting per-well z offsets are applied prior to thickness extraction to ensure consistent absolute comparisons across the plate. Microscopy acquisition settings (illumination power, exposure time, binning, and gain) are reported in the Main Text Experimental Section.

Note S4. Spatially resolved nanoindentation of flat LigHTS hydrogels

The mechanical characterization summarized in Figure 2i was acquired as a spatial map so that heterogeneity within a gel could be assessed together with the dependence on polymer content. Sample displacement was reconstructed from piezo displacement minus cantilever deflection. A straight-line baseline in time was fitted to the pre-contact stretch of each curve and removed, since the baseline is an error on the measured deflection, the same correction was propagated to the displacement so that force and displacement remain internally consistent. The contact point and modulus were obtained in two passes over the linearized Hertz plot ($s = s_0 + \beta F^{2/3}$): a seed regression over 20–100% of peak force anchored a fixed indentation window, and a second regression within that window returned the reported contact point s_0 and the contact modulus E* from the slope, using a rigid spherical Hertz model, $F \propto (s - s_0)^{3/2}$. The window for indentation was fixed at 0.5–3.5 µm on every curve: the lower edge excludes the contact-sensitive shallow region, and the 3.5 µm cap (13-14% of the probes radii, within the instrument 16% guidance) is the deepest window every retained curve fills. The figures report the contact modulus E* as measured, since the Poisson ratio of these gels was not characterized. A curve was accepted when its fit converged to a finite modulus within 0.1–100 kPa, reached the 3.5 µm analysis depth, had a normalized RMS fit residual of at most 8%, and a peak-force to baseline-noise ratio of at least 20; it additionally had to pass mechanical checks on common instrument failure modes. Based on those criteria, 395 of 432 curves passed (91.4%). Within each map, the mechanically identified contact surface was approximately planar after removing a per-gel least-squares tilt plane, with 95% of residual points within 2 µm of the detrended median (RMS ≈ 1 µm; Figure S4).

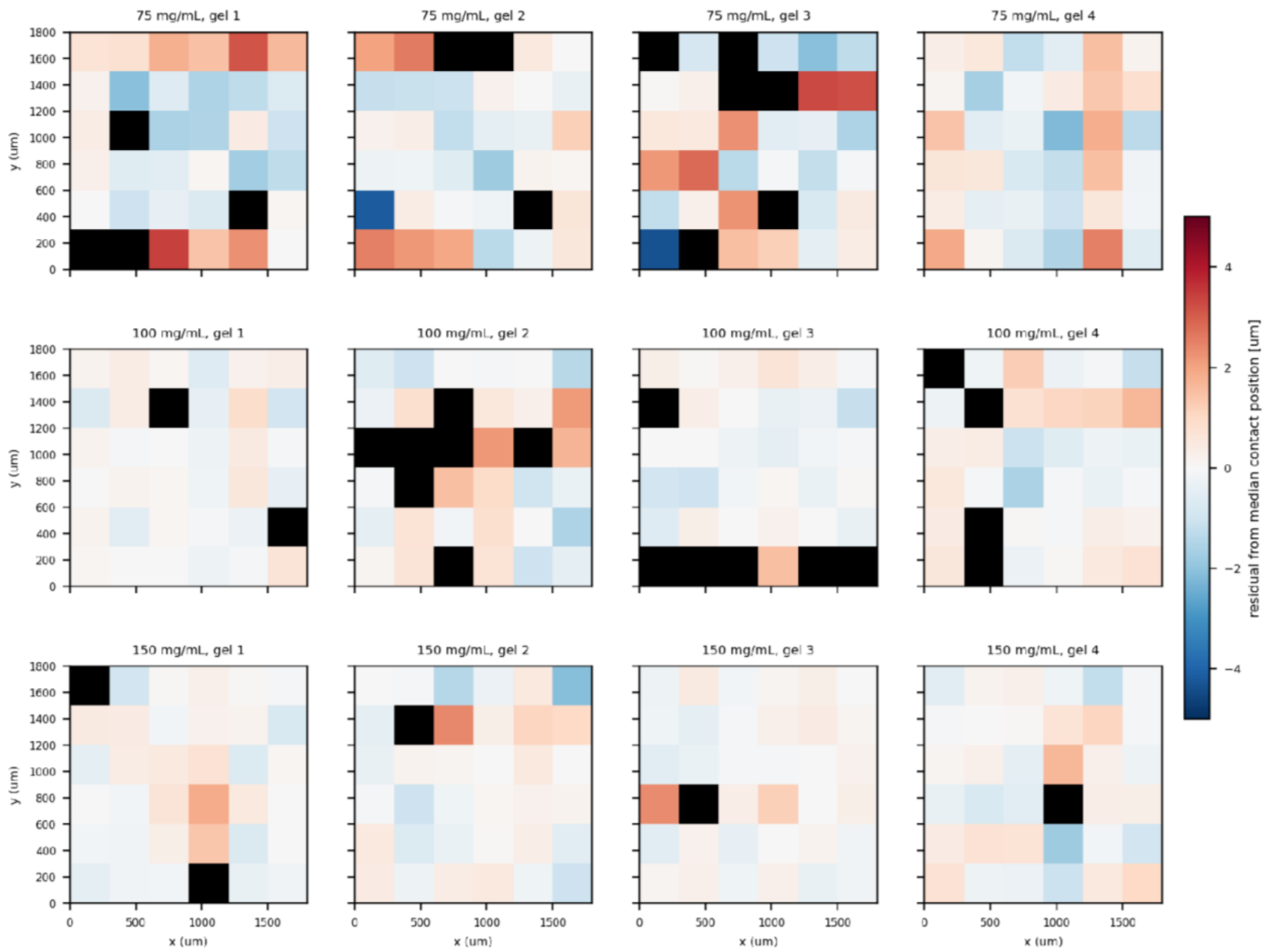

Figure S4. The mechanically identified surface is planar across each indentation map. Detrended contact-coordinate residuals for the twelve gels of the spatial nanoindentation dataset, four independently prepared gels at each of 75, 100, and 150 mg mL^{-1} GelMA, plotted at their physical positions on the nominal 6 × 6 grid. For each gel a plane was fitted to the contact coordinates s_0 across the map and subtracted; the color scale shows the remaining residual. Black cells indicate curve-quality-control failure or absence of a valid Hertz fit.

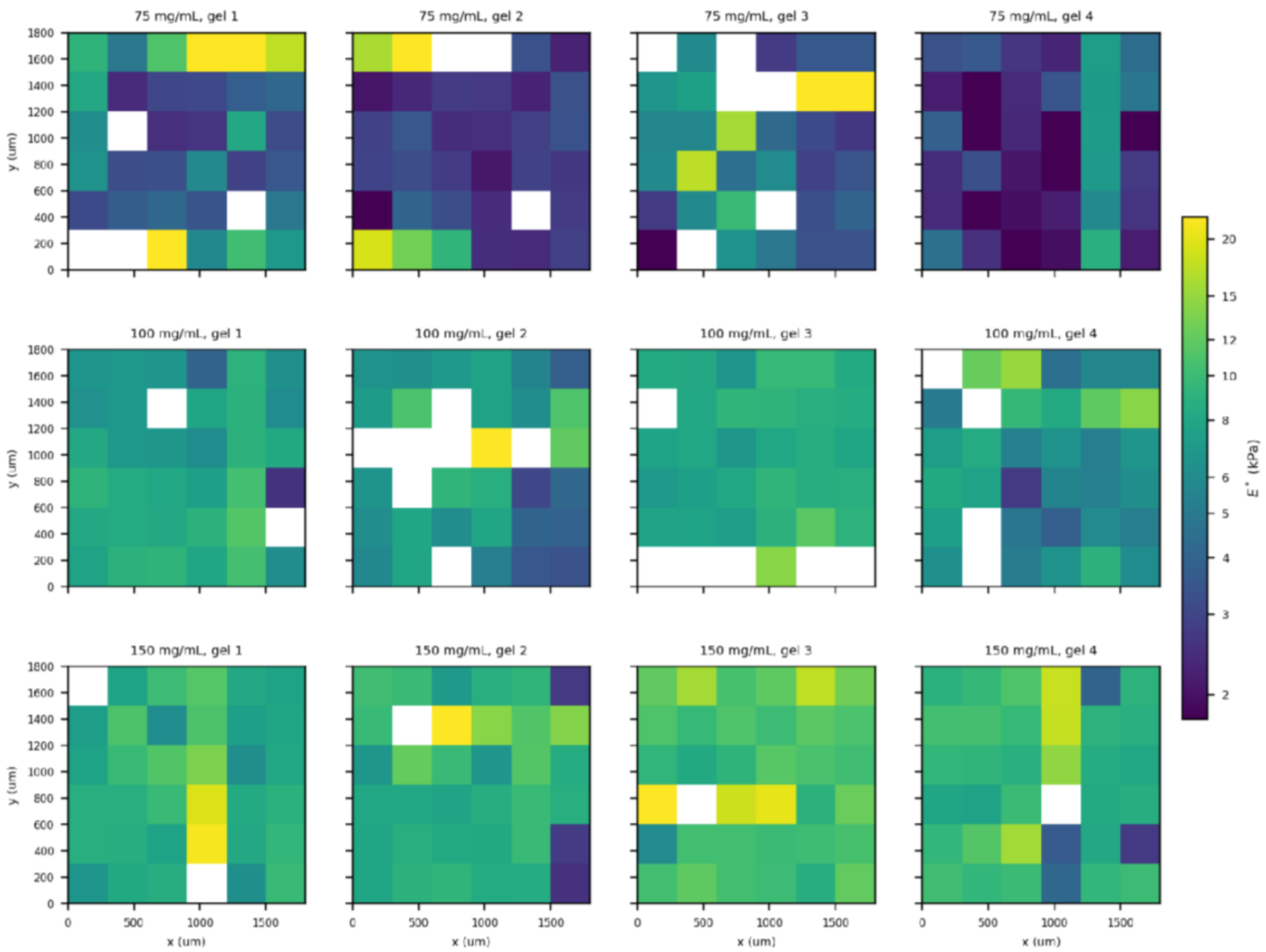

Figure S5. Local modulus varies within a gel while the ranking with polymer content is preserved. Reduced modulus E* at each position of the nominal 6 × 6 indentation grid, for four independently prepared gels at each of 75, 100, and 150 mg mL^{-1} GelMA; these are the maps summarized in Figure 2i. The shared color scale reports E* in kPa. Median gel-level values were 3.7, 7.3, and 9.1 kPa, and robust coefficients of variation within a map were approximately 20-50%, 10-45%, and 15-25% for 75, 100, and 150 mg mL^{-1} respectively, quoted to the nearest 5 percentage points. White cells carry no reportable value.

Note S5. Stability during refrigerated storage and reproducibility between batches

To assess storage stability, we characterized GelMA hydrogels right after patterning and after one month of hydrated storage and compared two results (Figure S6). Gels were cast, exposed, developed, and post-cured as described in the Experimental Section. For these tests, a stock 100 mg mL^{-1} GelMA solution (lot A294, product D16110025360, CellInk) was mixed with tartrazine, LAP, and glycerol in PBS to reach 75 mg mL^{-1} to prepare a 384-well plate and reproduced the DMD-based experiment presented in Figure 2g for characterizing gel thickness stability using the described confocal microscopy pipeline (Figure S6a). We instead used lyophilized GelMA (lot A326, product 5208, CellInk) to prepare the samples in 35-mm glass-

bottom wells for mechanical characterization (Figure S6b), since the nanoindenter probes were too large to perform a matrix scan within 384-well plates. After the first characterization, the samples were kept in PBS (pH 7.4) at 4 °C, protected from light, and re-measured after one month.

To assess batch-to-batch variability, we compared the hydrogel thickness described above with the data shown in Figure 2g (lot 9907, processed at similar room temperature, Figure S6c). We also evaluated batch-to-batch variability comparing samples produced with different batches (lot 9617, patterned at 26-30 °C, Figure S6d).

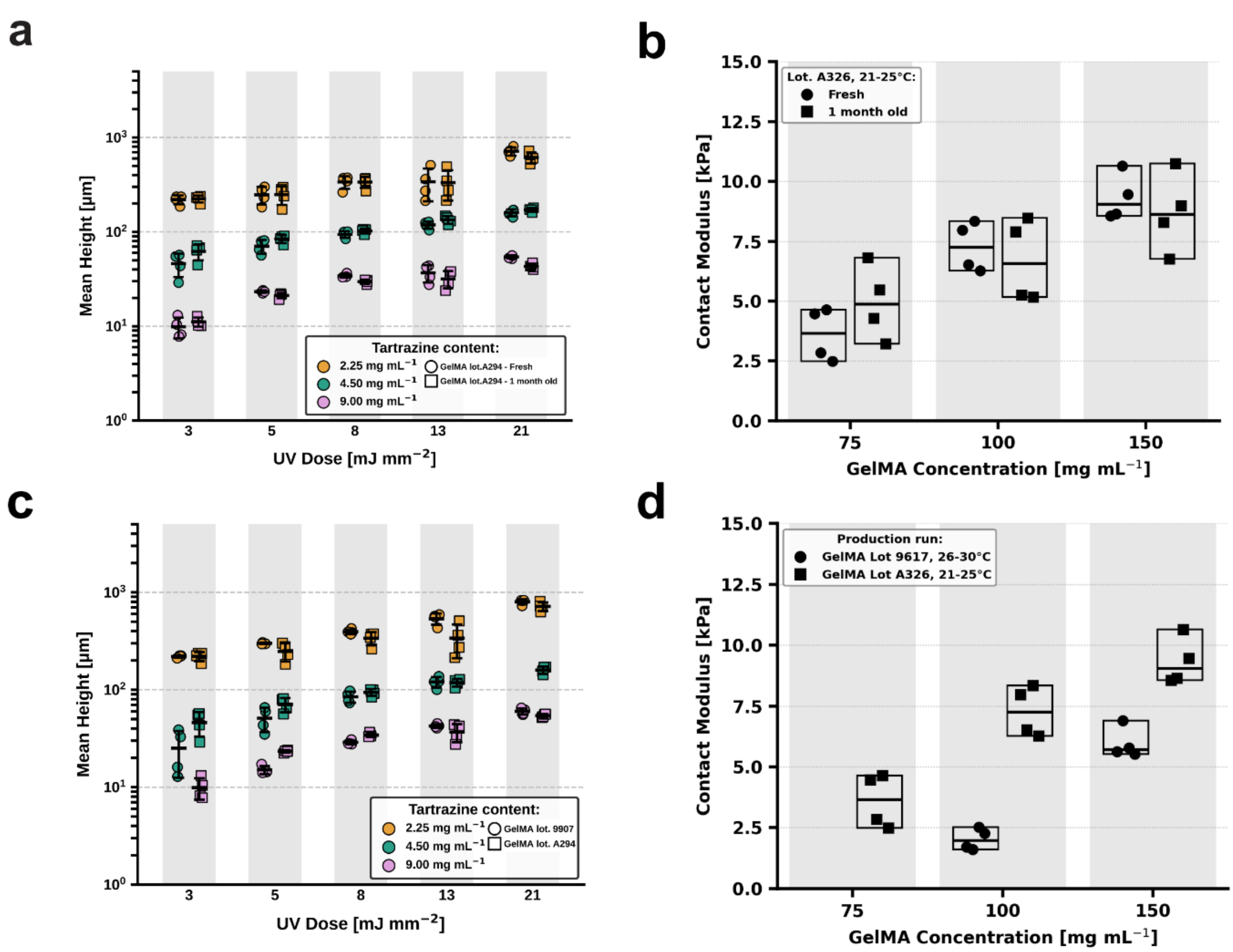

Figure S6. Long-term cold-storage stability and batch-to-batch variability of LigHTS-patterned GelMA hydrogels. a) Mean hydrogel height versus UV dose at three tartrazine concentrations, measured on the same batch immediately after fabrication (circles) and after one month in PBS at 4 °C (squares). Fifteen conditions, four wells each, showed the same trends over time. b) Long-term cold-storage stability of the mechanical properties of GelMA hydrogels prepared from lyophilized GelMA lot A326 photopatterned at 21-25 °C room temperature. Circles and squares indicate contact modulus values extracted, respectively, right after gel fabrication and after one month storage. c) Mean hydrogel height versus UV dose for two

independently prepared batches (circles, lot 9907; squares, lot A294) photopatterned at 21-25 °C room temperature. Across the shared conditions, the median difference was 14%, more prominent on the low dose side. d) Effect of lot and process temperature variability on LigHTS-patterned GelMA hydrogels. 100 and 150 mg mL^{-1} GelMA solutions from lot 9167 illuminated at 26-30 °C (circles) produced softer gels than the ones prepared with lot A326 and illuminated at 21-25 °C (squares), and only 75 mg mL^{-1} solutions patterned at the lower temperature survived development. In a and c, each point is one well and black bars denote mean ± SD. In b and d, each point is one gel, the box spans the interquartile range, and the horizontal line marks the median.

Note S6. Soluble tartrazine released after complete fabrication

To quantify the residual absorber after development, we collected sequential extracts from fully fabricated hydrogels of a mask-aligner-patterned 384-well plate and measured the optical absorbance using a plate reader (POLARstar Omega, BMG LABTECH). After UV flooding the wells were filled with PBS and incubated overnight at 37 °C. The solutions were then extracted after 12 hours (S1), then the wells were refilled with fresh PBS, and a second extract (S2) was collected after a further 12 h.

The extracts from the individual well for each collection timepoint were pooled together and cast as 500 µL aliquots in a 96-wellplate and optically characterized at 425 nm together with PBS blanks (n = 28) and tartrazine standards at 100, 250, and 500 ng mL^{-1} (n = 10 wells each; Figure S7a). The detection limit was defined as the blank mean plus three standard deviations, which gives 0.037 optical density (OD) for this plate (blank mean 0.025, SD 0.004, n = 28). Because the assay reports absorbance rather than identifying the absorbing species, values are expressed as tartrazine equivalents.

The one-sided 95% confidence interval, 0.034 OD for S1 (n = 10) and 0.029 OD for S2 (n = 9), against a detection limit of 0.037 OD, indicates that equivalent tartrazine concentrations for S1 and S2 were below the detection limit, which is estimated to correspond to 250 ng mL^{-1} standard. One well in the S2 group returned an isolated reading of 0.242 OD, roughly ten-fold above every other sample well and we attribute it to an obstruction of the optical path in that well and excluded it from the comparison.

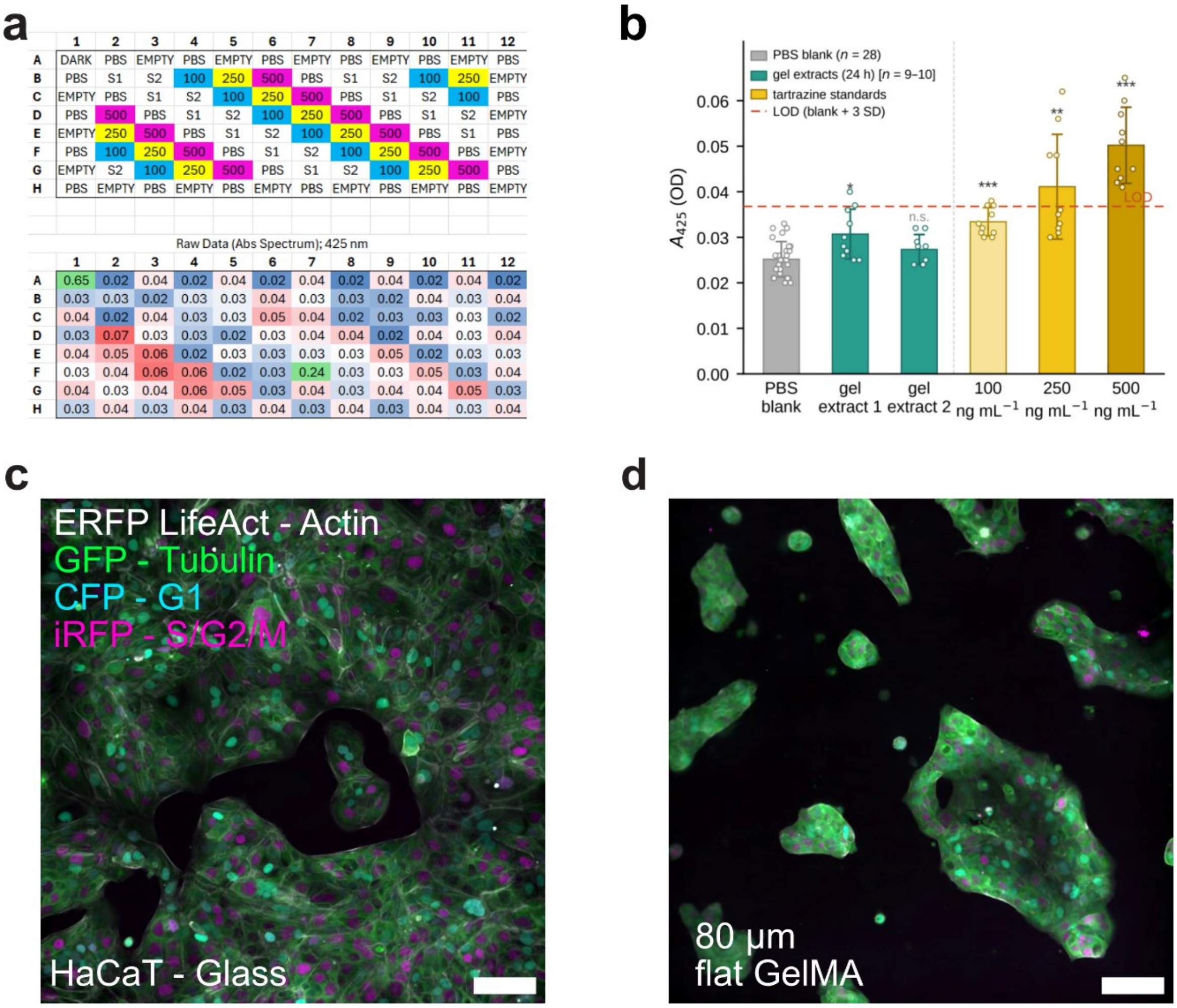

Figure S7. Residual tartrazine after fabrication lies below the detection limit, and LigHTS hydrogels support colony-forming keratinocytes. a) 96-well layout used for the extract assay (top) and the corresponding raw absorbance readings at 425 nm (bottom). PBS blanks, the sequential gel extracts S1 and S2, and tartrazine standards at 100, 250, and 500 ng mL^{-1} were interleaved across the plate to guard against position-dependent readout bias. b) A425 of the PBS blanks ($n = 28$), the two sequential gel extracts ($n = 9$-10 each), and the tartrazine standards. Bars show mean ± SD with individual wells overlaid, and the dashed line marks the detection limit defined as the blank mean plus three standard deviations (0.037 OD). Significance symbols refer to two-sided Mann-Whitney U comparisons with the PBS blank, Holm-adjusted (n.s., not significant; * $p < 0.05$; ** $p < 0.01$; *** $p < 0.001$). Both extracts fall below the detection limit and below the 250 ng mL^{-1} standard. Extract 1 is still distinguishable from buffer, consistent with a trace below the reporting threshold (Note S6). c,d) Four-channel confocal images of HaCaT human keratinocytes cultured on fibronectin-coated glass (c) and on a fibronectin-coated 75 mg mL^{-1} GelMA film approximately 80 µm thick (d). F-actin (ERFP-LifeAct) is shown in gray, tubulin (GFP) in green, and the FUCCI reporters for G1 (CFP) and S/G2/M (iRFP) in cyan and magenta. Colonies formed on both substrates; on glass they merged

into a near-confluent sheet, whereas on the hydrogel they remained discrete with visibly smaller individual cell areas. Scale bars: 100 µm.

Note S7. Cell area analysis pipeline and statistical inference

Overview and data structure: this analysis quantifies substrate-dependent differences in projected cell spreading area for HT1080 cells cultured on glass versus thin GelMA films (nominally 75 mg mL^{-1} and 150 mg mL^{-1}; Figure 3e). The design is hierarchical: cells are nested within FOVs, and FOVs are nested within independent experiments (N = 3). Each experiment contains three FOV technical replicates per condition (balanced 3x3 layout), and each FOV contains tens-to-hundreds of segmented cells (per-condition totals on the order of several hundred cells across experiments). For transparency and reproducibility, the workflow saves intermediate outputs (QC images and masks) and exports a single merged "long-format" table for statistics.

Image inputs and preprocessing: images are stored as ND2 files generated by NIS-Elements and organized in experiment-specific folders. The segmentation script is configured to process .nd2 documents suffixed with _filtered, consistent with exporting denoised images from NIS-Elements. The analysis uses the fluorescence channels carrying actin and tubulin signals (additional channels, if present, are ignored for segmentation).

Segmentation and mask QC (segment_cellpose_sam_masks.py): segmentation and mask quality control were performed with a standalone Python script (segment_cellpose_sam_masks.py; code and versioned outputs available via GitHub as stated in Data Availability). Whole-cell masks are obtained with Cellpose-SAM via the CellposeModel interface. The model is run on the two-channel tensor containing actin and tubulin, which improves robustness compared with single-channel segmentation when cells exhibit heterogeneous cytoskeletal contrast. The script auto-detects GPU availability (CUDA) but runs identically on CPU (slower). Segmentation parameters are held fixed across conditions (e.g., default model normalization settings) to avoid condition-dependent tuning. For each image, the pipeline writes (i) a QC panel comparing the fluorescence composite to the label map for rapid visual verification, (ii) a label TIFF (integer mask image) for traceable downstream measurements, and (iii) a per-image CSV containing per-cell features.

Area measurement in physical units: single-cell projected area is extracted from the label image using standard connected-component region-property extraction (scikit-image). Area is

reported in µm² by applying the physical pixel sizes (X and Y) read from ND2 metadata (via AICSImageIO). The per-cell output includes at minimum the label identifier and area, and is written per image to a CSV stored alongside the QC outputs.

Dataset assembly (merge_csvs.py): per-image CSVs are concatenated across experiments and conditions into a single dataset via a Python script (merge_csvs.py; code and versioned outputs available via GitHub as stated in Data Availability). During merging, each cell is annotated with its condition (Glass, 150 mg mL^{-1} GelMA, or 75 mg mL^{-1} GelMA), the experiment identifier (block), and the field-of-view identifier (technical replicate). This merged file is the sole input to the statistical script and figure generation.

Statistical inference and plotting: all statistical inference is performed at the field-of-view (FOV) level to avoid pseudoreplication arising from treating cells within the same FOV or experiment as independent biological replicates. Figure 3e displays per-cell data points for visualization ease. The analysis proceeds through five steps. First, a FOV-level summary is computed: for each experiment x condition x FOV combination, the median of $\log_{10}$(area) across cells is calculated, where the log transform stabilizes variance and enables fold-change interpretation. Second, a blocked omnibus test assesses whether any group effect exists across the three conditions by permuting group labels within each experiment block (preserving the balanced three-FOV-per-condition structure); the test statistic is a one-way F statistic computed after within-block centering, with the p-value estimated from 50000 permutations using a seeded random number generator. Third, blocked pairwise tests are conducted for each pair of conditions using two-sided blocked permutation tests on the same FOV-level summaries, with within-block label shuffling restricted to the two-group subset. For the balanced three-versus-three design used here that enumeration contains $20^3 = 8000$ arrangements, and the label-reversed arrangement always ties the observed one, so the smallest attainable two-sided p-value is 2.5×10^{-4} before adjustment and 7.5×10^{-4} after Holm. The Figure 3e contrasts sit at that floor, so they are reported as $p < 0.001$ and cannot be reported lower however large the effect. Fourth, pairwise p-values are adjusted using Holm step-down control of the family-wise error rate (FWER) at $\alpha = 0.05$. Fifth, per-experiment contrasts on the mean $\log_{10}$(FOV summary) and corresponding fold changes ($10^{\Delta\log10}$) are reported as descriptive effect summaries. All outputs (FOV summary table, omnibus test report, pairwise permutation table, effect summary) are exported alongside the plotted figure for auditability.

Note S8. HaCaT human keratinocytes cell culture, seeding, and microscopy

To test a human cell type with a different organization than individually migrating HT1080 cells, we cultured HaCaT keratinocytes on LigHTS hydrogels. HaCaT human keratinocytes genetically engineered with fluorescent reporters were maintained in phenol red-free DMEM/F-12 supplemented with 10% v/v fetal bovine serum and 1% v/v penicillin-streptomycin, and passaged before reaching full confluence. Cells were seeded at 4×10^4 cells mL^{-1} on flat 75 mg mL^{-1} GelMA films approximately 80 µm thick and on glass controls, both coated beforehand with human plasma fibronectin (40 µg mL^{-1} in PBS, 1 h at room temperature). Four-channel confocal images were acquired with the 20x/0.75 NA objective.

Note S9. Groove analysis pipeline (groove_analyzer.py): field-of-view strategy, preprocessing, metrology definitions, quality control, and statistics

Confocal bead-layer topography analysis (groove_analyzer.py): Confocal z-stacks of a fluorescent bead coating at the gel-medium interface to determine its surface topography were processed with a standalone, versioned Python pipeline (groove_analyzer.py; code and versioned outputs available via GitHub as stated in Data Availability). The analysis assumes a single dominant bead-layer interface signal per (x,y) pixel and a groove pattern that is the primary periodic feature within the analyzed FOV.

PRIMO DMD datasets (Figure 4) were acquired as a 2x2 mosaic at 20x to cover the printing region. Mosaic stitching can introduce non-physical single-digit-µm steps at tile seams that would spuriously inflate groove depth and distort Fourier-based orientation estimates. Therefore, for each condition we manually cropped ~1000 µm sub-FOVs from within a single tile interior, explicitly excluding seams and other stitching defects.

For plate-wide photomask datasets (Figures 5-6), stacks were processed at scale. A fixed-size sub-FOV (~1000 µm) was automatically center-cropped from each RAW acquisition to (i) standardize metrology footprint across wells and (ii) minimize edge effects (e.g., meniscus/wall scattering) without subjective selection. These choices ensure that comparisons across fabrication modes reflect polymerization physics rather than imaging artifacts or seam discontinuities.

Denoising and the RAW-vs-denoised equivalence control: for PRIMO datasets, z-stacks were exported after applying the NIS-Elements denoiser to improve the robustness of per-pixel axial peak localization under lower SNR conditions. For plate-wide datasets (Figure 5-6), analysis

was performed on RAW stacks to preserve a fully automated pipeline and avoid dependence on proprietary preprocessing.

To verify that preprocessing did not bias conclusions, we performed a paired control in which the same stacks were analyzed as RAW and after NIS denoising using identical downstream settings. Agreement was assessed per stack for pitch, depth, and height (paired differences and Bland-Altman-style error relative to the reported type-A/type-B uncertainty terms). Across tested conditions, differences were negligible relative to uncertainty and did not alter group-level statistics. This control justifies using denoised PRIMO exports for robustness while retaining RAW-only processing for plate-scale automation.

Hydrogel surface reconstruction: for each (x,y) pixel, the axial position of maximum bead-layer intensity is identified and refined via a local quadratic fit to obtain sub-slice precision. The refined axial index is converted to µm using the acquisition z-step. On grooved datasets, no manual tilt adjustment was performed on any dataset. No plate-level curvature correction (e.g., fitting a well/plate warpage model across positions) was applied. When detrending is used, it is limited to an automated within-FOV best-fit plane removal to suppress residual stage/sample tilt so that groove amplitude metrics reflect periodic relief, not mounting angle.

Groove angle and a coarse pitch are estimated from the dominant component in the 2D Fourier spectrum of the detrended map. The map is rotated to align grooves with the analysis axis and then center-cropped to avoid rotation-edge artifacts. Pitch is defined as crest-to-crest spacing along the groove periodic axis. It is computed from band-limited line profiles using peak detection constrained by the FFT-derived pitch estimate; the reported pitch is the mean of successive crest-to-crest distances. Within-FOV spread is estimated from row-wise measurements with robust outlier rejection to suppress occasional peak mis-detections.

After peak identification, groove depth is defined per period as crest height minus the mean height of the two adjacent valleys, computed on baseline-corrected profiles and then averaged across valid periods within the FOV. Peak and valley identification is also used to report bulk film height independent of periodic relief. In fact, height/thickness is estimated by fitting a plane from the valley-height distribution (median and spread).

Assessment and correction of pitch-dependent systematic depth bias in the analysis pipeline: confocal reconstruction of a fluorescent bead layer can systematically underestimate groove depth due to axial sampling, PSF blur, and the smoothing required for stable peak/valley localization. To compensate, the workflow applies a pitch-dependent multiplicative correction derived from synthetic forward-model validation (sinusoidal grooves rendered at matched sampling/noise and processed through the same pipeline). The correction factor is interpolated

based on measured pitch and applied consistently to reported mean depth and associated uncertainty terms.

QC and exclusion rules: to prevent silent failure modes from contaminating plate-scale summaries, several quality-control gates are applied. First, stacks with insufficient valid peak-localization pixels (falling below a fixed valid-fraction threshold) are flagged and excluded to ensure interface detectability. Second, fields of view lacking a clear dominant periodic component (i.e., a weak or ambiguous FFT peak) are flagged as failing the periodicity check. Third, profiles yielding too few valid periods or exhibiting inconsistent peak spacing relative to the FFT-derived pitch are excluded from depth and pitch aggregation. Finally, per-stack diagnostic overlays (maximum-intensity projection with crop box, height map, aligned profiles, and peak/valley calls) are exported to support visual audit of every analyzed field of view.

Statistics reporting for groove metrics: for the large-n plate-wide datasets (Figures 5-6; $N > 100$ wells per group), group comparisons were performed in GraphPad Prism using two-sided tests at $\alpha = 0.05$; distributional assumptions were verified and nonparametric tests applied when warranted, with full test names, multiple-comparison corrections, and effect sizes reported in the main text statistics section. For the smaller-n PRIMO datasets (Figures 2-4; N = 3-4 per condition), inference relied on the dedicated Python-based statistical workflow used elsewhere in this study for small sample sizes, employing exact permutation logic and reporting two-sided p-values with Holm step-down correction for multiple comparisons as appropriate.

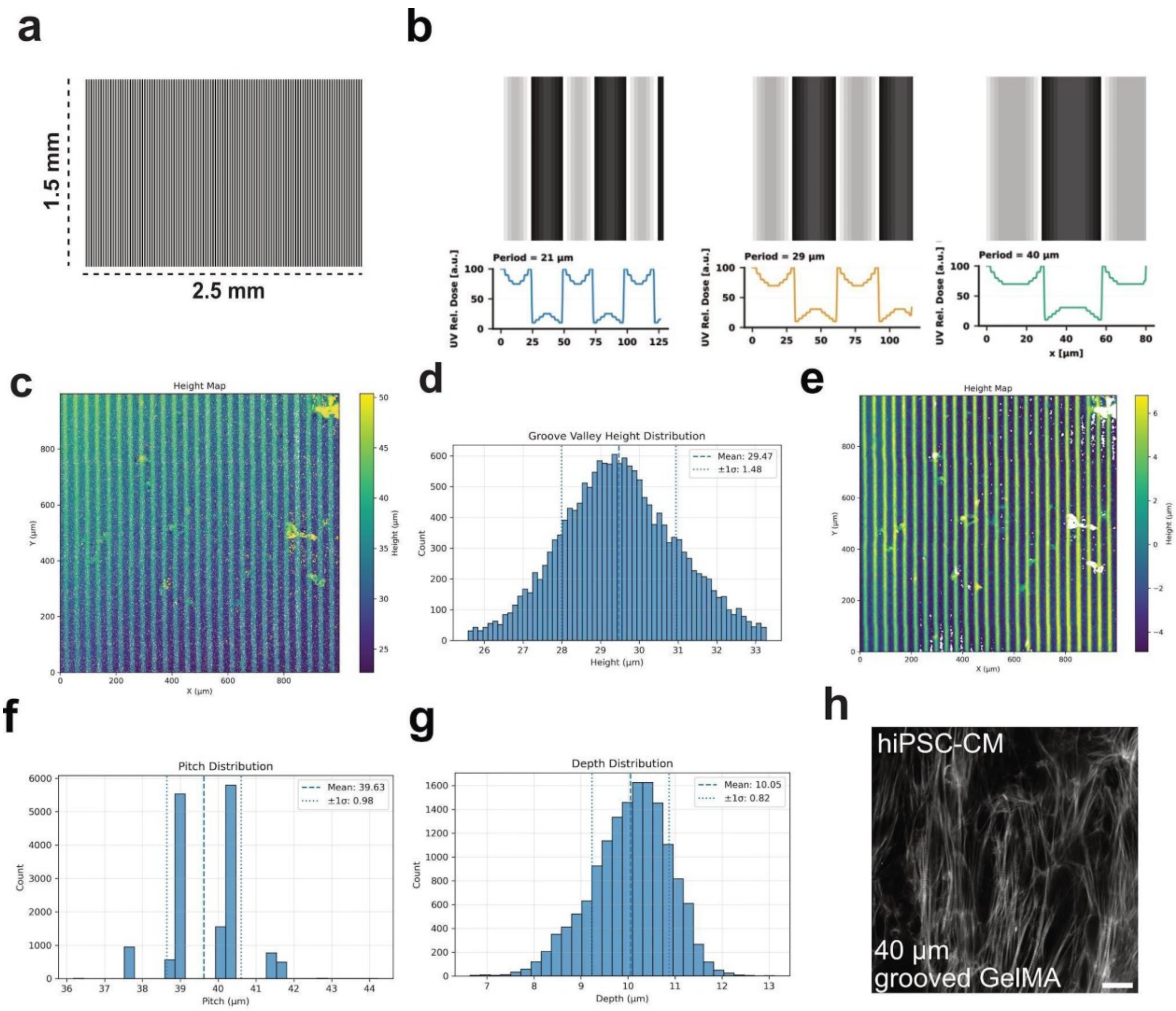

Figure S8. Grayscale DMD designs, automated confocal metrology, and hiPSC-derived cardiomyocytes on PRIMO-patterned grooved hydrogels. a) Line-array design covering the 2.5 mm × 1.5 mm PRIMO printing footprint. b) Grayscale illumination masks for target periods of 21, 29, and 40 µm, each with the corresponding projected relative-dose profile used to verify the programmed spatial period (darker, lower dose; brighter, higher dose). c-g) Confocal metrology of a representative grooved field from the 40 µm pitch condition. c) Raw top-surface height map reconstructed by per-pixel axial intensity peak localization with sub-slice refinement. d) Groove-valley height distribution pooled across the field (mean 29.5 µm, ±1σ 1.5 µm). e) The same field after best-fit plane subtraction and Fourier-guided rotation onto the groove axis, giving the residual topography used for metric extraction. f) Pitch distribution measured as crest-to-crest spacing on aligned, band-limited profiles (mean 39.6 µm, ±1σ 1.0 µm). g) Groove-depth distribution measured as crest-to-adjacent-valleys amplitude across periods, including the pitch-dependent bias correction implemented in groove_analyzer.py (mean 10.1 µm, ±1σ 0.8 µm). Dashed and dotted lines in d, f, and g mark the mean and ±1σ.

Color bars in c and e are in micrometers. h) hiPSC-derived cardiomyocytes seeded at 1 × 10^6 cells mL^{-1} and imaged 5 days later on fibronectin-coated, 40-µm-pitch grooved GelMA, forming a dense monolayer whose F-actin architecture follows the groove axis. F-actin was visualized with a live-cell fluorescent probe; the same monolayer is shown contracting in Video S6. Scale bar: 100 µm. Microscopy acquisition settings are reported in the Main Text Experimental Section.

Note S10. Contact-guided migration acquisition and analysis

Confocal time-lapse acquisition for contact-guided migration imaging: HT1080 migration on grooved and flat substrates was recorded by confocal time-lapse microscopy to quantify cell migration, directional bias relative to the engineered groove axis, and, where indicated, cell morphology and cell-cycle state under conditions compatible with plate-based workflows. Cells were seeded in multiwell formats (384- and 1536-well plates) and imaged using a Crest V3 X-Light spinning-disk confocal microscope (Nikon Ti2) equipped with a Photometrics Kinetix sCMOS camera and a 20x/0.75 NA air objective. Tubulin and actin fluorescence and DIC were acquired sequentially every 15 min for 12 h (4x4 binning; tubulin: 546 nm, 200 ms; actin: 477 nm, 50 ms; DIC: 4 ms). For experiments incorporating cell-cycle reporters, acquisition was extended to four fluorescence channels, comprising two cytoplasmic channels containing the tubulin and actin reporters and two nuclear FUCCIplex reporter channels. DIC, when acquired, was used for visualization but was not used for automated segmentation or tracking. Only fields of view with consistent focus across the full time series and showed no evidence of gel or substrate damage were carried forward for quantitative analysis.

Preprocessing and data handoff to analysis: raw acquisitions were denoised using the NIS-Elements "Denoise" feature and exported from ND2 to TIFF; channels were split via an ImageJ macro to provide single-channel time stacks for downstream analysis. For tracking, the tubulin channel was used as the primary segmentation input to maximize cell-boundary visibility across morphodynamic states and to decouple motility readouts from actin reporters used for phenotyping/visualization.

Cell segmentation (instance masks) from the tubulin channel, trajectory reconstruction (tracking), and track QC (segment_track_stack.py): cell segmentation, trajectory reconstruction, and tracking quality control were performed with a standalone Python script (segment_track_stack.py; code and versioned outputs available through GitHub as stated in

Data Availability). Individual frames were segmented using Cellpose v3.0.5 with the pretrained ‘cyto3’ model. When handling four-channels images, the two cytoplasmic channels acquiring acting and tubulin were combined by taking the pixel-wise maximum of the normalized signals to generate a cytoplasmic image, while the two nuclear channels corresponding to FUCCIplex sensors were combined analogously to generate a nuclear image. These cytoplasmic and nuclear composites were supplied together to Cellpose. For three-channel acquisition datasets, only the tubulin channel was used for segmentation. Segmentation used a user-defined mean cell diameter to account for image scale; for the datasets reported here, a diameter corresponding to 17 pixels was used. This design choice keeps the segmentation step model-driven but scale-aware, improving robustness across wells with minimal manual tuning.

Instance labels were linked into trajectories using LapTrack (v0.17.0), which solves frame-to-frame assignments using tunable distance-based costs. In the provided implementation, linking uses a Euclidean distance metric with conservative cutoffs and limited “memory” to bridge short gaps (parameters exposed in the script), yielding a track table exported as CSV for downstream quantification. To ensure that tracking failures are detectable at scale, the pipeline generates a diagnostic movie overlaying instance boundaries and track IDs on the tubulin signal; this QC output is intended to catch systematic errors (e.g., over-segmentation, missed detections, drift) without interactive curation.

Migration readouts and contact-guidance metric (2D nematic order) with track filtering to avoid unstable or discontinuous trajectories (migration_analysis.py): tracks length control and extraction of migration directionality were performed with a Python script (migration_analysis.py; code and versioned outputs available via GitHub as stated in Data Availability that takes in input the csv file containing information on cell tracking as generated by segment_track_stack.py. To prevent short or fragmented tracks from disproportionately influencing directional statistics, trajectories were filtered before computing migration metrics. Tracks were retained only if they spanned at least 8 frames, and step segments were excluded if the time index contained gaps larger than two frames (i.e., discontinuities exceeding the tolerated linking window). This yields a conservative set of trajectories that better reflect true cell motion rather than intermittent detection artifacts.

Directional bias was quantified on the filtered trajectories using a displacement-weighted nematic order parameter (WNO) referenced to the groove axis. With grooves aligned to the image vertical (y) direction, each step displacement $\Delta r = (\Delta x, \Delta y)$ defines an angle θ relative to the groove axis. The step-level nematic contribution is computed as $s = \cos(2\theta)$, which is equivalently $s = (\Delta y^2 - \Delta x^2)/(\Delta x^2 + \Delta y^2)$; by construction $s = 1$ for perfectly groove-parallel

motion and s = −1 for perfectly groove-perpendicular motion. To ensure that contact guidance reflects directed motion rather than track count or near-zero jitter, step-level contributions are weighted by step length and averaged within each track, and a single FOV value is then obtained as a length-weighted mean across tracks.

Extended morphology and migration analysis: additional cell-shape and motility parameters were extracted from the saved Cellpose label stacks using a Python script (extract_shape_metrics.py; code and versioned outputs available through GitHub as stated in Data Availability). For each segmented object in each frame, the script calculated cell area, perimeter, major- and minor-axis lengths, eccentricity, and major-axis orientation using 'scikit-image' region properties. Object measurements were linked to the corresponding migration trajectory through the shared frame and instance-label identifiers contained in the Cellpose label stack and tracking table. Pixel-based measurements were converted to physical units using the experiment-specific micrometre-per-pixel calibration. Cell aspect ratio was calculated as the major-axis length divided by the minor-axis length, and morphological polarization was quantified such that P=0 represents a circular object and values approaching 1 represent increasingly elongated cells.

The extended analysis additionally calculated instantaneous displacement and velocity, total migration path length, net displacement, mean migration velocity, and persistence ratio, defined as net displacement divided by cumulative path length. Directionality was quantified at the track level using displacement-weighted axis-referenced nematic order as described above. An orientation-independent nematic magnitude was also calculated from the complex second angular moment of the displacement vectors. To assess coupling between cell morphology and migration direction, alignment between the segmented cell major axis and the instantaneous migration direction was quantified using the corresponding twofold angular alignment term, $s=\cos(2\Delta\theta)$. The same track-level exclusion criteria of a minimum of eight observed frames, a maximum temporal gap of two frames, and non-zero cumulative displacement were applied. An exclusion ledger was retained for traceability, and sensitivity analysis was performed by repeating track-level aggregation using minimum track-length thresholds of 5, 8, and 12 frames. Outputs included frame-level cell measurements, track-level metrics, replicate-level summaries, enriched tracking tables, sensitivity-analysis tables, and analysis provenance records.

FUCCIplex cell-cycle analysis from tracked cell masks: for four-channel experiments incorporating FUCCIplex cell-cycle progression reporters, cell-cycle composition was quantified using a Python script (count_fucci_phases_from_labels.py; code and versioned o

available through GitHub as stated in Data Availability). The analysis reused the Cellpose masks generated during migration tracking and went through the corresponding original multichannel stack to measure reporter fluorescence. For each segmented cell, mean fluorescence intensity was measured within the corresponding cell mask for both nuclear reporter channels, and cell-cycle state was assigned according to the dominant reporter signal either as S/G2/M cells or G1 cells. Cells for which both reporters remained below a minimum intensity threshold were retained in the total cell count but left unclassified. Because the analysis uses whole-cell masks rather than independently segmented nuclei, classification relies on the nuclear localization of the FUCCIplex reporters such that the dominant nuclear signal remains dominant when averaged over the corresponding cell mask. FUCCIplex classification was performed at the first, middle, and final frames of each time series by default, with analysis of every frame available when required. For each FOV and time point, the numbers and percentages of G1 and S/G2/M cells were calculated. Replicate-level phase fractions were subsequently aggregated by experimental condition and reported as mean ± SEM. The script generated replicate-level and condition-level phase tables, graphical summaries of FUCCI phase fractions over time.

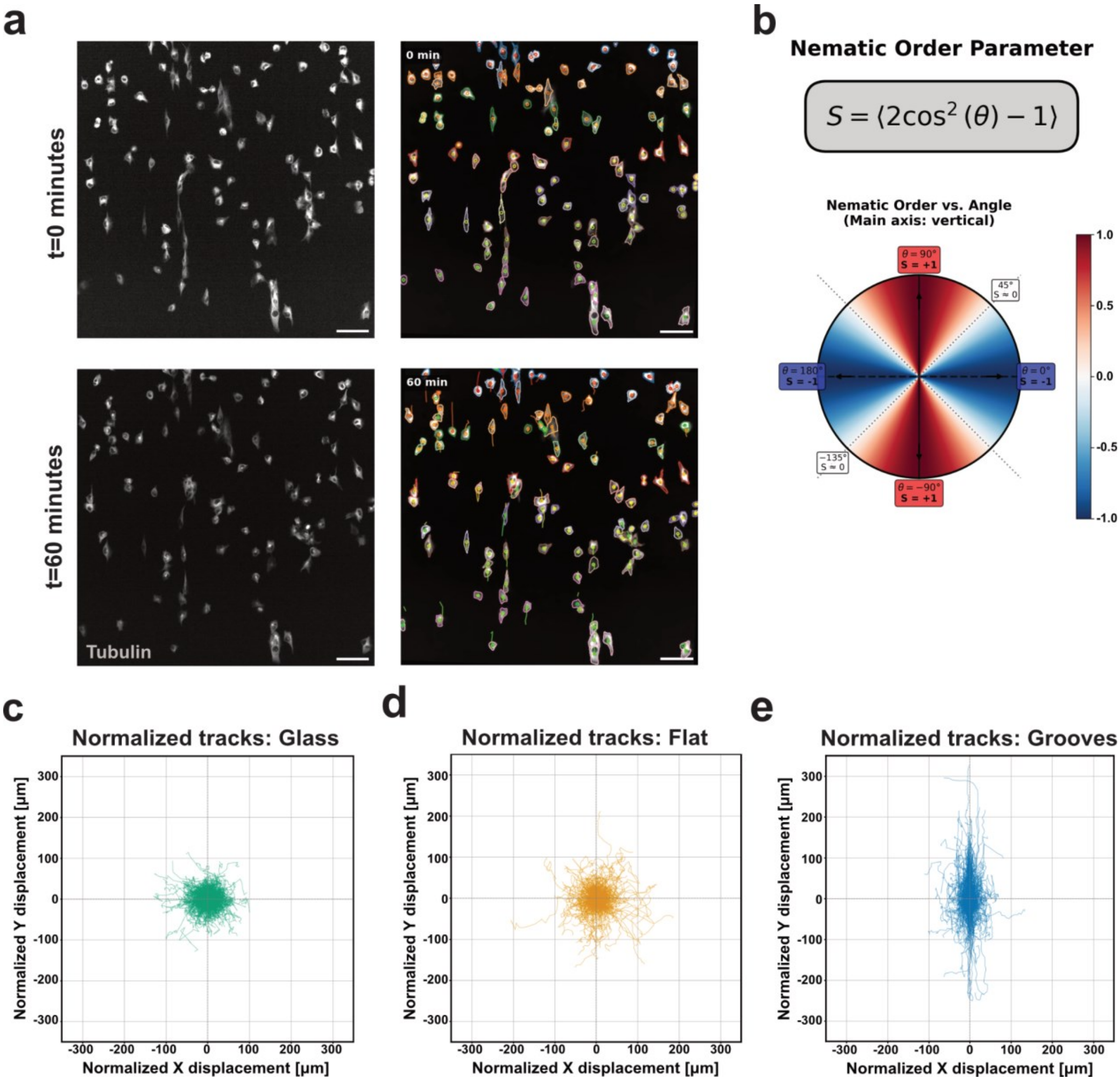

Figure S9. Segmentation/tracking workflow and displacement-weighted nematic order parameter for quantifying contact-guided migration in Figure 4. a) Representative raw confocal tubulin fluorescence frames used for migration analysis (left): first frame (top) and fifth frame (bottom) from the same field of view. Corresponding instance-segmentation masks (right) for the same two frames, labelled t = 0 min (top right) and t = 60 min (bottom right), with the t = 60 min mask additionally overlaid with the linked LapTrack trajectories used for downstream trajectory aggregation. Scale bars: 100 µm. b) Definition and codomain of the nematic order parameter used to collapse the circular migration-angle variable into a scalar alignment score. The angle θ in the printed expression is measured between the instantaneous displacement and the groove axis, so the score is +1.0 for displacement parallel to the reference axis (main/groove axis defined as vertical) and −1.0 for displacement orthogonal to it. The direction labels around the polar map are given relative to the image horizontal axis. The polar map illustrates the mapping from direction to aligned motion, orthogonal motion, and motion

at 45° with respect to the reference axis, with the color bar indicating the scalar range. c-e) Origin-normalized single-cell migration tracks (each trajectory translated to start at the origin) for cells migrating on c) fibronectin-coated glass, d) flat GelMA gels, and e) grooved GelMA gels. Tracks are plotted normalized in a common coordinate frame (groove axis vertical for the patterned condition) to enable direct visual comparison of ensemble directionality; at least N=85 tracks are reported in each plot.

Note S11. Compatibility with hiPSC-derived cardiomyocytes

WTC11 hiPSCs (hPSCreg UCSFi001-A-1C) engineered with fluorescent probes for actin and calcium were maintained on growth factor-reduced Matrigel (1:100) in Essential 8 Flex medium supplemented with penicillin-streptomycin, and passaged with 0.5 mM EDTA and RevitaCell. Cardiomyocytes were obtained by small-molecule direct differentiation as described previously, specifically through induction with CHIR99021 (7.5 µM) on day 0 and Wnt-C59 (2 µM) on day 2, switching to maintenance medium on day 6, and metabolic purification from day 12 with 4 mM sodium DL-lactate.

Purified cardiomyocytes were reseeded at 1 × 10^6 cells mL^{-1} on 40-µm-pitch grooved hydrogels prepared from 150 mg mL^{-1} GelMA precursor and coated beforehand with human plasma fibronectin (40 µg mL^{-1} in PBS, 1 h at room temperature), as for the keratinocyte experiment. After 5 days the cells had formed a dense monolayer whose F-actin architecture followed the groove axis across the field of view (Figure S8h), and the monolayer contracted spontaneously and synchronously (Video S6). Alignment was partial rather than complete, as expected for a monolayer seeded at high density on grooves of this pitch without additional patterning cues.

Note S12. LigHTS beyond GelMA: compatibility with HAMA and PEGDA hydrogel photostructuring

We used the same preparation protocol with lyophilized HAMA (lot MKCZ1205, product code 943118, Merck) and neat PEGDA solution (lot MKCX1004, product code 437441, Merck). Specifically, we dissolved low-molecular-weight HAMA (10-20 kDa) in PBS to obtain the following solution: 75 mg mL^{-1} HAMA, 1 mg mL^{-1} LAP, and 4.5 mg mL^{-1} tartrazine and photopatterned it using 40-µm groove design at UV doses of 8, 13, and 21 mJ mm^{-2}. We tested higher molecular weight HAMA (100-150 kDa, product number 5212, CellInk) for preparing

additional hydrogels, but the viscosity at the tested concentration was not compatible with room temperature casting by air-displacement pipetting.

Precursor solution for PEGDA hydrogels were instead prepared containing 7.5% (w/w) PEGDA (575 Da), 2.5 mg mL^{-1} LAP, and 4.5 mg mL^{-1} tartrazine and patterned using groove designs of 21, 29, and 40 µm pitch at a fixed dose of 13 mJ mm^{-2}, reproducing the pitch-to-depth dependence measured for GelMA.

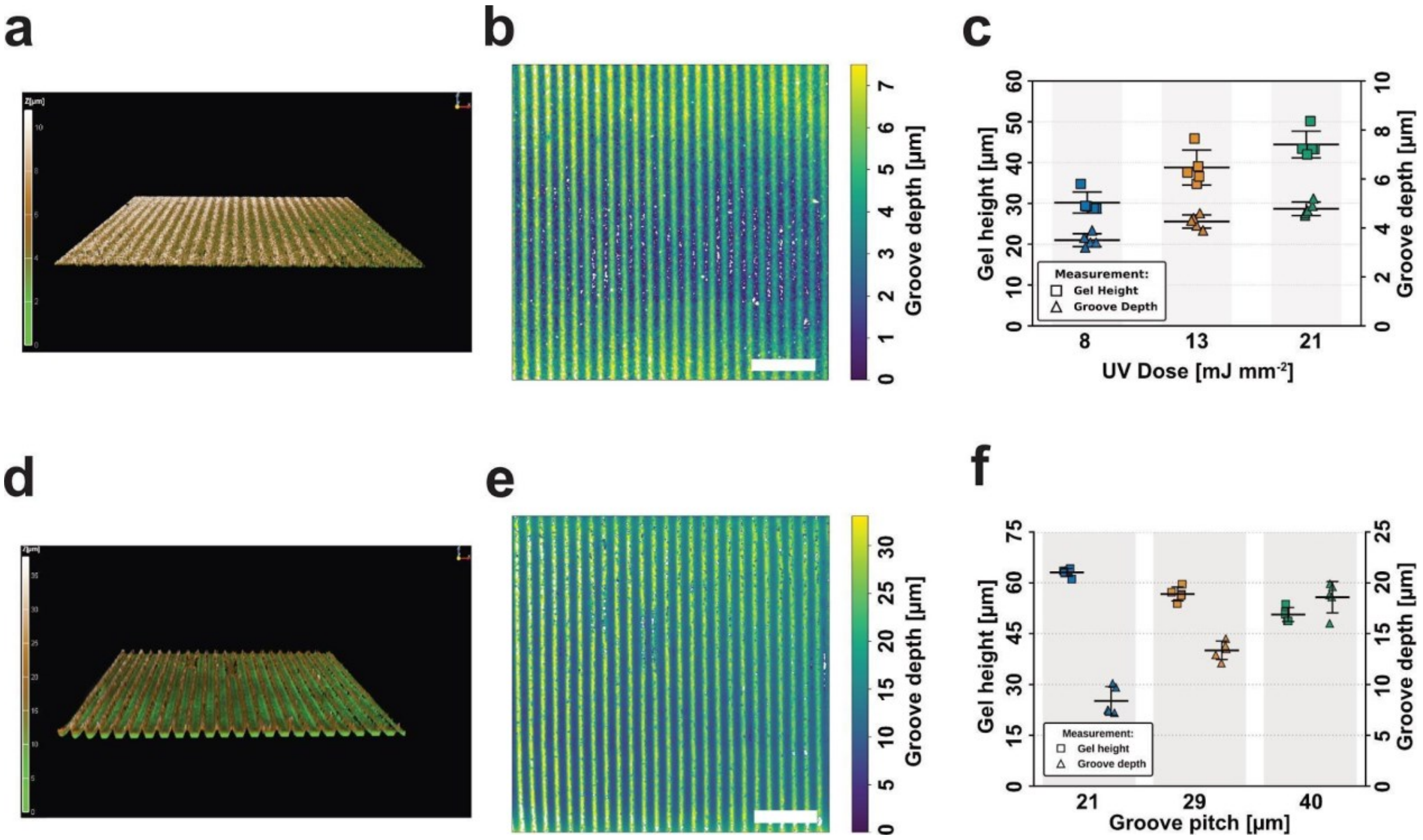

Figure S10. Absorber-guided photostructuring extends to HAMA and PEGDA hydrogels. a-c) HAMA hydrogels (75 mg mL^{-1} HAMA of 10-20 kDa, 1 mg mL^{-1} LAP, 4.5 mg mL^{-1} tartrazine) patterned with a fixed groove design at increasing UV dose. a) Representative 3D rendering of the reconstructed surface. b) Groove-depth map of the same field. c) Gel height (squares, left axis) and groove depth (triangles, right axis) at 8, 13, and 21 mJ mm^{-2}. Both increase with dose, by 47% and 37% respectively, while the absolute change is 14.2 µm in height against 1.3 µm in depth. d-f) PEGDA hydrogels (7.5% w/w PEGDA of 575 Da, 2.5 mg mL^{-1} LAP, 4.5 mg mL^{-1} tartrazine) patterned at a fixed dose of 13 mJ mm^{-2} through groove designs of increasing pitch. d) Representative 3D rendering. e) Groove-depth map. f) Gel height (squares, left axis) and groove depth (triangles, right axis) at 21, 29, and 40 µm pitch; height falls by 20% while groove depth rises by 121%. Each point is one well, five wells per condition from a single plate, and horizontal bars denote mean ± SD. Scale bars in b and e: 200 µm.

Note S13. Optics-only UV diffraction model for photomask-mediated dose modulation

We simulated the one-dimensional irradiance modulation at the hydrogel plane produced by a periodic photomask using a scalar, optics-only diffraction model. The photomask was approximated as an infinite, binary amplitude line grating with 50% duty cycle. The complex field immediately after the mask was expanded into spatial harmonics of the grating period (diffraction orders), and each harmonic was propagated a distance z using the scalar Helmholtz propagator exp(i kz z), where $k = 2\pi/\lambda$ and $kz = \text{sqrt}(k^2 - kx^2 - ky^2)$. The propagated harmonics were coherently summed to obtain E(x,z), and the irradiance was computed as $I(x,z) = |E(x,z)|^2$. Finite source divergence was treated as angular incoherence: I(x,z) was computed for a deterministic set of plane-wave incidence directions within a cone of half-angle θ and then averaged (incoherent sum) with approximately uniform solid-angle weighting. The illustrative profiles in Figure 5b use λ = 365 nm, z = 200 µm, and grating pitches of 40, 60, and 80 µm. Intensities are reported on a relative (arbitrary) scale and are not radiometrically calibrated; values greater than 1 can occur due to constructive interference. The model omits photopolymerization kinetics and material response, refractive-index effects, absorption or scattering, and finite mask-thickness and reflection effects, and is intended to capture the qualitative smoothing of a binary mask into a near-sinusoidal dose modulation under finite divergence (Figure 5b).

Note S14. Preprocessing of holographic phase imaging datasets and groove metrology

Scope and relationship to the confocal workflow: this note describes the holographic-only preprocessing and groove quantification used for Figure 6e-f. It is intentionally complementary to the confocal z-stack groove metrology note: holographic phase imaging begins from an instrument-reconstructed phase/height map (no axial peak localization), and because acquisition is centered within wells, it does not require the well-ROI segmentation logic used in plate-wide confocal QC.

Data export and height-map preparation (holo_to_tiff.py): holographic acquisitions were exported from HoloMonitor AppSuite as per-frame quantitative phase/height images. The preprocessing script (holo_to_tiff.py; code available via GitHub as stated in Data Availability) performs several sequential operations that replace the confocal-specific steps used in the groove analysis workflows. First, all frames belonging to the same field of view are grouped

into a single stack. Second, frame-to-frame rigid registration is applied for drift correction: within each field-of-view stack, frames are aligned to the first frame by FFT-based phase correlation (subpixel translation via cv2.phaseCorrelate) after groove-axis rotation, using the intersection of valid-pixel masks to exclude NaN-valued pixels; the resulting per-frame shift values (dx, dy, and correlation response) are stored in the output TIFF metadata for auditability. Third, phase values are decoded: per-frame exports encoded as phase-coded 16-bit TIFFs (carrying per-image phase extrema in the ImageDescription tag) are mapped by linear interpolation to native AppSuite phase units (one unit corresponds to one wavelength of optical path) and stored as 8-bit maps on a fixed, uncalibrated scale (15.8 µm per native unit) that is written to the output TIFF metadata and undone exactly before the calibrated measurement (Note S15). Fourth, geometric normalization for groove-aligned metrology is performed: a preliminary frequency-domain estimate of groove orientation is used to rotate maps so that grooves are axis-aligned before one-dimensional profiling, reducing the sensitivity of peak/valley detection to arbitrary camera rotation and well-to-well placement. Fifth, a consistent region of interest is defined by selecting a common, NaN-free central crop shared across all registered frames for a given field of view. Finally, a median-based temporal reduction over the successfully registered frames (phase-correlation response ≥ 0.3; fields with fewer than three such frames are excluded from Figure 6f) is computed to yield a representative two-dimensional height map for groove quality control while minimizing the influence of intermittent reconstruction voids at field-of-view boundaries; both the median height map and the full frame stack are saved for subsequent analysis. These steps produce per-well TIFF median-based height maps that are directly consumable by the groove analysis script below.

Groove analysis from holographic height maps (groove_analyzer_holo.py): groove pitch and depth are extracted from each TIFF height map using a Python script designed for periodic microtopographies and robust to missing pixels (NaNs) typical of phase reconstruction at boundaries (groove_analyzer_holo.py code and versioned outputs available via GitHub as stated in Data Availability). Each TIFF is loaded as either a 2D height map or a 3D stack (time series). When a stack is provided, a per-pixel temporal median is computed to obtain a representative 2D map. To isolate the periodic relief from residual sample tilt and low-order curvature, a best-fit plane is estimated within the valid-pixel mask and subtracted. A 2D FFT of the detrended map is then used to identify the dominant spatial frequency and its orientation; the map is rotated so grooves are axis-aligned and a centered NaN-free crop is selected to avoid edge artifacts from interpolation and reconstruction voids. A 1D height profile is formed by averaging across the groove direction (ignoring NaNs) and is band-limited to suppress slow

curvature and high-frequency noise before peak/valley detection. Pitch is computed as the mean crest-to-crest spacing from detected peaks, and depth is computed crest-by-crest as the crest height minus the mean of the two nearest bracketing valley heights.

Uncertainty model and QC flags: For each well, the script reports uncertainty using a GUM-style split into Type-A (repeatability) and Type-B (resolution/calibration) components. Type-A terms are derived from the dispersion of per-groove (and/or per-row) measurements within the analyzed FOV. Type-B terms capture finite sampling and quantization, using the lateral pixel size for pitch resolution and the effective height quantization step for depth. QC descriptors (e.g., valid-pixel fraction, FFT pitch-seeding success, and number of detected grooves) are exported per file so that wells failing seeding or returning too few valid grooves can be flagged and excluded reproducibly.

Analysis parameters for holographic groove metrology (e.g., FFT pitch search band, detrending/rotation logic, peak/valley detection thresholds, and uncertainty components tied to pixel size and height quantization) are held fixed within a dataset and are implemented directly in groove_analyzer_holo.py. For traceability, the script exports per-file QC descriptors (e.g., valid-pixel fraction, pitch-seeding success, and number of detected grooves) alongside pitch/depth values and uncertainties, enabling reproduction of any downstream exclusions directly from the exported tables and the analysis code. These crest-to-valley depths serve quality control only; the physical depths in Figure 6f are measured with the estimator of Note S15 and converted with C.

Plate-level summaries for Figure 6e-f: Figure 6e shows a representative single-field holographic map. For Figure 6f, the 382 plate fields were decoded to native phase and measured with the estimator of Note S15; two fields matched no design pitch, and 375 fields passed the pre-declared quality control (at least three registered frames, pitch within 10% of nominal, at least four periods, residual RMS $\leq 0.6 \times$ depth). Physical depths $d = C\varphi$ were grouped by nominal pitch (mean ± SD) and compared with the confocal group means converted with ζ, using the expanded uncertainty ($k = 2$) of the difference as acceptance criterion.

Note S15. Cross-calibration of confocal and holographic groove metrology against nanoindentation

Neither confocal z-stacking nor holographic phase imaging reports a physical groove depth directly. Confocal z-stacking returns an apparent optical depth a, because a dry NA 0.75

objective focused into an aqueous gel compresses the axial scale. Holographic phase imaging returns a phase contrast φ, expressed in the native phase units of the HoloMonitor AppSuite reconstruction (one unit corresponds to one wavelength of optical path, i.e. an optical path difference OPD = φλ with λ = 635 nm), which becomes a height only through the refractive-index contrast of the swollen network. Nanoindentation contacts the surface and reports the relief directly, so we used it as the physical anchor and defined d = ζa and d = Cφ. One 40-μm-pitch, 75 mg mL^{-1} GelMA specimen, DMD-patterned as described for Figure 4, was measured by all three modalities in PBS at 37 °C: a 40 × 40 nanoindentation map at 7 μm step acquired in peak-force mode with a 3.5 μm tip radius (1600 curves), three confocal z-stacks, and five holographic fields of seven frames (Figure S11a-c). The modalities could not be registered to a common location, so every comparison is between field-averaged quantities on that specimen.

Because a calibration constant is the ratio of two measurements, any difference in estimator between the modalities propagates directly into it. Every field of every modality on the calibration specimen, and every holographic plate field in Figure 6f, was therefore processed with a single estimator. Groove orientation was taken from the two-dimensional FFT and refined by maximizing the contrast of the collapsed profile. The profile was then formed as a 10% trimmed mean along the grooves, detrended with a quadratic across them, and fitted with six harmonics at the refined pitch, reporting the peak-to-trough amplitude of the fundamental. Plate confocal depths were taken from the confocal groove pipeline (Note S9).

The specimen gave a physical depth of 23.6 ± 0.7 μm, an apparent confocal depth of 15.6 ± 1.2 μm, and a phase contrast of 0.73 ± 0.04, hence ζ = 1.5 ± 0.1 and C = 32 ± 2 μm per native phase unit (Figure S11d-f), which corresponds to an effective gel-to-medium refractive-index contrast Δn_eff = λ/C ≈ 0.020 (gel index ≈1.35). ζ can also be bounded from optics alone: for a dry objective focused into a medium of index 1.35 the paraxial and marginal-ray limits are 1.35 and 1.70, with the wave-optical estimate at 1.47,[60] and the measured value falls inside that band (Figure S11e).

The ratio Λ = C/ζ = a/φ carries neither the mechanical anchor nor the phase convention, so it tests whether the calibration specimen and the plates form the same optical system. Measuring the 382 plate fields with the same estimator (375 passed quality control) gave an anchor value of Λ = 21.1 ± 0.7 μm per native phase unit from the 60 and 80 μm groups, which the independently calibrated specimen (Λ = 21.3 ± 2.0 μm per native phase unit) reproduces to

within 1%. With the locked constants, holographic and confocal depths in Figure 6f agree to within 4% at 60 and 80 µm pitch, inside expanded tolerances of 20% and 19% (k = 2). The 40 µm group sits about 19% below the anchor, a deviation that no choice of ζ or C can remove and that we attribute to the shallow apparent confocal depth at this pitch (≈6 µm, about seven 0.9 µm z-steps), where the confocal measurement uncertainty is largest; the corresponding depths differ by +25%. Depths at 40 µm therefore carry an additional systematic error and are reported as indicative.

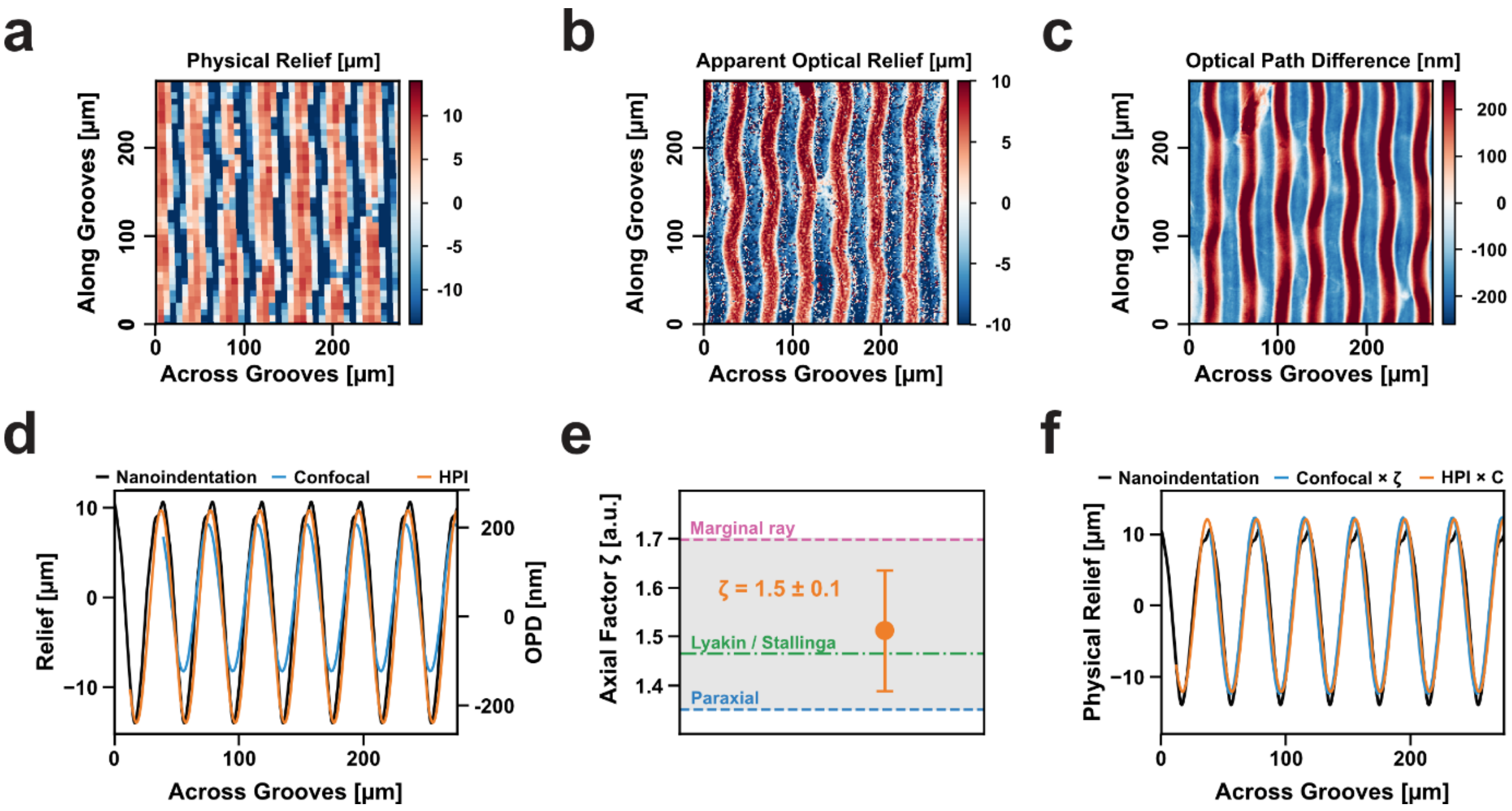

Figure S11. Cross-calibration of confocal and holographic groove metrology against nanoindentation. a–c) One 40-µm-pitch, 75 mg mL^{-1} GelMA specimen measured in PBS at 37 °C by the three modalities, each shown in the quantity it reports: a) physical relief extracted from nanoindentation contact point map, b) apparent optical relief from confocal z-stacking, c) optical path difference from holographic phase imaging (OPD = φλ). d) Comparison between phase-aligned field-averaged profiles, each on its native scale. e) Estimated axial factor ζ using nanoindentation groove depth as ground truth for transforming the optical thickness from the confocal pipeline into physical groove depth. The estimated value is plotted against the paraxial and marginal-ray limits for a dry NA 0.75 objective compared to literature and theory estimates and ranges. f) Comparison of estimated physical groove depths of the three characterization methods, after conversion with ζ and with the phase-to-relief coefficient C.

Note S16. Holographic imaging for cell migration analysis

For holographic migration imaging (Video S8), per-FOV time series exported by holo_to_tiff.py were processed to isolate moving cells from the static groove background. Within each FOV stack, a temporal median frame was computed and manually subtracted from every time point to suppress the stationary topography signal and emphasize cell motion. For visualization, the median groove map and the median-subtracted cell stack were false-colored (cyan and magenta, respectively) prior to movie export. The median-subtracted stacks were then analyzed using the same segmentation and tracking workflow described above for confocal migration (Cellpose + LapTrack), with the segmentation diameter adjusted to 30 px to match the holographic pixel scale.

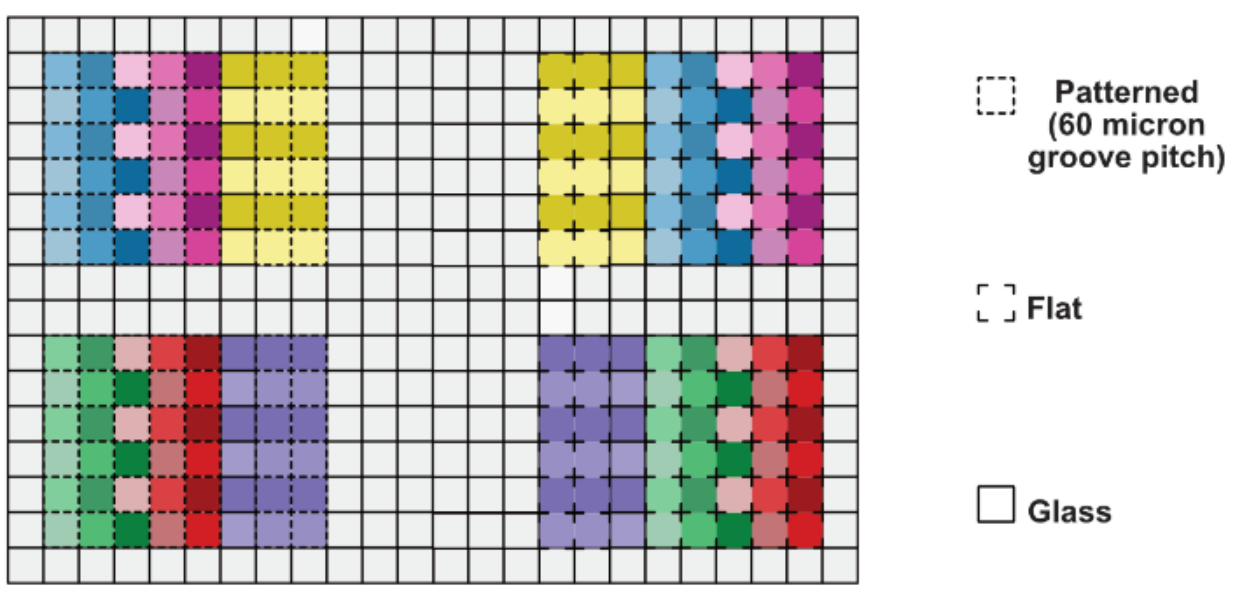

Drug		Concentration [µM]				
		1.	2.	3.	4.	5.
PP2	P	0.1	0.3	1	3	10
Mibefradil	M	0.3	1	3	5	10
Nocodazole	N	0.01	0.03	0.1	0.3	1
Y27632	Y	0.3	1	3	10	30

1:1000 DMSO (vehicle)
1:1000 DMSO + FBS (vehicle + FBS)
5 µM CytoD (positive control)
5 µM CytoD + FBS (positive control + FBS)

Figure S12. Schematics of the experimental design for the proof-of-concept multiparametric pharmacological perturbation assay on flat and grooved LigHTS substrates in a 384-well plate. HT1080 were seeded in a 384-well plate in wells featuring 60-µm-pitch grooved hydrogels (dashed well outline) or flat hydrogels (corner-outlined wells), as well as non-hydrogel-functionalized wells. 24 hours after seeding, the fetal bovine serum (FBS) present at 10% v/v concentration was removed from the media. After 24 hours of serum starvation, cells on gels were treated with PP2 (P), mibefradil (M), nocodazole (N), or Y-27632 (Y) in FBS-free media, with each of the drugs at the five tabulated concentrations, with three wells per combination of drug concentration and gel surface geometry. Additional wells with HT1080 cells on hydrogels and on glass substrates were imaged while under vehicle (DMEM/F12, 1% P/S, 1:1000 DMSO) with and without serum as internal technical control for viability and cell growth, as well as with 5 µM cytochalasin D as positive control for migration inhibition.
